# Promoting photocatalytic CO_2_ reduction through facile electronic modification of N-annulated perylene diimide rhenium bipyridine dyads†

**DOI:** 10.1039/d1sc05465a

**Published:** 2021-12-28

**Authors:** Josh D. B. Koenig, Warren E. Piers, Gregory C. Welch

**Affiliations:** Department of Chemistry, University of Calgary 2500 University Drive N.W. Calgary Alberta T2N 1N4 Canada joshua.koenig1@ucalgary.ca gregory.welch@ucalgary.ca

## Abstract

The development of CO_2_ conversion catalysts has become paramount in the effort to close the carbon loop. Herein, we report the synthesis, characterization, and photocatalytic CO_2_ reduction performance for a series of N-annulated perylene diimide (NPDI) tethered Re(bpy) supramolecular dyads [Re(bpy-C2-NPDI-R)], where R = –H, –Br, –CN, –NO_2_, –OPh, –NH_2_, or pyrrolidine (–NR_2_). The optoelectronic properties of these Re(bpy-C2-NPDI-R) dyads were heavily influenced by the nature of the R-group, resulting in significant differences in photocatalytic CO_2_ reduction performance. Although some R-groups (*i.e.* –Br and –OPh) did not influence the performance of CO_2_ photocatalysis (relative to –H; TON_co_ ∼60), the use of an electron-withdrawing –CN was found to completely deactivate the catalyst (TON_co_ < 1) while the use of an electron-donating –NH_2_ improved CO_2_ photocatalysis four-fold (TON_co_ = 234). Despite being the strongest EWG, the –NO_2_ derivative exhibited good photocatalytic CO_2_ reduction abilities (TON_co_ = 137). Using a combination of CV and UV-vis-nIR SEC, it was elucidated that the –NO_2_ derivative undergoes an *in situ* transformation to –NH_2_ under reducing conditions, thereby generating a more active catalyst that would account for the unexpected activity. A photocatalytic CO_2_ mechanism was proposed for these Re(bpy-C2-NPDI-R) dyads (based on molecular orbital descriptions), where it is rationalized that the photoexcitation pathway, as well as the electronic driving-force for NPDI^2−^ to Re(bpy) electron-transfer both significantly influence photocatalytic CO_2_ reduction. These results help provide rational design principles for the future development of related supramolecular dyads.

## Abstract 2

Seven N-annulated perylene diimide tethered rhenium (2,2′-bipyridine) supramolecular dyads are evaluated as photocatalysts for the reduction for carbon dioxide, highlighting the importance of photoexcitation pathway and electronic driving-force.

## Graphical abstract

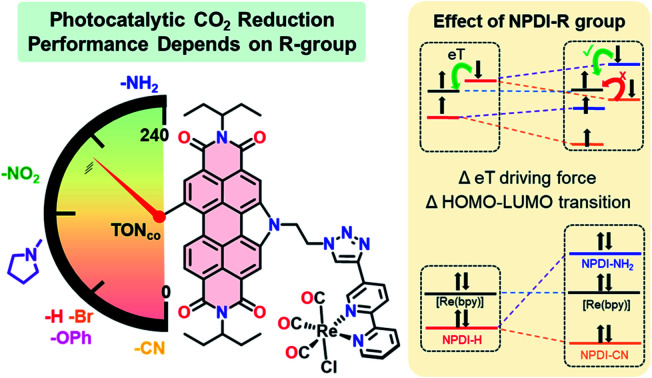

## Introduction

The adverse effects on climate change related to increased anthropogenic CO_2_ emissions has inspired the utilization of excess CO_2_ as a sustainable feedstock for value-added chemicals and fuels.^1,2^ While the activation of CO_2_ is kinetically unfavorable, it can be readily accomplished electro-/photocatalytically *via* proton-couple multielectron chemical reductions.^3,4^ Consequently, the development of capable molecular electro-/photocatalysts has mainly focused on improving the efficiency and selectivity of the CO_2_ conversion process.^5–9^ Among the many comprehensively studied molecular catalyst systems, Re(2,2′-bipyridine)(CO)_3_Cl [Re(bpy)] is notable for its highly selective CO_2_-to-CO conversion.^10^ The versatile bpy ligand has been modified with a variety of substituents to change both the electronic properties and/or the second-sphere H-bonding character of the catalyst.^11–18^ And while Re(bpy) alone can be used as an effective CO_2_ reduction photocatalyst,^19–24^ the photocatalytic CO_2_ reduction performance is greatly enhanced *via* the direct functionalization of Re(bpy) with photosensitizing (PS) units.^25–31^

The development of ruthenium(ii) diimine photosensitized Re(bpy) supramolecular dyads has been extensively reported by the Ishitani group.^5,25–31^ These Ru^II^–Re^I^ dyads make use of a Z-scheme architecture whereby the photoexcited electrons of the Ru^II^-moiety are reductively quenched and subsequently transferred to the Re^I^ catalyst center to enable CO_2_ reduction. To facilitate efficient electron-transfer (eT) and CO_2_ photocatalysis, several supra-molecular dyad design principles have been established. First, the photoexcited electron should be localized near the tethering portion between the PS and the catalyst.^5^ Second, the tether between the PS and catalyst moieties should be as short as possible (without being through-conjugated) to enable rapid intramolecular eT.^27–30^ Third, increasing the molar absorptivity of the PS-moiety (*i.e.* by incorporating multiple PS units) can improve the quantum efficiency capabilities and the ensuing eT dynamics of the supramolecular dyad.^31^ Restricted by the first two design principles, attempts to improve the quantum efficiency of these Re(bpy) dyads has been made by using more strongly absorbing PS units, such as porphyrins,^32–37^ naphthalimide,^38,39^ naphthalene diimide,^40–42^ and perylene diimide (PDI).^43,44^ Although the photophysical dynamics of these dyads appear fundamentally well-understood, only a handful have been properly evaluated as CO_2_ reduction photocatalysts (see ESI, Table S1†).^35–39^

**Fig. 1:**
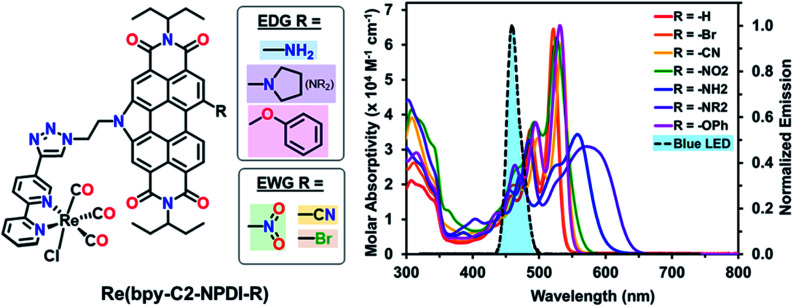
(Left) chemical structures and (right) UV-vis absorption spectra of Re(bpy-C2-NPDI-R) dyads in CHCl_3_ (∼10^−5^ M), where R = –H (red), R = –Br (orange), R = –CN (yellow), R = –NO_2_ (green), R = –NH_2_ (blue), R = –NR_2_ (purple), and R = –OPh (magenta). The optical profiles of all Re(bpy-C2-NPDI-R) dyads overlap with the emission spectrum of the blue LED (*λ* = 470 ± 30 nm; 4 mW cm^−2^) used for photocatalytic CO_2_ reduction testing.

Recently, we reported on four N-annulated perylene diimide (NPDI) functionalized Re(bpy) dyads as CO_2_ reduction electro-catalysts.^45^ Our investigation of these Re(bpy)–NPDI dyads revealed that the PS unit (NPDI) functions as an electron-reservoir for Re(bpy), enabling efficient CO_2_ reduction at an overpotential 300 mV lower than conventional Re(bpy)-type electrocatalysts. Moreover, it was also elucidated that the tether length between Re(bpy) and NPDI governs which CO_2_ reduction mechanism is preferred for the supramolecular dyad(s), where the ethyl-linked Re(bpy)–NPDI dyad possessed the greatest degree of electronic communication. These promising results from our initial Re(bpy)–NPDI dyads led us to hypothesize that eT from the electron-reservoir to the Re(bpy) catalyst could be improved by electronically modifying NPDI in two different ways. It was theorized that the introduction of electron withdrawing groups (EWGs) on NPDI may inductively stabilize the entire dyad, thus enabling more efficient eT by increasing the overall electron affinity of Re(bpy)-moiety. Alternatively, the use of electron donating groups (EDGs) on NPDI could make the electron-reservoir more electron-rich and thus more willing to transfer electrons to the Re(bpy)-moiety. To determine which of these two opposing hypotheses was correct, a series of electronically modified ethyl-linked Re(bpy)–NPDI dyads [Re(bpy-C2-NPDI-R)] (where R = –H, –Br, –CN, –NO_2_, –OPh, –NH_2_, or –NR_2_) were designed (Fig. 1, left) and, for the first time, their photocatalytic CO_2_-to-CO reduction performance was evaluated. It was revealed that installing an EDG, such as –NH_2_, led to a four-fold enhancement in turnover numbers of CO (TON_co_), with respect to the benchmark Re(bpy-C2-NPDI-H) dyad. A mechanism based on molecular orbital (MO) energy levels is proposed to explain the observed differences in photocatalytic CO_2_ reduction performance for these dyads caused by the installation of EWGs and EDGs on NPDI.

## Results & discussion

### Synthesis & characterization

**Scheme 1:**
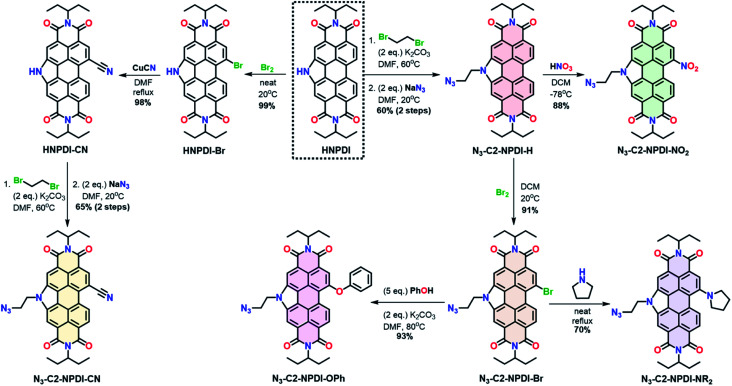
Synthesis of N_3_-C2-NPDI-R precursors that are converted into Re(bpy-C2-NPDI-R) dyads.

The synthesis of all azide-ethyl-NPDI precursors (N_3_-C2-NPDI-R) starts from the HNPDI synthon (Scheme 1).^47–49^ The pyrrolic nitrogen was alkylated with 1,2-dibromoethane and a terminal azide was installed using S_N_2 chemistry to give N_3_-C2-NPDI-H.^45,47^ Treatment of N_3_-C2-NPDI-H with either fuming HNO_3_ (at −78 °C) or Br_2_ (at 20 °C) affords the N_3_-C2-NPDI-NO_2_ and N_3_-C2-NPDI-Br precursors, respectively. When refluxed with pyrrolidine, the N_3_-C2-NPDI-Br precursor can be converted to N_3_-C2-NPDI-NR_2_. Alternatively, reacting N_3_-C2-NPDI-Br with an excess of phenol and K_2_CO_3_ in *N*,*N*-dimethylformamide (DMF; at 80 °C) generates N_3_-C2-NPDI-OPh. Attempts to cyano-functionalize N_3_-C2-NPDI using the Rosenmund–von Braun reaction conditions resulted in the elimination of the terminal azide. Consequently, N_3_-C2-NPDI-CN had to be synthesized starting from HNPDI. After selectively brominating HNPDI,^50^ HNPDI-Br was reacted with excess CuCN in refluxing DMF to yield HNPDI-CN. This intermediate was then alkylated and azide-functionalized to give the N_3_-C2-NPDI-CN precursor.

Next, these six N_3_-C2-NPDI-R precursors were linked to Re(5-ethynyl-2,2′-bipyridine)(CO)_3_Cl [Re(ethynyl-bpy)] using a standard copper catalyzed azide–alkyne cycloaddition (CuAAC) procedure (see ESI Section II for more details†).^45,47^ While most Re(bpy-C2-NPDI-R) dyads (where R = –H, –Br, –CN, –NR_2_, and –OPh) were obtained in excellent yields as the exclusive product, the CuAAC reaction between N_3_-C2-NPDI-NO_2_ and Re(ethynyl-bpy) generated both Re(bpy-C2-NPDI-NO_2_) (major product) and Re(bpy-C2-NPDI-NH_2_) (minor product). To obtain each catalyst selectively, two additional CuAAC protocols were developed. The selective synthesis of Re(bpy-C2-NPDI-NO_2_) was accomplished by changing the catalyst from CuSO_4_ (with sodium ascorbate) to CuI, suggesting that the substoichiometric sodium ascorbate used under our standard CuAAC conditions acted as a reducing agent.^51–53^ With this insight, Re(bpy-C2-NPDI-NH_2_) was afforded exclusively using catalytic CuSO_4_ and a stoichiometric excess of sodium ascorbate. The identity of all Re(bpy-C2-NPDI-R) dyads was confirmed using ^1^H and ^13^C NMR spectroscopies, as well as MALDI-TOF mass spectrometry and CHN elemental analysis (Fig. S1–S54†). Note, each specific Re(bpy-C2-NPDI-R) dyad will henceforth be referred to by their R-group only (where R = –H, –Br, –CN, –NO_2_, –NH_2_, –NR_2_, and –OPh).

The optical properties of these Re(bpy-C2-NPDI-R) dyads were probed using both UV-visible-near-infrared (UV-vis-nIR) absorption and Fourier transform infrared (FTIR) spectroscopies. Visually, the optical properties of all Re(bpy-C2-NPDI-R) dyads were dominated by the NPDI-moiety (Fig. 1 and S55–S56†). Relative to –H (*λ*_max_ = 521), the installation of –Br, –CN, –NO_2_, and –OPh groups on NPDI all caused a minor bathochromic shift of the absorption profile (Δ*λ*_max_ = 1–13 nm) while maintaining most of the vibronic transitions of the parent complex. As for the –NH_2_ and –NR_2_ dyads, the optical spectra were significantly red-shifted (*λ*_max_ = 558 and 572 nm, respectively) and the molar absorptivity also decreased (from ∼60 000 to ∼40 000 M^−1^ cm^−1^). The broadened absorption of –NH_2_ and –NR_2_ near the *λ*_max_ is consistent with the donation of electron-density from the lone-pair of the amino nitrogen into the NPDI π-system.^54–56^ Notably, all dyads exhibit an absorption peak around *λ* = 341–343 nm, characteristic of a Re(bpy)-based metal-to-ligand charge transfer band.^57^ By FTIR, all Re(bpy-C2-NPDI-R) dyads possessed three carbonyl stretching frequencies (*v*_co_ ∼1900, 1925, and 2025 cm^−1^), which is consistent with other Re(bpy) catalysts (Fig. S57†).^11–18,58^ Together, these data confirm successful Re(bpy-C2-NPDI-R) dyad formation.

**Fig. 2:**
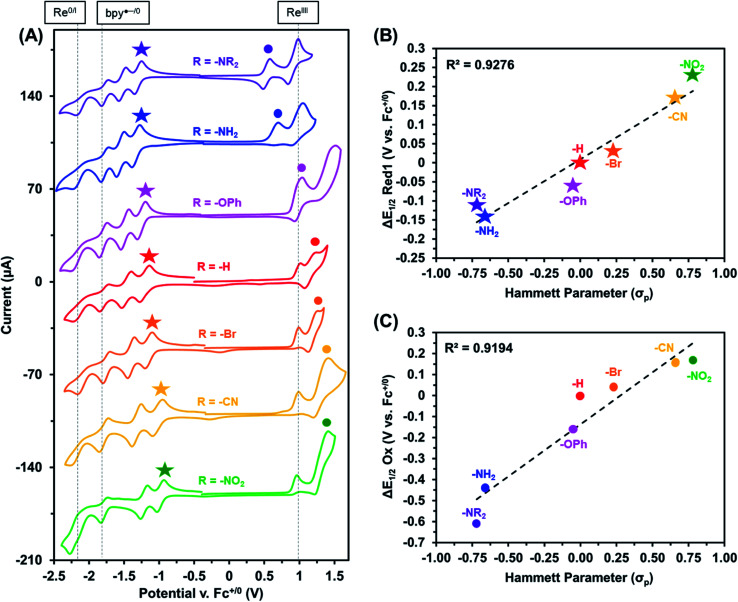
Cyclic voltammograms (A) of Re(bpy-C2-NPDI-R) dyads where R = –NR_2_ (purple), –NH_2_ (blue), –OPh (magenta), –H (red), –Br (orange), –CN (yellow), and –NO_2_ (green). All measurements were recorded at 100 mV s^−1^, under argon in CH_2_Cl_2_ with 0.1 M TBAPF_6_ supporting electrolyte (WE = glassy carbon, CE = Pt-wire, RE = Ag/AgCl, and Fc^+/0^ as internal reference standard). Correlation diagrams plot the observed shifts in *E*_1/2_ (relative to R = –H) for the first reduction (B) and oxidation (C) as a function of R-group Hammett parameter.^46^

The electrochemical properties of these Re(bpy-C2-NPDI-R) dyads were next evaluated using cyclic voltammetry (CV). CV analysis was first performed in CH_2_Cl_2_ (Fig. 2A and S65†), with all reported redox events being referenced to the Fc^+/0^ internal standard. Under an atmosphere of argon, all Re(bpy-C2-NPDI-R) dyads exhibited four reduction and two oxidation redox processes. The first two reversible reductions may be assigned to the NPDI˙^−/0^ and NPDI^2−/^˙^−^ redox couples while the third and fourth reductions correspond to the quasi-reversible bpy˙^−/0^ and the irreversible Re^0/I^ redox events, respectively.^11–18,45^ With respect to the oxidation events, the irreversible Re^II/I^ redox process remains consistently near *E*_p_ ≈ +1.0 V. The quasi-reversible NPDI-based oxidation event, on the other hand, underwent dramatic shifts in potential depending on the nature of the electronic substituent.

Based on the presented CV data (Fig. 2A, S64 and S65†), it was observed that the installation of EWGs (*i.e.* –Br, –CN, and –NO_2_) on NPDI caused both the redox events assigned to the lowest unoccupied molecular orbital (LUMO) and the highest occupied molecular orbital (HOMO) energy levels of NPDI to shift to more positive potentials. In all cases, the electronic bandgap was narrowed because the LUMO energy level was more significantly perturbed than the HOMO energy level. Conversely, the installation of EDGs (*i.e.* –OPh, –NH_2_, and –NR_2_) on NPDI caused the redox events associated with NPDI HOMO and LUMO energy levels to shift to more negative potentials. Once again, the electronic bandgap was decreased mainly due to the more substantial effects experienced by the HOMO energy level. The observed shifts in *E*_1/2_ for first NPDI-R based reduction and oxidation events (Fig. 2B and C), relative to –H, show good Hammett parameter correlation.^46^ Similar correlations were previously reported for electronically-substituted Re(4,4′-R-bpy) complexes by Kubiak *et al.*,^11^ where EWGs shifted redox events more positively and EDGs shifted redox events more negatively. In principle, combining these two relationships could assist with proper energy level matching when designing future supramolecular dyads based on the Re(bpy-C2-NPDI-R) architecture.

**Fig. 3:**
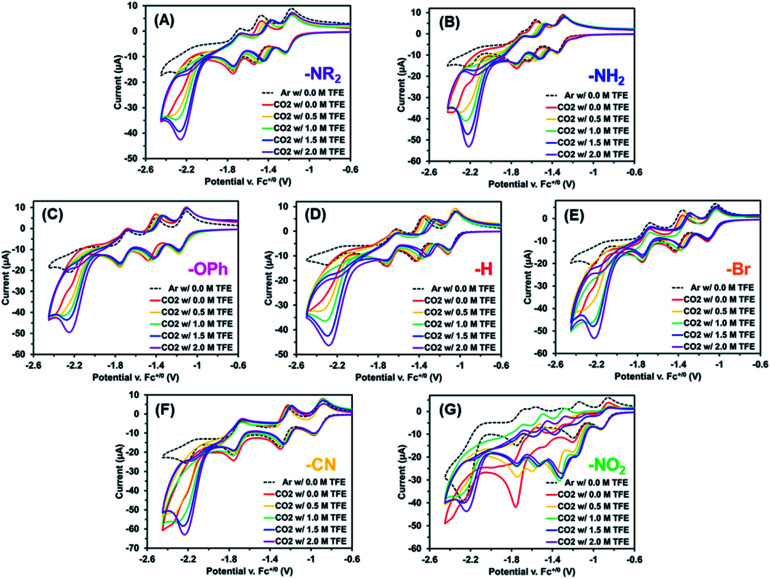
CV current enhancement plots as a function of 2,2,2-trifluoroethanol (TFE) concentration for Re(bpy-C2-NPDI-R) derivatives, where R = –NR_2_ (A), R = –NH_2_ (B), R = –OPh (C), R = –H (D), R = –Br (E), R = –CN (F), and R = –NO_2_ (G). TFE was incrementally added at 0 M (red), 0.5 M (yellow), 1.0 M (green), 1.5 M (blue), and 2 M (purple). All measurements were recorded at 100 mV s^−1^, under an atmosphere of CO_2_, in DMF with 0.1 M TBAPF_6_ supporting electrolyte (WE = glassy carbon, CE = Pt-wire, RE = Ag/AgCl, and Fc^+/0^ as internal reference standard). Note, the observed current enhancement at *E*_p_ = −1.35 V (*vs.* Fc^+/0^) for R = –NO_2_ (G) results from an *in situ* electrochemical conversion to R = –NH_2_.

Next, the electrochemistry of these Re(bpy-C2-NPDI-R) dyads were assessed in DMF, where only the reduction processes were measured due to solvent window effects.^59^ Under argon, all Re(bpy-C2-NPDI-R) dyads exhibited the same four previously assigned reduction events (*vide supra*), where solvent effects caused the potential of most redox events to shift positively by ∼50–100 mV, relative to CH_2_Cl_2_ (Fig. S66†).^60^ Upon measuring CVs at variable scan rates, each dyad displayed a diffusion-limited current response when fitted to the Randles–Sevcik equation (see ESI, eqn (i)†), with a calculated diffusion coefficient between *D* = 2.3–4.4 × 10^−6^ cm^2^ s^−1^ (Fig. S69–S74†). Upon subjecting these Re(bpy-C2-NPDI-R) dyads to an atmosphere of CO_2_ (in DMF), a moderate CV current enhancement was observed underneath the fourth redox couple (Fig. 3, red traces with *E*_cat/2_ ≈ −2.1 V). When a catalyst is supplied with the appropriate combination of substrates at a sufficient applied potential, a CV current enhancement is often observed as a result of initiating an electron-consuming catalytic process.^61,62^ Moreover, these CV current enhancements may be monitored as a function of catalyst or proton-source concentration to gather preliminary mechanistic data on the catalytic process (see ESI, eqn (ii)†).^63,64^

When the Re(bpy-C2-NPDI-R) concentration was varied, a linear CV current enhancement response was also observed for most dyads (Fig. S68†). Similarly, when proton-source 2,2,2-trifluoroethanol (TFE) was incrementally added to a CO_2_-saturated Re(bpy-C2-NPDI-R) dyad solution, it induced a CV current enhancement increase under the fourth reduction event (Fig. 3). We further note that incremental addition of TFE also caused the NPDI^2−/^˙^−^ redox couple to gradually shift to more positive potentials. This behavior has been modeled for similar rylene diimide materials as a 2H^+^/2e^−^ proton-coupled electron-transfer process,^65^ whereby the NPDI imide oxygens are protonated by the proton-source.^66,67^ While the collected CV data was not obtained under steady-state conditions (*i.e.* plateau current),^68^ modelling these measured CV current enhancements as CO_2_ reduction variables can qualitatively describe the effects of catalyst and proton-source as first-order and second-order rate-dependent variables, respectively. In other words, these data imply that one Re(bpy-C2-NPDI-R) dyad, with the assistance of two proton-source molecules, can enable electrocatalytic CO_2_ conversion.^45^ The obvious exception to these generalized electrochemical trends is the –NO_2_ dyad (Fig. 3G). An in-depth evaluation of the electrochemical behaviour of –NO_2_ under argon and CO_2_ is provided in the ESI (see Section VIII, Fig. S84–S86†). Thorough analysis of this data strongly suggests that –NO_2_ is converted *in situ* to –NH_2_ under reducing conditions; as such, it is difficult to establish meaningful trends for –NO_2_ (*vide infra*).

### CO_2_ electro-/photocatalysis

The electrocatalytic CO_2_ reduction abilities of these Re(bpy-C2-NPDI-R) dyads were evaluated using controlled potential electrolysis (CPE). All experiments were performed in DMF (with 2 M TFE) using our previously described two-compartment H-cell.^45,69^ At an applied potential (*E*_appl_) of −1.8 V (Fig. S75†), all Re(bpy-C2-NPDI-R) dyads (except –CN) achieved comparable turnover numbers of CO (TON_co_ = 21–25) and faradaic efficiencies (FE_co_ = 87–99%) after 6 hours of electrocatalysis. The –CN derivative, on the other hand, attained about half the performance (TON_co_ = 13) at a slightly lower FE_co_ (87%). This drop in performance is consistent with decreased efficacy of the electron-reservoir effect due to the increased electron-affinity of NPDI-CN (Fig. S77†). Unlike the electronically modified Re(4,4′-R-bpy) series reported by Kubiak *et al.*, where electrocatalytic CO_2_-to-CO conversion efficiency was highly dependent on the nature of the R-group,^11^ all Re(bpy-C2-NPDI-R) dyads achieved high FE_co_ at an overpotential that is ∼300 mV lower than the measured *E*_cat/2_ ≈ −2.1 V (Fig. S67†). It should be noted, however, that when an *E*_appl_ = −1.7 V was used for CPE (Fig. S76†), electrocatalytic CO_2_ reduction was essentially shut-off for all Re(bpy-C2-NPDI-R) dyads (TON_co_ ≤ 6). This could indicate that altering the electronic properties of the electron-reservoir may not be the most effective strategy towards further lowering the overpotentials required to enable electrocatalytic CO_2_ reduction.

In our previous report, we noticed that the use of blue light during CPE experiments significantly increased the rates of CO production for –H.^45^ To determine the origin of this TON_co_ enhancement,^28,70,71^ we next turned our attention towards the photocatalytic CO_2_ reduction capabilities of these Re(bpy-C2-NPDI-R) dyads. Following established literature protocols,^31,34–37^ each dyad (30 μM) was dissolved in a (5 : 1) DMF: triethanolamine (TEOA) mixture containing sacrificial reducing agent 1,3-dimethyl-2-phenyl-2,3-dihydro-1*H*-benzo[*d*]imidazole (BIH; 3 mM). The glass vials were sealed with rubber septa, sparged with CO_2_, and then irradiated with blue light (*λ* = 470 ± 30 nm; 4 mW cm^−2^) for 24 hours (Table 1).

**Table 1:** Optimized photocatalytic CO_2_ reduction of Re(bpy-C2-NPDI-R)

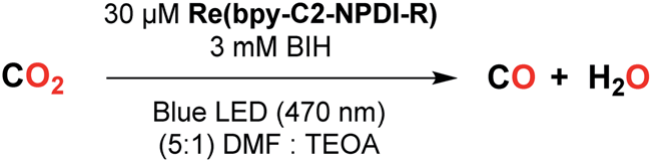			
R =	TON_CO_^a^	TON_H2_^a^	CO : H_2_^b^
–H	57 ± 1	1.8 ± 1	97 : 3
–CN	<1	<1	—
–NO_2_^c^	137 ± 15	3 ± 0.6	98 : 2
–Br	61 ± 5	2.5 ± 1	96 : 4
–NR_2_	86 ± 8	1.4 ± 1	98 : 2
–OPh	59 ± 6	3.6 ± 2	95 : 5
–NH_2_	234 ± 13	2.0 ± 0.1	>99 : 1

a Calculated based on bulk catalyst concentration from quadruplicate trials.

b No other gaseous (*i.e.* CH_4_) or liquid (*i.e.* HCOO–) products were detected.

c Re(bpy-C2-NPDI-NO_2_) is *in situ* converted to Re(bpy-C2-NPDI-NH_2_).

Over the 24 h testing period, the Re(bpy-C2-NPDI-R) dyads all showed good activity for ∼9 hours, after which CO production would level-off for the remainder of the experiment (Fig. S78†). The benchmark dyad, –H, achieved a TON_co_ of 57 ± 1 with a selectivity for CO of 97%. The –Br (TON_co_ = 61 ± 5) and –OPh (TON_co_ = 59 ± 6) dyads achieved the same performance with roughly the same CO selectivity (≥95%). The –CN derivative was completely inactive for CO_2_ photocatalysis under these conditions. Interestingly, despite being the most EWG, the –NO_2_ dyad achieved the second best TON_co_ (134 ± 15) with a very high CO selectivity of 98%. When EDGs were functionalized on NPDI, the CO_2_-to-CO production and selectivity was improved to (TON_co_ = 86 ± 8) for –NR_2_ and (TON_co_ = 234 ± 13) for –NH_2_. We note that the photocatalytic performance of –NH_2_ was not greatly improved over the 24 h testing period by replenishing both BIH and CO_2_ in 6 h intervals (TON_co_ = 294; Fig. S79†), suggesting that depletion of substrate was not the limiting factor for TON_co_.

To confirm the importance of each component in the photocatalytic CO_2_ reduction setup, various control experiments were conducted. As expected, the omission of Re(bpy-C2-NPDI-R) dyads or CO_2_ from the setup stopped the production of CO. When sacrificial reducing agent BIH was excluded (Table S2†), the TON_co_ was decreased by at least two-fold for all dyads. This result implies that while TEOA alone can simultaneously act as the proton-source and the sacrificial electron-donor,^20^ BIH is more efficient at reductively quenching the photoexcited dyads. When TEOA was replaced by TFE (Table S3†), the production of CO for all dyads was decreased almost four-fold, except for –NH_2_ (TON_co_ = 143). Overall, this result points towards the utility of TEOA as a sacrificial electron-donor, as well as the importance of forming Re(bpy)-adducts during CO_2_ reduction, as seen in previous literature examples.^5,26–31^ When the irradiation source was switched to a green light LED array (*λ* = 525 ± 32 nm; 1.9 mW cm^−2^), the measured TON_co_ decreased at least five-fold for all Re(bpy-C2-NPDI-R) dyads (Table S4†). This significant drop in performance could be the result of either inefficient photoexcitation pathways in the dyad^40–44^ and/or the elimination of a photo-assisted CO cleavage process.^70,71^ Lastly, the importance of tethering the NPDI-moiety to the Re(bpy)-moiety was demonstrated by combining N_3_-C2-NPDI-R with Re(bpy), where it was observed that all samples obtained the same performance as Re(bpy) alone (Table S5†). We further showed that all N_3_-C2-NPDI-R precursors were essentially inactive for CO_2_ conversion under the optimized photocatalysis conditions (TON_co_ < 3; Table S6†).

While these control experiments clearly highlight the necessity of each component in the photocatalytic CO_2_ reduction process, it does not fully account for the performance differences of each Re(bpy-C2-NPDI-R) dyad. Therefore, based on the photocatalytic CO_2_ reduction results, the Re(bpy-C2-NPDI-R) dyads may be grouped together in three categories: (i) standard catalysts (weak EWGs/EDGs = –H, –Br, and –OPh), (ii) inactive catalysts (strong EWGs = –CN), and (iii) top-performing catalysts (strong EDGs = –NR_2_ and –NH_2_). Note, the –NO_2_ dyad can also be classified as a top-performing catalyst because –NO_2_ undergoes an *in situ* transition to –NH_2_ under photocatalytic CO_2_ reduction conditions (see ESI Section VIII for more details†).

### Mechanistic investigation

During electro-/photocatalytic CO_2_ reduction testing of these Re(bpy-C2-NPDI-R) dyads, a series of dramatic colour changes were observed. Prior to light irradiation, the absorption profile of all Re(bpy-C2-NPDI-R) dyads were unchanged by the addition of both TEOA and BIH, confirming that neither reagent reduces Re(bpy-C2-NPDI-R) immediately. After sparging with CO_2_, the samples were then subjected to blue light and the progression of colour changes was monitored periodically by UV-vis-nIR spectroscopy (Fig. S80†). Each Re(bpy-C2-NPDI-R) dyad underwent a transition from their initial colour to either a green (–H, –Br, and –OPh), a dark blue (–CN), or a beige (–NO_2_, –NR_2_, and –NH_2_) colour that was found to persist throughout the remainder of catalysis. Photoluminescence spectroscopy revealed that the photoexcited state of all Re(bpy-C2-NPDI-R) dyads can be reductively quenched by both TEOA and BIH (Fig. S81†). Thus, to gain further insight into the photoelectrochemical processes that were occurring during CO_2_ reduction catalysis, UV-vis-nIR and FTIR spectroelectrochemistry (SEC) experimentation was conducted. UV-vis-nIR and FTIR SEC data was collected by monitoring an air-free Re(bpy-C2-NPDI-R) dyad solution that was held at a constant *E*_appl_ (where Red1, Red2, and Red3 correspond to the NPDI˙^−/0^, NPDI^2−/^˙^−^, and bpy˙^−/0^ reductions, respectively).

**Fig. 4:**
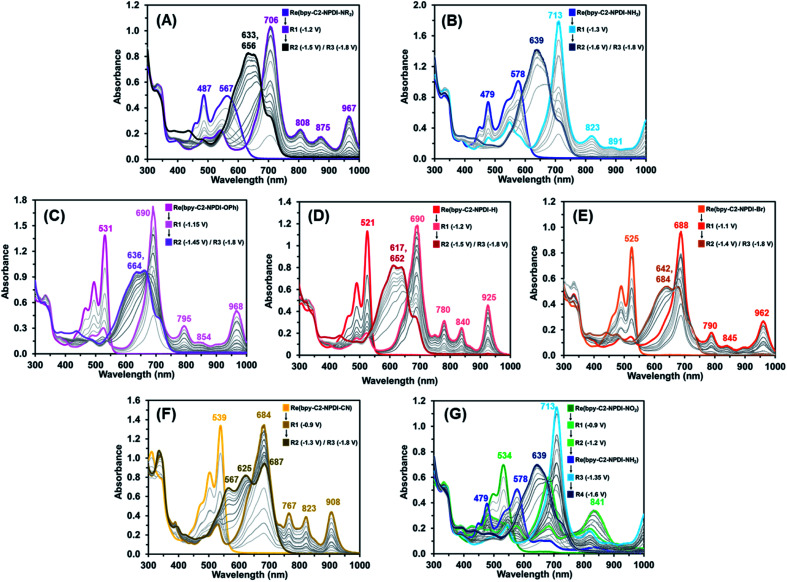
UV-vis-nIR SEC absorbance spectra of Re(bpy-C2-NPDI-R) derivatives, where R = –NR_2_ (A), R = –NH_2_ (B), R = –OPh (C), R = –H (D), R = -Br (E), R = –CN (F), and R = –NO_2_ (G). All experiments were performed in DMF with 0.1 M TBAPF_6_ supporting electrolyte (WE = Pt-mesh, CE = Pt-wire, pseudo-RE = Ag-wire).

The UV-vis-nIR SEC data of each Re(bpy-C2-NPDI-R) dyad (Fig. 4) correlates very well with what was observed when we periodically monitored our photocatalytic experiments. When using an *E*_appl_ = Red1, the *λ*_max_ of all Re(bpy-C2-NPDI-R) dyads was bathochromically shifted, the relative molar absorptivity of this *λ*_max_ was stronger, and some new vibrational fine-structure was also observed between 800–1000 nm. While the nature of installed R-group influences the position of the *λ*_max_ and the absorption fine-structure, all these absorption features are consistent with selective formation of NPDI˙^−^.^43,44^ When the *E*_appl_ is switched to Red2, the spectral features of NPDI˙^−^ are rapidly depleted and replaced by shifted broad-band absorption peak(s). These spectral features are commonly associated with NPDI^2−^,^67^ where the vibronic structure of the NPDI^2−^ absorption profile is once again influenced by the nature of installed R-group. With respect to –H (Fig. 4D), the incorporation of HOMO-modifying EDGs (*i.e.* –NR_2_, –NH_2_, and –OPh) resulted in minimal changes to the NPDI^2−^ absorption profile shape (Fig. 4A–C). Conversely, when LUMO-modifying EWGs (*i.e.* –Br and –CN) are used, the vibronic structure of NPDI^2−^ is significantly different (Fig. 4E and F). When the *E*_appl_ was changed to Red3, no other significant spectral changes were observed. This result is unsurprising given the differences in molar absorptivity of the NPDI and Re(bpy)-moieties.^57,72^ Except for –NO_2_ (Fig. 4G), all Re(bpy-C2-NPDI-R) dyads displayed very similar spectral transitions. In-depth analysis of the UV-vis-nIR SEC data for –NO_2_ (Fig. S86†) shows that –NO_2_ is *in situ* converted to –NH_2_ under the conditions necessary for CO_2_ reduction catalysis. These results help confirm why –NO_2_ served as an efficient CO_2_ reduction photocatalyst despite having a stronger EWG than the totally inactive –CN dyad derivative.

Moving onto the FTIR SEC data, the behavior of all Re(bpy-C2-NPDI-R) dyads were nearly identical (Fig. S58–S63†). While no spectral changes were detected at *E*_appl_ = Red1 and Red2, significant shifts in *v*_co_ were observed at *E*_appl_ = Red3. At Red3, the Re(bpy)-moiety of these dyads is formally reduced by one-electron [Re^I^(bpy˙^−^-C2-NPDI^2−^-R)]. The added electron density at the Re(bpy)- moiety results in a lowering of *v*_co_ from 1895, 1915, and 2019 cm^−1^ to roughly 1865, 1885, and 1995 cm^−1^, respectively. Over time, an equilibration process occurs whereby electron-density is shifted from bpy˙^−^ to Re, and leads to Re–Cl dissociation [Re^0^(bpy-C2-NPDI^2−^-R)].^58^ This crucial process generates a 5-coordinate Re metal-center and can be characterized by a Δ*v*_co_ to 1843, 1862, and 1978 cm^−1^. Although this Cl-dissociation process was more readily observed for dyads bearing EWGs (–CN, –NO_2_, and –Br) than it was for dyads bearing EDGs (–NR_2_, –NH_2_, and –OPh), it was still detected to some degree for all Re(bpy-C2-NPDI-R) catalysts.

**Fig. 5:**
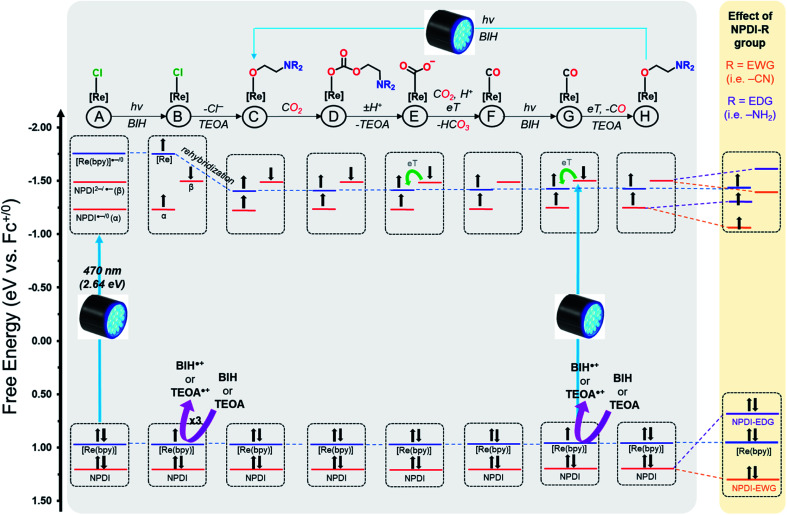
Molecular orbital description for the proposed photocatalytic CO_2_ reduction mechanism of Re(bpy-C2-NPDI-R), where Re(bpy) orbitals are shown in blue and NPDI-R orbitals are shown in red. The influence of electron withdrawing groups (EWGs, orange) and electron donating groups (EDGs, purple) on NPDI-R molecular orbitals is highlighted (right). Both BIH and TEOA are depicted as sacrificial electron donors that reductively quench the photoexcited electrons.

The similarities between our SEC data and the results reported for other Re(bpy) and Ru^II^–Re^I^ catalysts suggests that our Re(bpy-C2-NPDI-R) systems likely operate *via* similar photocatalytic CO_2_ reduction mechanisms.^5,25–31^ It should be noted, however, that the photocatalytic CO_2_ reduction mechanism for Re(bpy) is still heavily debated.^18–31,73^ As such, the goal of our proposed mechanism was not to precisely determine the exact identity, electron-spin configuration (singlet *vs.* triplet), and/or the rate dynamics of eT for all photocatalytic CO_2_ reduction intermediates. Instead, we focus on developing a molecular orbital (MO) description of these Re(bpy-C2-NPDI-R) dyads that helps account for the observed differences in photocatalytic CO_2_ reduction performance (Fig. 5). The presented mechanism was modeled after the benchmark dyad, –H.

By CV, it was elucidated for all Re(bpy-C2-NPDI-R) dyads (except –NH_2_ and –NR_2_) that the HOMO is Re(bpy)-based and the LUMO is NPDI-based (Fig. 5A). Consequently, the first two photoreductions most likely result from a Re–π* intersystem crossing (ISC) process; however, a direct NPDI-based π–π* transition may also be possible.^43,44^ The third photoreduction likely occurs either *via* direct Re(bpy) ^3^MLCT^19^ or eT from a photoexcited state of NPDI^2−^.^43,44^ Following the formal three electron reduction of the Re(bpy-C2-NPDI-R) dyads (Fig. 5B), the next step is Re–Cl dissociation which generates a 5-coordinate Re metal-center whose axial position subsequently forms an adduct with TEOA.^20,74^ Another consequence of the chloro-dissociation step is that the Re metal-center undergoes a rehybridization process that lowers the overall energy of the Re(bpy)-based MOs.^45,75^ Following the formation of the TEOA-Re(bpy) adduct, it is possible for CO_2_ insertion to occur without direct eT from the catalyst center (Fig. 5D).^20,27–30^ Protonation of the resulting carbonate intermediate induces a reorganization process that releases TEOA and forms a Re-CO_2_˙^−^ species (Fig. 5E).^22^ Due to the presence of ^13^CO and H^13^CO_3_^−^ in the ^13^C{^1^H} NMR spectrum after blue light irradiation (Fi. S83†), it is postulated that these Re(bpy-C2-NPDI-R) dyads operate *via* a BIH-mediated disproportionation reaction between Re–CO_2_˙^−^ and another equivalent of CO_2_ that liberates HCO_3_^−^,^25^ rather than a proton-coupled electron transfer process from NPDI^2−^ to the Re(bpy)-moiety that liberates OH^−^.^18^ From there, reductive quenching of a photoexcited electron restores NPDI^2−^ (Fig. 5G) and the ensuing transfer of this electron to the Re(bpy)-moiety produces CO, as well as opens a coordination site for TEOA (Fig. 5H).^21^ The photocatalytic cycle is completed by the photoexcitation and reductive quenching of the electron to regenerate NPDI^2−^.

Based on photocatalytic CO_2_ reduction performance, the catalysts were loosely grouped into three categories: (i) standard catalysts (–H, –Br, and –OPh), (ii) inactive catalysts (–CN), and (iii) top-performing catalysts (–NR_2_ and –NH_2_). Looking at the proposed photocatalytic CO_2_ reduction mechanism, it is also possible to map out the effects of EWGs and EDGs on the provided MO description of these Re(bpy-C2-NPDI-R) dyads (Fig. 5, highlighted in yellow). In the case of –Br and –OPh, the overall influence of these R-group does not appear to change the eT dynamics of the Re(bpy-C2-NPDI-R) dyad (with respect to –H). The HOMO–LUMO transition (Re–π*) is identical and the relative shift(s) of the NPDI^2−^ energy level does not significantly alter the electronic driving-force of eT between NPDI^2−^ and Re(bpy). In the case of –CN, the fixation of that EWG on NPDI served to lower the energy of the NPDI-based HOMO and LUMOs. While the net result of this transformation retains the original HOMO–LUMO transition (Re–π*), it appears to lower the energy level of NPDI^2−^ enough to effectively prevent eT from NPDI^2−^ to the Re(bpy)-moiety, thus shutting down catalysis. Conversely, the installation of strong EDGs (–NR_2_ and –NH_2_) causes the energy levels of the NPDI-based HOMO and LUMOs to increase. The overall result of this transformation not only changes the HOMO–LUMO transition to an exclusively NPDI-based process (π–π*), but it also increases the driving-force for eT from NPDI^2−^ to Re(bpy). The sum of these two effects together lead to improved dyad eT dynamics, thereby enhancing photocatalytic CO_2_ reduction (with respect to –H).

It should be noted that another feasible explanation for the improved performance of the amino-functionalized dyads, in particular –NH_2_, is the possibility of second-sphere H-bonding effects.^15,18^ Previously we calculated the optimized geometries of various intermediates during CO_2_ catalysis for the –H dyad.^45^ It was shown in this study that, although the NPDI was initially folded over the Re(bpy)-moiety, reduction of the –H dyad caused the two moieties to extend away from one another (likely due to coulombic repulsion effects). By analogy, the amino-functionalized NPDI-R bay position would most likely also be extended away from the Re(bpy)-moiety during CO_2_ photocatalysis. While in this case the distance between the catalyst center and the amino-groups of –NR_2_ and –NH_2_ make it unlikely that second-sphere H-bonding effects are involved in CO_2_ photocatalysis, they can't be conclusively ruled out at this time. If nothing else, the synthetic versatility of the NPDI chromophore means that future iterations of the Re(bpy-C2-NPDI-R) motif could incorporate proximal second-sphere H-bonding groups as a means to further improve CO_2_ conversion performance.

## Conclusions

In conclusion, we present the synthesis and full characterization of six new Re(bpy-C2-NPDI-R) supramolecular dyad materials (where R = –Br, –CN, –NO_2_, –OPh, –NH_2_, or –NR_2_). The installation of R-groups on NPDI altered the optoelectronic properties of these dyads, as well as impacted the photocatalytic CO_2_ reduction performance. Relative to the benchmark Re(bpy-C2-NPDI-H) dyad (TON_co_ = 57), the incorporation of EDGs (*i.e.* –NH_2_) led to an over four-fold improvement in photocatalytic CO_2_ reduction performance (TON_co_ = 234) while strong EWGs (*i.e.* –CN) resulted in complete deactivation of the dyads. Despite being the most electron-withdrawing, the –NO_2_ functionalized NPDI was among the top performing CO_2_ reduction photocatalysts (TON_co_ = 137), making it an outlier to the proposed trend. Through CV and UV-vis-nIR SEC experimentation, it was elucidated that –NO_2_ undergoes an *in situ* conversion to –NH_2_, thereby forming a different dyad that is responsible for catalysis. A photocatalytic CO_2_ reduction mechanism is proposed for these dyads, where EDGs served to accelerate CO_2_ reduction rates by simultaneously changing the HOMO–LUMO excitation pathway and by increasing the electronic driving-force of intramolecular electron transfer from NPDI^2−^ to Re(bpy). Conversely, EWGs shifted the LUMO energy levels of NPDI to the point where photocatalysis is shut down because there is no electronic driving force for eT between NPDI^2−^ and Re(bpy). This study clearly highlights the importance of evaluating structure–property relationships to develop and optimize the future design of new supramolecular dyad photocatalysts.

## Author contributions

JDBK performed all experimental work and data analysis and prepared the manuscript. GCW directed the project and provided resources. WEP co-directed the project and provided resources.

## Conflicts of interest

There are no conflicts to declare.

## Supplementary Material

SC-013-D1SC05465A-s001
