# Supplementary material for: Promoting photocatalytic CO2 reduction through facile electronic modification of N-annulated perylene diimide rhenium bipyridine dyads
Source: Chem Sci. 2021 Dec 28;13(4):1049–59. doi: 10.1039/d1sc05465a (PMC8790914; doi:10.1039/d1sc05465a)
Supplement: SC-013-D1SC05465A-s001 [file SC-013-D1SC05465A-s001.pdf]

Electronic Supplementary Material (ESI) for Chemical Science.
This journal is © The Royal Society of Chemistry 2021

Promoting Photocatalytic CO_2 Reduction Through Facile Electronic Modification of N-Annulated Perylene Diimide Rhenium Bipyridine Dyads

(Supporting Information)

Josh D. B. Koenig[a*], Warren E. Piers[a], and Gregory C. Welch[a*]

[a] Department of Chemistry, University of Calgary, 2500 University Drive N.W., Calgary, Alberta, T2N 1N4, Canada.

* Corresponding Authors:
Email: joshua.koenig1@ucalgary.ca & gregory.welch@ucalgary.ca
Phone Number: 1-403-210-7603

Keywords: CO_2 reduction; electrochemical catalysis; molecular catalysis; rhenium; bipyridine; Re(bpy); perylene diimide; PDI

TABLE OF CONTENTS

1. **Materials and Methods** **S2 – S4**
2. **Synthetic/Experimental Procedures** **S5 – S13**
3. **^{1}H & ^{13}C NMR Spectroscopies** **S14 – S31**
4. **MALDI-TOF MS & CHN Elemental Analysis** **S32 – S40**
5. **UV-Vis-nIR / FTIR Spectroscopies & Spectroelectrochemistry** **S41 – S49**
6. **Cyclic Voltammetry** **S50 – S60**
7. **Electro-/Photocatalytic CO_2 Reduction** **S61 – S70**
8. **Evaluation of Re(bpy-C2-NPDI-NO_2)** **S71 – S73**
9. **References** **S74**

1. Materials and Methods

Materials: All air-free reactions were carried out under an atmosphere of N_2 using standard glove box or high vacuum line (Schlenk) techniques unless otherwise stated. All reactants, reagents, and catalysts for synthesis were purchased from Millipore-Sigma or Fisher Scientific and used without further purification.

Nuclear Magnetic Resonance (NMR): All ^{1}H, ^{1}H-^{1}H COSY, and ^{13}C NMR spectroscopy experiments were recorded using a Bruker Avance III 600 MHz spectrometer. All experiments were performed in chloroform-d ($CDCl_3$), tetrachloroethane-d_2 (TCE-d_2), or N,N-dimethylformamide-d_7 (DMF-d_7). Chemical shifts (referenced to residual solvent) were reported in parts per million (ppm).

High-resolution MALDI-TOF (HR MALDI-TOF): High-resolution MALDI-TOF mass spectrometry measurements were performed courtesy of Johnson Li in the Chemical Instrumentation Facility at the University of Calgary (UofC). Sample solution (~ 1 µg/mL in CH_2Cl_2) was mixed with matrix trans-2-[3-(4-tert-Butylphenyl)-2-methyl-2-propenylidene] malononitrile (DCTB) solution (~5 mg/ml in methanol). All spectra were acquired using a Bruker Autoflex III Smartbeam MALDI-TOF, set to the positive reflective mode (Na:YAG 355 nm laser settings: laser offset = 62-69; laser frequency = 200 Hz; and number of shots = 300). The target used was Bruker MTP 384 ground steel plate target.

CHN Elemental Analysis: Elemental analyses were performed by Johnson Li in the Chemical Instrumentation Facility at the UofC. A Perkin Elmer 2400 Series II CHN Elemental Analyzer was used to obtain CHN data, using ~1.5 mg of sample (with particle sizes ranging between 0.2 and 0.5 mm in diameter).

Cyclic Voltammetry (CV): All electrochemical measurements were performed using a CH Instruments Inc. Model 620E Potentiostat. A standard 3-electrode setup was utilized, consisting of a freshly polished glassy carbon disk WE, Pt-wire CE, Ag/AgCl pseudo-RE, and ferrocene ($Fc^{+/0}$) as an internal standard. Unless otherwise specified, all CV experiments were performed in anhydrous CH_2Cl_2 or N,N-dimethylformamide (DMF) at a scan rate of 100 mV/s with 0.1 M tetrabutylammonium hexafluorophosphate ($TBAPF_6$) supporting electrolyte. All electrochemical solutions were sparged with dry gas (either argon or CO_2) for at least 5 minutes prior to measurements. The impact of proton source concentration on catalytic current enhancement was tested using 2,2,2-trifluoroethanol (TFE; pKa = 24.0).[1] An initial measurements was run under argon and then the solution was sparged with CO_2. To the CO_2-saturated sample (0.23 M in DMF), TFE was added incrementally until a maximal current enhancement was achieved.

UV-Visible Spectroscopy (UV-Vis): Optical absorption measurements were performed using Agilent Technologies Cary 60 UV-Vis spectrometer at ambient conditions. All solution UV-Vis spectra were measured with 2 or 10 mm quartz cuvettes, using $CHCl_3$, N,N-dimethylformamide (DMF), or tetrahydrofuran (THF) as solvent. Stock solutions (~1 mg/mL) of each compound were prepared, serially diluted to concentrations between 10^{-5} - 10^{-6} M, and then used to construct calibration curves for determining molar absorptivity.

Fourier Transform Infrared Spectroscopy (FTIR): All IR absorption measurements were performed using an Agilent Technologies Cary 630 FTIR spectrometer at room temperature. FTIR spectra were measured from $CHCl_3$ or DMF solutions of the analyte, using either the dial-path module (pathlength = 100 microns) or the transmission module.

Photoluminescence Spectroscopy (PL): All PL emission measurements were performed using an Agilent Technologies Cary Eclipse fluorescence spectrometer at room temperature. Samples were prepared at a concentration of 30 μM, using 3 mL of either neat DMF or a (5:1) mixture of DMF:triethanolamine (TEOA). The excitation wavelength (λ_{ex}) was set to 470 nm. Spectrometer slit widths were adjusted depending on the emission intensity of each analyte, but held constant for all samples related to that particular analyte to allow for normalization of data.

Spectroelectrochemistry (SEC): All UV-vis SEC and FTIR SEC experiments were conducted using a LabOmak IR-SEC cell fitted with CaF_2 windows (pathlength = 0.2 mm), a Pt-mesh working electrode (WE), a Pt-wire counter electrode (CE), an Ag-wire pseudo-reference electrode (RE), and $Fc^{+/0}$ as an internal reference standard. The cell was filled with DMF solutions (~0.1 mL) containing 0.5 mM analyte and 0.1 M $TBAPF_6$. Solutions were sparged with argon in a scintillation vial for 15 minutes prior to use. Blank DMF solutions containing 0.1 M $TBAPF_6$ were used to baseline correct spectra at each voltage step.

Controlled Potential Electrolysis (CPE): CPE experiments were performed with a customized two-compartment H-shaped cell, using a CH Instruments Inc. Model 620E Potentiostat. A glassy carbon plate (20 mm x 8 mm x 2 mm) was used as the WE, along with a Pt-mesh CE, and a non-aqueous Ag/AgCl pseudo-RE. For all experiments using the two-compartment H-shaped cell, two solutions were prepared: an active solution (0.5 mM catalyst + 0.1 M $TBAPF_6$ + TFE), and a blank solution (0.1 M $TBAPF_6$ + TFE). The glassy carbon plate WE and Ag/AgCl pseudo-RE were immersed into the active solution in the cathodic compartment, while the Pt-mesh CE was immersed into the blank solution in the anodic compartment. Using the chronoamperometry setting, the potentiostat applied constant voltage for the duration of testing.

Photochemistry: CO_2 photocatalysis experiments were performed using a 10.5 mL glass pressure vial that was sealed with a rubber septum. The catalyst was dissolved in the specified solvent mixture (5:1, DMF:TEOA/TFE). Samples were sparged with CO_2 for 20 minutes prior to irradiation with a Thor Labs Blue LED ($\lambda = 470 \pm 30$ nm; 4.0 mW cm^{-2}). Headspace aliquots (50 μL) were removed after 24 hours of light irradiation to analyze gaseous products.

Gas chromatography (GC): GC headspace analysis (50 μL aliquots) was performed using an Agilent Technologies 7890B GC, equipped with a PoraPlot Q and PLOT molecular sieve (5 Å) column (oven temperature 60 – 120 °C) in series and a VICI pulsed discharge Helium ionization detector. The flow rate of the carrier gas (helium) was approximately 5 mL min^{-1}. The system was calibrated using a customized gas mixture, containing known concentrations of: H_2, CO, CH_4, ethylene, and ethane.

Data Handling/Calculations:

i) Randles Sevcik Equation (i_p scan rate dependency for diffusion-controlled processes) [2]

$$i_p = 0.4463\, nFAc_{cat}\sqrt{\frac{nFvD}{RT}}$$

Where: i_p = peak current of redox wave, n = # of electrons transferred, F = Faradaic constant, A = geometric area of the working electrode, c_{cat} = catalyst concentration, v = scan rate, D = diffusion coefficient, R = universal gas constant, and T = temperature.

ii) Determination of catalyst and substrate rate-dependences [3]

$$i_{cat} = nFA[cat]\sqrt{Dk_{sub}[sub]^x}$$

Where: i_{cat} = peak current of redox wave under catalytic conditions, n = # of electrons transferred, F = Faradaic constant, A = geometric area of the working electrode, [cat] = catalyst concentration, D = diffusion coefficient, k_{sub} = rate constant of substrate, [sub] = concentration of substrate (either TFE and/or CO_2), x = rate-dependence of substrate

iii) Determination of k_{obs} using the i_{cat} / i_p analysis [4]

$$\frac{i_{cat}}{i_p} = 2.2406\sqrt{\frac{RT}{nFv}\, n' k_{obs}}$$

Where: i_{cat} = peak current of redox wave under catalytic conditions, i_p = peak current of redox wave, R = universal gas constant, T = temperature, n = # of electrons transferred, F = Faradaic constant, v = scan rate, n' = # of catalyst molecules required per turnover (n' = 1), and k_{obs} = observed catalytic rate constant.

2. Synthetic/Experimental Procedures

Bromo[N-annulated perylene diimide] (HNPDI-Br)
Starting material, N-annulated perylene diimide (HNPDI), was synthesized using known literature procedure.[7]

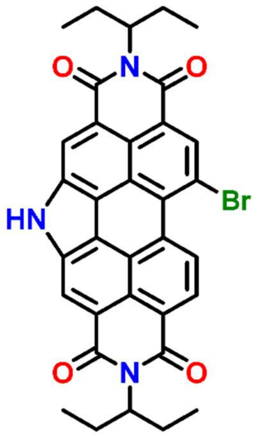

HNPDI (2.17 g, 4.00 mmol, 1 eq.) was combined with elemental Br_2 (10.3 mL, 200 mmol, 50 eq.) in a charged 250 mL round-bottom flask (rbf). The rbf was sealed with a rubber septum and left to stir at room temperature for 2 hours. The reaction was very slowly quenched using a saturated $Na_2S_2O_5$ aqueous solution and then stirred for 15 minutes. The resulting red precipitate was collected by vacuum filtration and copiously rinsed with H_2O and then MeOH until the rinsate was colorless (2.48 g, 3.99 mmol, 99% yield). No additional purification was required, as the collected product matched known MALDI data.[8]

Cyano[N-annulated perylene diimide] (HNPDI-CN)
Rosenmund-von Braun reaction conditions were modified from literature example.[5]

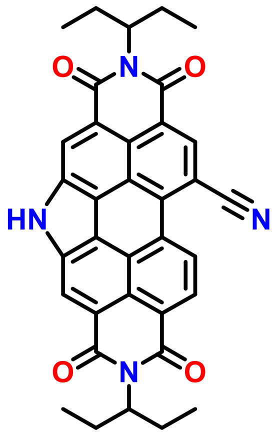

HNPDI-Br (623 mg, 1.01 mmol, 1 eq.) and CuCN (1.79 g, 20.0 mmol, 20 eq.) were added to a 20 mL glass pressure vial, dissolved in N,N-dimethylformamide (15 mL), and then the vial was sealed. After sparging with N_2 for 15 minutes, the reaction vessel was placed into a 160 °C bead bath and left to stir for 24 hours, monitoring reaction progress by thin-layer chromatography (TLC). Following reaction completion, the mixture was cooled to room temperature, poured into MeOH (150 mL), and then dilute $HCl_{(aq)}$ solution was added until dark red precipitate formed. The dark red precipitate was collected by vacuum filtration, rinsing with copious MeOH. After drying, the crude solid was subsequently dissolved in THF and filtered through a short silica-gel plug (eluting with THF). After solvent was removed by rotary evaporation, the resulting solid was collected by vacuum filtration (550 mg, 0.976 mmol, 98% yield).

<u>^{1}H NMR</u> (400 MHz, $CDCl_3$) δ 10.08 (d, J = 8.4 Hz, 1H), 9.98 (s, 1H), 9.25 (s, 1H), 9.14 (s, 1H), 9.02 (d, J = 8.4 Hz, 1H), 5.27 – 5.17 (m, 2H), 2.35 (tt, J = 16.4, 8.2 Hz, 4H), 2.04 (dt, J = 14.5, 6.6 Hz, 4H), 1.06 – 0.80 (m, 12H).

<u>^{13}C NMR</u> (100 MHz, $CDCl_3$) *due to poor solubility of the material, no data was collected*

<u>HRMS</u> ($[M-H]^+$) calculated for M = $C_{35}H_{28}N_4O_4$: 567.2027; detected $[M-H]^+$: 567.2051

Cyano[azido-ethyl-N-annulated perylene diimide] (N_3-C2-NPDI-CN)
Alkylation and azide functionalization procedures were modified from known literature.[6]

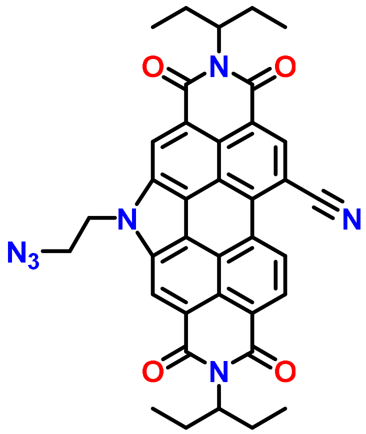

HNPDI-CN (284 mg, 0.499 mmol, 1 eq.) and K_2CO_3 (136 mg, 1.00 mmol, 2 eq.) were combined in a 20 mL glass pressure vial, dissolved in DMF (10 mL), and then the vial was sealed. After sparging with N_2 for 15 minutes, 1,2-dibromoethane (0.22 mL, 2.5 mmol, 5 eq.) was added to the solution. The reaction mixture was heated at 60 °C and left to stir for 18 hours. Next, reaction mixture was poured through a short Celite plug (eluting with CH_2Cl_2) and solvent was removed by rotary evaporation. The crude solid was re-dissolved in CH_2Cl_2, adhered to silica, and then loaded on top of a short silica-gel plug. The desired alkylated material was eluted using a (6:1) hexanes:acetone mixture. Following the removal of solvent by rotary evaporation, NaN_3 (63 mg, 0.95 mmol, 1.9 eq.) was added to the rbf containing the isolated material. The reactants were dissolved in DMF (30 mL) and left to stir at room temperature for 18 hours. After confirming reaction completion by TLC, the reaction mixture was poured into MeOH (100 mL) and H_2O (15 mL) was added to induce precipitation. The precipitated cherry red solid was collected by vacuum filtration and rinsed with a (19:1) MeOH:H_2O mixture (208 mg, 0.326 mmol, 65% yield over 2-steps).

<u>^{1}H NMR</u> (600 MHz, $CDCl_3$) δ 9.77 (d, J = 8.2 Hz, 1H), 9.19 (s, 1H), 9.07 (s, 1H), 8.98 (s, 1H), 8.85 (d, J = 8.0 Hz, 1H), 5.23 – 5.18 (m, 2H), 5.10 (dd, J = 7.3, 3.7 Hz, 2H), 4.18 – 4.11 (m, 2H), 2.39 – 2.32 (m, 4H), 2.09 – 2.01 (m, 4H), 1.00 (t, J = 7.4 Hz, 12H).

<u>^{13}C NMR</u> (151 MHz, $CDCl_3$) δ 165.65, 164.78, 135.54, 134.85, 133.55, 132.67, 130.55, 128.37, 128.19, 127.42, 126.42, 124.50, 123.83, 122.22, 121.88, 120.75, 119.20, 119.08, 118.69, 118.52, 106.85, 58.34, 58.17, 51.85, 46.16, 25.20, 25.11, 11.50, 11.45. *expected = 33 C; observed = 29 C; some quaternary carbons centers were not detected*

<u>HRMS</u> ($[M-H]^+$) calculated for M = $C_{37}H_{31}N_7O_4$: 636.2354; detected $[M-H]^+$: 636.2363

Nitro[azido-ethyl-N-annulated perylene diimide] (N_3-C2-NPDI-NO_2)
Precursor, N_3-C2-NPDI, was synthesized following reported literature procedure.[x]

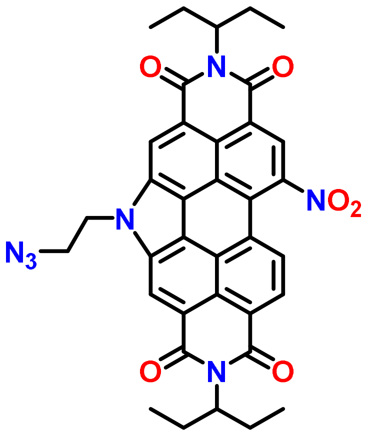

N_3-C2-NPDI (246 mg, 0.402 mmol, 1 eq.) was dissolved with CH_2Cl_2 (75 mL) in a charged 250 mL rbf. The reaction mixture was cooled to -78 °C. After 15 minutes of stirring, fuming HNO_3 (2 mL, excess) was added, and the reaction was left to stir at -78 °C for 3 hours (monitoring by TLC).* Following reaction completion, H_2O (100 mL) was poured into the reaction and the biphasic solution was vigorously stirred for 5 minutes. Next, organic extracts were liquid-liquid washed with H_2O (3 x 50 mL). The organic extracts were dried over Na_2SO_4, decanted over a silica-gel plug, and eluted with more CH_2Cl_2. After all CH_2Cl_2 was removed by rotary evaporation, the resulting red powder was

isolated by vacuum filtration by creating a slurry from (19:1) MeOH:H_2O (239 mg, 0.363 mmol, 88% yield).

*Note: the reaction must be done at cryogenic temperatures to afford the intended mono-nitrated product.

^{1}H NMR (600 MHz, $CDCl_3$) δ 9.17 (s, 1H), 9.07 (s, 1H), 8.88 (s, 1H), 8.79 (d, J = 7.9 Hz, 1H), 8.74 (d, J = 8.2 Hz, 1H), 5.22 – 5.16 (m, 2H), 5.09 (t, J = 5.5 Hz, 2H), 4.13 (dd, J = 6.2, 4.7 Hz, 2H), 2.34 (dddd, J = 15.3, 9.5, 7.5, 3.7 Hz, 4H), 2.02 (ddd, J = 13.8, 7.5, 6.0 Hz, 4H), 0.98 (t, J = 7.5 Hz, 12H).

^{13}C NMR (151 MHz, $CDCl_3$) δ 165.55, 164.69, 147.02, 135.35, 134.93, 128.42, 128.30, 127.66, 125.63, 125.20, 124.79, 123.67, 122.98, 122.71, 122.60, 122.22, 120.40, 119.63, 119.27, 118.61, 58.36, 58.11, 51.83, 46.13, 25.18, 25.10, 11.44, 11.41. *expected = 32 C; observed = 28 C; some quaternary carbons centers were not detected*

HRMS ([M-H]$^+$) calculated for M = $C_{36}H_{31}N_7O_6$: 656.2252; detected [M-H]$^+$: 656.2253

Bromo[azido-ethyl-N-annulated perylene diimide] (N_3-C2-NPDI-Br)

Precursor, N_3-C2-NPDI, was synthesized following reported literature procedure.[x]

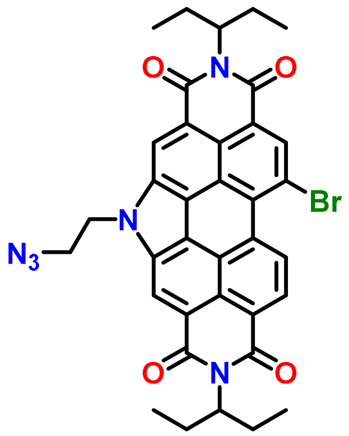

In a charged 250 mL rbf, N_3-C2-NPDI (612 mg, 0.999 mmol, 1 eq.) was dissolved with CH_2Cl_2 (40 mL), and Br_2 (1 mL, 19.5 mmol, 20 eq.) was subsequently added. Reaction progress was monitored by TLC. Upon completion, the reaction was slowly quenched using a saturated Na_2S_2 O_5 aqueous solution and then stirred vigorously for 15 minutes. The organic extracts were subsequently liquid-liquid washed with H_2O (3 x 50 mL) and then dried over Na_2SO_4. After decanting the organic extracts through a Celite plug, solvent was removed by rotary evaporation to afford a solid. The dark red solid was precipitated with a (19:1) MeOH:H_2O mixture and collected by vacuum filtration (631 mg, 0.912 mmol, 91% yield).

^{1}H NMR (600 MHz, $CDCl_3$) δ δ 9.64 (d, J = 8.2 Hz, 1H), 8.86 (d, J = 2.9 Hz, 2H), 8.79 (s, 1H), 8.59 (s, 1H), 5.23 – 5.16 (m, 2H), 4.93 (t, J = 5.5 Hz, 2H), 4.05 (dd, J = 6.3, 4.7 Hz, 2H), 2.37 (tdd, J = 14.4, 7.3, 1.9 Hz, 4H), 2.10 (dddd, J = 13.8, 11.8, 7.5, 6.2 Hz, 4H), 1.05 (dt, J = 12.0, 7.4 Hz, 12H).

^{13}C NMR (151 MHz, $CDCl_3$) δ 164.99, 164.49, 163.49, 134.45, 134.36, 134.05, 131.87, 129.98, 127.28, 127.18, 123.97, 123.16, 123.08, 122.75, 122.43, 122.22, 121.63, 121.27, 118.59, 118.20, 117.44, 58.20, 58.05, 51.66, 45.80, 25.20, 25.14, 11.64, 11.60. *expected = 32 C; observed = 29 C; some quaternary carbons centers were not detected*

HRMS ([M-H]$^+$) calculated for M = $C_{36}H_{31}N_6O_4Br$: 689.1506; detected [M-H]$^+$: 689.1538

Pyrrolidine[azido-ethyl-N-annulated perylene diimide] (N_3-C2-NPDI-NR_2)

Procedure was modified from literature.[7]

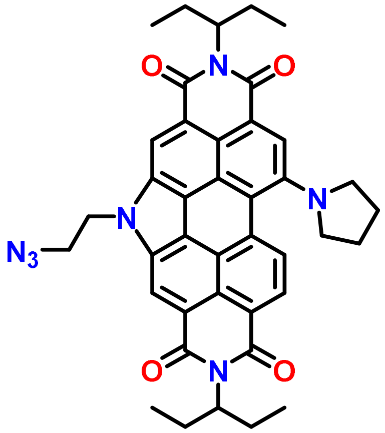

N_3-C2-NPDI-Br (282 mg, 0.408 mmol, 1 eq.) was dissolved with pyrrolidine (6 mL) in a 10 mL glass pressure vial. The vial was sealed, sparged with N_2 for 30 minutes, and then placed into a 65 °C bead bath to stir for 18 hours. Upon reaction completion (monitoring by TLC), the mixture was poured into a 1% HCl solution (50 mL) and was then subsequently liquid-liquid extracted with CH_2Cl_2 (4 x 50 mL). The organic extracts were dried over Na_2SO_4, eluted through a Celite plug, and then dried by rotary evaporation. Resulting crude solid was purified by silica-gel column chromatography (eluting with 7:1 hexanes:acetone). Common fractions were combined, dried by rotary evaporation, and collected by vacuum filtration using a (19:1) MeOH:H_2O mixture (191 mg, 0.280 mmol, 70% yield).

^{1}H NMR (600 MHz, $CDCl_3$) δ 9.03 (s, 1H), 8.91 (d, *J* = 8.2 Hz, 1H), 8.84 (s, 2H), 8.68 (s, 1H), 5.28 – 5.15 (m, 2H), 5.00 (t, *J* = 5.6 Hz, 2H), 4.10 – 4.04 (m, 2H), 3.16 (s, 4H), 2.37 (ddt, *J* = 13.9, 9.6, 7.7 Hz, 4H), 2.24 (s, 4H), 2.01 (ddt, *J* = 13.6, 7.6, 5.9 Hz, 4H), 0.99 (td, *J* = 7.5, 1.4 Hz, 12H).

^{13}C NMR (151 MHz, $CDCl_3$) δ 166.81, 165.61, 164.45, 150.97, 135.82, 134.54, 133.90, 128.50, 128.05, 126.62, 124.95, 124.40, 123.01, 122.36, 121.50, 121.16, 120.49, 120.16, 119.77, 119.29, 117.59, 117.19, 114.79, 114.71, 57.78, 57.66, 51.77, 51.06, 45.70, 25.28, 25.24, 24.78, 11.47, 11.45. *expected = 34 C; observed = 34 C*

HRMS ($[M-3H]^+$) calculated for M = $C_{40}H_{39}N_7O_4$: 678.2829; detected $[M-3H]^+$: 678.2838

Phenoxy[azido-ethyl-N-annulated perylene diimide] (N_3-C2-NPDI-OPh)

Procedure was modified from literature.[8]

N_3-C2-NPDI-Br (137 mg, 0.198 mmol, 1 eq.), K_2CO_3 (70 mg, 0.492 mmol, 2.5 eq.), and phenol (94 mg, 0.998 mmol, 5 eq.) were dissolved with anhydrous DMF (8 mL) in a 20 mL glass pressure vial. The vial was sealed, sparged with N_2 for 30 minutes, and then placed in an 80°C bead bath to stir for 18 hours. Following reaction completion (tracking by TLC), all solvent was removed by rotary evaporation. The crude solid was re-dissolved in minimal CH_2Cl_2, adhered to silica, and loaded on top of a silica-gel plug. To elute the desired product, a (6:1) hexanes:acetone mixture was used. After all solvent was removed by rotary evaporation, the afforded red solid was precipitated into H_2O and collected by vacuum filtration (132 mg, 0.187 mmol, 93% yield).

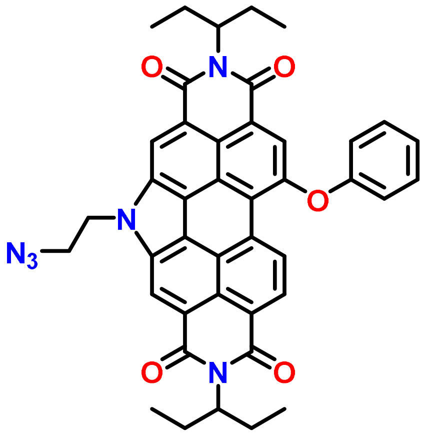

^{1}H NMR (600 MHz, $CDCl_3$) δ 9.45 (d, J = 8.1 Hz, 1H), 9.02 (s, 1H), 8.91 (s, 1H), 8.81 (d, J = 8.1 Hz, 1H), 8.30 (s, 1H), 7.57 – 7.50 (m, 2H), 7.37 – 7.29 (m, 3H), 5.28 – 5.11 (m, 2H), 5.01 (t, J = 5.6 Hz, 2H), 4.08 (dd, J = 6.3, 4.9 Hz, 2H), 2.42 – 2.24 (m, 4H), 2.08 – 1.94 (m, 4H), 1.02 – 0.95 (m, 12H).

^{13}C NMR (151 MHz, $CDCl_3$) δ 166.37, 165.31, 157.61, 155.20, 135.12, 134.36, 132.22, 130.68, 128.96, 128.72, 125.50, 124.85, 124.49, 123.77, 122.08, 121.42, 120.45, 120.23, 120.19, 119.60, 118.79, 117.90, 117.48, 116.63, 116.21, 57.94, 57.81, 51.79, 45.90, 25.25, 25.22, 11.50, 11.48. *expected = 36 C; observed = 33 C; some quaternary carbons centers were not detected*

HRMS ($[M-H]^+$) calculated for M = $C_{42}H_{36}N_6O_5$: 703.2663; detected $[M-H]^+$: 703.2635

General Preparation of Re(bpy-C2-NPDI-R) catalysts

Precursor Re(5-ethynyl-2,2'-bipyridine)$(CO)_3$Cl was synthesized following known literature procedure.[x] The conditions for copper catalyzed azide-alkyne cycloaddition are identical to those that were previously reported for Re(bpy-C2-NPDI-H).[x]

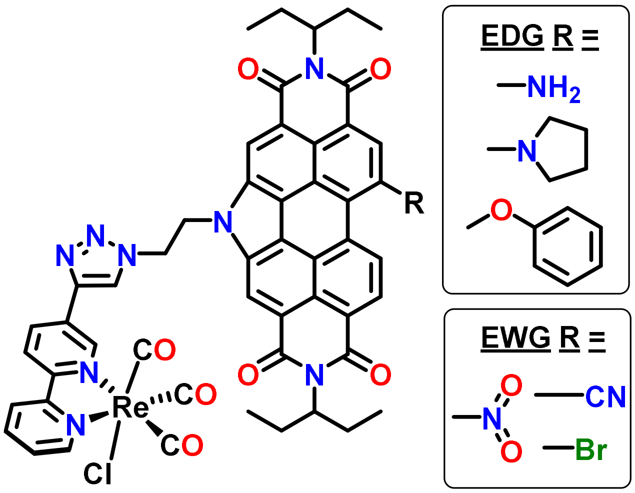

Re(5-ethynyl-2,2'-bipyridine)$(CO)_3$Cl (48.6 mg, 0.100 mmol, 1eq.) and N_3-C2-NPDI-R (0.100 mmol, 1 eq.) were combined in a 10 mL glass pressure vial with $CuSO_4$ (1.6 mg, 0.010 mmol, 0.1 eq.) and sodium ascorbate (5.9 mg, 0.030 mmol, 0.3 eq.). The vial was sealed and then N_2-sparged for 30 minutes. A degassed mixture of (4:1) THF:H_2O (7.5 mL) was transferred into the vial using a Cannula line. The mixture was purged an additional 5 minutes, then placed into a 60 °C bead bath for 18 hours. After TLC showed consumption of all starting material, all solvent was removed by rotary evaporation. The resulting crude solid(s) was purified by silica-gel column chromatography, eluting with (4:3) hexanes:acetone. Solvent was removed by rotary evaporation and the resulting solid(s) was collected by vacuum filtration by precipitating into a (19:1) MeOH:H_2O mixture.

Re(bpy-C2-NPDI-CN) (74.0 mg, 0.067 mmol, 67% yield)

^{1}H NMR (600 MHz, TCE-d_2) δ 9.33 (d, J = 8.1 Hz, 1H), 8.41 (s, 1H), 8.38 – 8.35 (m, 1H), 8.34 (d, J = 5.4 Hz, 1H), 8.28 (s, 2H), 8.19 (s, 1H), 7.88 (d, J = 8.5 Hz, 1H), 7.52 (t, J = 7.4 Hz, 2H), 7.48 – 7.41 (m, 1H), 7.26 (s, 1H), 6.95 – 6.88 (m, 1H), 5.01 – 4.93 (m, 2H), 4.66 – 4.56 (m, 2H), 4.53 – 4.43 (m, 2H), 1.68 – 1.54 (m, 4H), 1.33 (dt, J = 14.0, 6.9 Hz, 4H), 0.27 (qd, J = 7.3, 3.6 Hz, 11H).

^{13}C NMR (151 MHz, TCE-d_2) *due to poor solubility of the material, no data was collected*

HRMS ($[M-Cl]^+$) calculated for M = $C_{52}H_{39}N_9O_7ReCl$: 1086.2497; detected $[M-Cl]^+$: 1086.2462

CHN theoretical (%) C: 55.59, H: 3.50, N: 11.22; found (%) C: 55.38 H: 3.85, N: 10.55;

UV-vis λ (x 10^4 M^{-1} cm^{-1}): 309 nm (4.1); 343 nm (3.1); 464 nm (2.5); 493 nm (3.8); 527 nm (6.2)

FTIR ν_{co} (cm^{-1}): 1901, 1923, and 2026 in $CHCl_3$; 1895, 1914, and 2019 in DMF.

Re(bpy-C2-NPDI-NO_2) (42.2 mg, 0.037 mmol, 37% yield) *low yield was observed under these conditions because of the *in-situ* reduction of -NO_2 to -NH_2 by sodium ascorbate. See modified procedure (below) for improved synthetic procedure*

^{1}H NMR (600 MHz, TCE-d_2) δ 8.38 (d, *J* = 2.0 Hz, 1H), 8.34 (dt, *J* = 5.1, 1.3 Hz, 1H), 8.28 – 8.10 (m, 5H), 7.87 (d, *J* = 8.4 Hz, 1H), 7.52 (t, *J* = 8.1 Hz, 2H), 7.46 (td, *J* = 7.8, 1.6 Hz, 1H), 7.25 (s, 1H), 6.91 (ddd, *J* = 7.6, 5.4, 1.3 Hz, 1H), 4.98 (q, *J* = 6.7 Hz, 2H), 4.66 – 4.55 (m, 2H), 4.47 (s, 2H), 1.67 – 1.57 (m, 4H), 1.32 (dp, *J* = 13.9, 7.1 Hz, 4H), 0.26 (tdd, *J* = 7.4, 3.7, 2.0 Hz, 12H).

^{13}C NMR (151 MHz, TCE-d_2) *due to poor solubility of the material, no data was collected*

HRMS ($[M-Cl]^+$) calculated for M = $C_{51}H_{39}N_9O_9ReCl$: 1106.2395; detected $[M-Cl]^+$: 1106.2383

CHN theoretical (%) C: 53.57, H: 3.44, N: 11.02; found (%) C: 53.17 H: 3.57, N: 10.58;

UV-vis λ (x 10^4 M^{-1} cm^{-1}): 309 nm (3.9); 343 nm (2.1); 467 nm (1.9); 498 nm (3.3); 534 nm (6.1)

FTIR ν_{co} (cm^{-1}): 1901, 1925, and 2026 in $CHCl_3$; 1894, 1915, and 2019 in DMF.

Re(bpy-C2-NPDI-Br) (82.2 mg, 0.071 mmol, 71% yield)

^{1}H NMR (600 MHz, TCE-d_2)) δ 9.61 (d, *J* = 8.3 Hz, 1H), 8.44 – 8.37 (m, 2H), 8.34 (dd, *J* = 5.6, 1.6 Hz, 1H), 8.24 (s, 1H), 8.13 (s, 2H), 7.83 (d, *J* = 8.4 Hz, 1H), 7.51 (dd, *J* = 12.4, 8.4 Hz, 2H), 7.44 (td, *J* = 7.9, 1.6 Hz, 1H), 7.26 (s, 1H), 6.91 (ddd, *J* = 7.1, 5.4, 1.2 Hz, 1H), 4.97 – 4.83 (m, 2H), 4.67 – 4.53 (m, 2H), 4.52 – 4.40 (m, 2H), 1.60 (m, 4H), 1.33 (tt, *J* = 14.1, 6.5 Hz, 4H), 0.31 – 0.23 (m, 12H).

^{13}C NMR (151 MHz, TCE-d_2) *due to poor solubility of the material, no data was collected*

HRMS ($[M-Cl]^+$) calculated for M = $C_{51}H_{39}N_8O_7ReClBr$: 1139.1649; detected $[M-Cl]^+$: 1139.1609

CHN theoretical (%) C: 52.02, H: 3.34, N: 9.52; found (%) C: 52.38 H: 3.69, N: 8.99;

UV-vis λ (x 10^4 M^{-1} cm^{-1}): 313 nm (2.6); 343 nm (1.6); 459 nm (2.0); 488 nm (3.6); 522 nm (6.4)

FTIR ν_{co} (cm^{-1}): 1899, 1923, and 2026 in $CHCl_3$; 1894, 1915, and 2019 in DMF.

Re(bpy-C2-NPDI-NR$_2$) (107 mg, 0.084 mmol, 84% yield)

^{1}H NMR (600 MHz, $CDCl_3$) δ 9.01 (t, J = 4.7 Hz, 2H), 8.87 (d, J = 8.0 Hz, 1H), 8.82 (s, 2H), 8.67 (s, 1H), 8.61 (s, 1H), 8.45 (dd, J = 8.5, 2.0 Hz, 1H), 8.16 (d, J = 8.2 Hz, 1H), 8.12 (d, J = 8.5 Hz, 1H), 8.06 – 8.00 (m, 1H), 7.92 (s, 1H), 7.52 (t, J = 6.6 Hz, 1H), 5.52 (dq, J = 15.8, 8.3 Hz, 2H), 5.30 (s, 2H), 5.22 – 5.12 (m, 2H), 3.92 (s, 2H), 3.16 (s, 2H), 2.30 (s, 4H), 2.23 (s, 4H), 1.95 (dh, J = 14.6, 7.4 Hz, 4H), 0.91 (td, J = 7.5, 4.1 Hz, 12H).

^{13}C NMR (151 MHz, $CDCl_3$) δ 197.17, 196.76, 189.00, 165.36, 155.27, 154.38, 153.12, 151.06, 149.60, 142.27, 139.02, 135.76, 135.69, 134.35, 134.10, 130.16, 127.00, 126.69, 125.08, 124.55, 123.23, 123.17, 123.16, 122.64, 121.44, 121.25, 120.35, 119.74, 57.69, 57.57, 51.13, 51.11, 46.24, 25.20, 25.18, 25.17, 25.15, 24.83, 11.43, 11.42, 11.40. *expected = 49 C; observed = 41 C; some quaternary carbons centers were not detected*

HRMS ($[M-Cl]^+$) calculated for M = $C_{55}H_{46}N_9O_7ReCl$: 1164.2733; detected $[M-Cl]^+$: 1164.2778

CHN theoretical (%) C: 56.57, H: 4.06, N: 10.80; found (%) C: 56.15 H: 4.05, N: 10.31;

UV-vis λ (x 10^4 M^{-1} cm^{-1}): 304 nm (4.4); 343 nm (2.4); 455 nm (1.8); 484 nm (3.2); 572 nm (3.1)

FTIR ν_{co} (cm^{-1}): 1901, 1925, and 2026 in $CHCl_3$; 1895, 1916, and 2019 in DMF.

Re(bpy-C2-NPDI-OPh) (100 mg, 0.084 mmol, 84% yield)

^{1}H NMR (600 MHz, $CDCl_3$) δ 9.47 (d, J = 8.1 Hz, 1H), 9.08 – 9.01 (m, 2H), 8.80 (s, 2H), 8.67 (s, 1H), 8.46 (d, J = 8.4 Hz, 1H), 8.30 (s, 1H), 8.17 (d, J = 8.1 Hz, 1H), 8.13 (d, J = 8.4 Hz, 1H), 8.04 (t, J = 7.9 Hz, 1H), 7.99 (s, 1H), 7.53 (t, J = 8.0 Hz, 3H), 7.35 (t, J = 7.5 Hz, 1H), 7.31 (d, J = 7.9 Hz, 2H), 5.58 – 5.49 (m, 2H), 5.35 – 5.27 (m, 2H), 5.21 – 5.06 (m, 2H), 2.35 – 2.20 (m, 4H), 2.01 – 1.90 (m, 4H), 0.96 – 0.87 (m, 12H).

^{13}C NMR (151 MHz, $CDCl_3$) δ 197.26, 196.76, 188.99, 157.86, 155.26, 155.09, 154.46, 153.16, 149.66, 142.41, 139.05, 135.78, 134.92, 134.15, 132.44, 130.68, 130.11, 129.02, 127.03, 125.54, 124.56, 123.89, 123.24, 123.19, 122.68, 122.19, 121.49, 120.50, 120.40, 120.29, 119.79, 57.84, 57.71, 51.19, 46.47, 25.17, 25.15, 25.12, 11.46, 11.43. *expected = 51 C; observed = 40 C; some quaternary carbons centers were not detected*

HRMS ($[M-Cl]^+$) calculated for M = $C_{57}H_{44}N_8O_8ReCl$: 1153.2806; detected $[M-Cl]^+$: 1153.2858

CHN theoretical (%) C: 57.50, H: 3.72, N: 9.41; found (%) C: 56.89 H: 4.07, N: 9.06;

UV-vis λ (x 10^4 M^{-1} cm^{-1}): 316 nm (2.9); 342 nm (1.9); 465 nm (2.5); 496 nm (3.8); 531 nm (6.5)

FTIR ν_{co} (cm^{-1}): 1901, 1923, and 2026 in $CHCl_3$; 1893, 1914, and 2019 in DMF.

Selective Preparation of Re(bpy-C2-NPDI-NO_2)

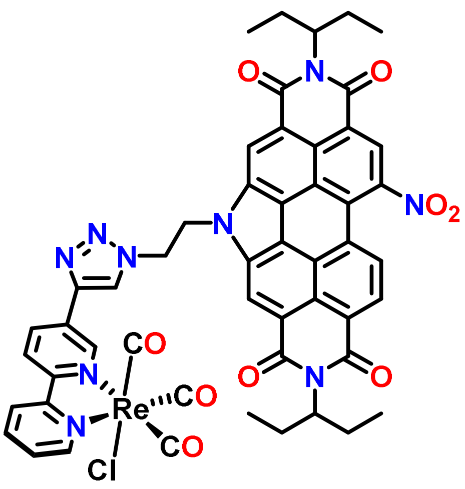

Re(5-ethynyl-2,2'-bipyridine)$(CO)_3Cl$ (48.6 mg, 0.100 mmol, 1eq.) and N_3-C2-NPDI-NO_2 (65.8 mg, 0.100 mmol, 1 eq.) were combined in a 10 mL glass pressure vial with CuI (1.9 mg, 0.010 mmol, 0.1 eq.). The vial was sealed and then N_2-sparged for 30 minutes. A degassed mixture of (4:1) THF:H_2O (7.5 mL) was transferred into the vial using a Cannula line. The mixture was purged an additional 5 minutes, then placed into a 60 ºC bead bath for 18 hours. After TLC showed consumption of all starting material, all solvent was removed by rotary evaporation. The resulting crude solid(s) was purified by silica-gel column chromatography, eluting with (4:3) hexanes:acetone. Solvent was removed by rotary evaporation and the resulting solid(s) was collected by vacuum filtration by precipitating into a (19:1) MeOH:H_2O mixture (86.1 mg, 0.076 mmol, 76% yield).

Selective Preparation of Re(bpy-C2-NPDI-NH_2)

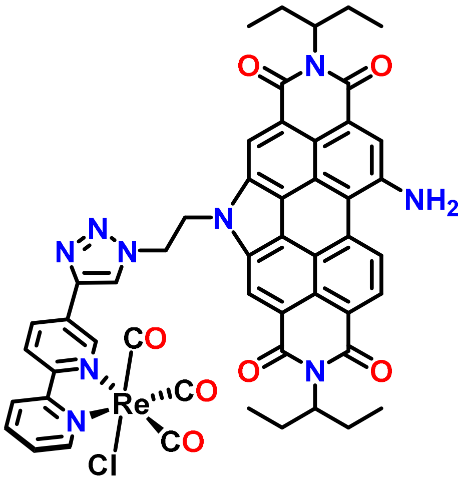

Re(5-ethynyl-2,2'-bipyridine)$(CO)_3Cl$ (48.6 mg, 0.100 mmol, 1eq.) and N_3-C2-NPDI-NO_2 (65.8 mg, 0.100 mmol, 1 eq.) were combined in a 10 mL glass pressure vial with $CuSO_4$ (1.6 mg, 0.010 mmol, 0.1 eq.) and sodium ascorbate (59.5 mg, 0.300 mmol, 3 eq.). The vial was sealed and then N_2-sparged for 30 minutes. A degassed mixture of (4:1) THF:H_2O (7.5 mL) was transferred into the vial using a Cannula line. The mixture was purged an additional 5 minutes, then placed into a 60 ºC bead bath for 18 hours. After TLC showed consumption of all starting material, all solvent was removed by rotary evaporation. The resulting crude solid was re-dissolved in CH_2Cl_2, adhered to silica, and then purified using a short silica plug. The plug was eluted first using (4:1) hexanes:acetone, followed by flashing the plug with (1:1) hexanes:acetone to afford the desired compound(s). Solvent was removed by rotary evaporation and the resulting purple solid was precipitated into a (19:1) MeOH:H_2O mixture, and then collected by vacuum filtration (90.9 mg, 0.082 mmol, 82% yield).

^{1}H NMR (600 MHz, TCE-d_2) δ 8.40 – 8.29 (m, 2H), 8.17 (s, 2H), 8.11 (s, 1H), 7.83 (d, J = 10.0 Hz, 1H), 7.58 (s, 1H), 7.50 (dd, J = 14.6, 8.3 Hz, 2H), 7.44 (t, J = 7.9 Hz, 1H), 7.17 (s, 1H), 6.90 (t, J = 6.6 Hz, 1H), 4.90 – 4.81 (m, 2H), 4.77 (s, 2H), 4.62 – 4.53 (m, 2H), 4.51 – 4.41 (m, 2H), 1.70 – 1.54 (m, 4H), 1.31 (dt, J = 13.9, 7.6 Hz, 4H), 0.26 (tt, J = 7.7, 4.0 Hz, 12H).

^{13}C NMR (151 MHz, TCE-d_2) *due to poor solubility of the material, no data was collected*

HRMS ($[M-Cl]^+$) calculated for M = $C_{51}H_{41}N_9O_7ReCl$: 1076.2653; detected $[M-Cl]^+$: 1076.2679

CHN theoretical (%) C: 55.01, H: 3.71, N: 11.32; found (%) C: 53.87 H: 3.95, N: 10.35;

UV-vis λ (x 10^4 M^{-1} cm^{-1}): 321 nm (2.6); 343 nm (1.8); 472 nm (2.2); 527 nm (2.6); 558 nm (3.4)

FTIR v_{co} (cm^{-1}): 1903, 1925, and 2026 in $CHCl_3$; 1894, 1915, and 2019 in DMF.

3. ^{1}H & ^{13}C NMR Spectroscopies

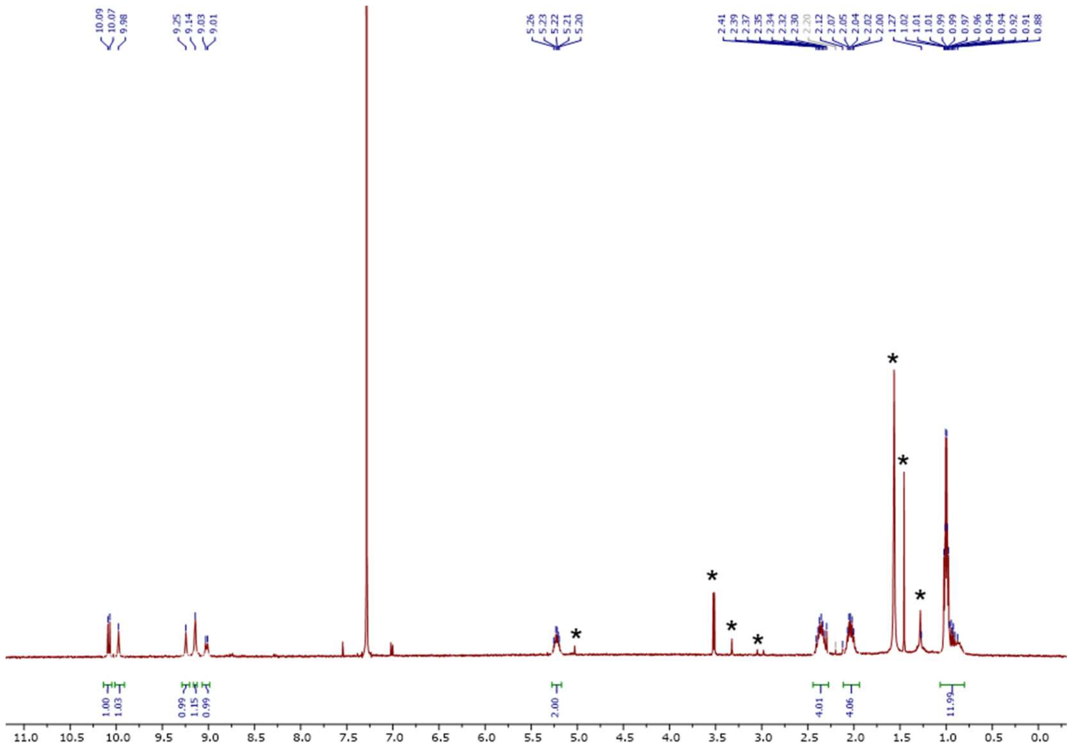

Figure S1. ^{1}H NMR spectrum of HNPDI-CN (600 MHz, $CDCl_3$); * = solvent impurities [9]

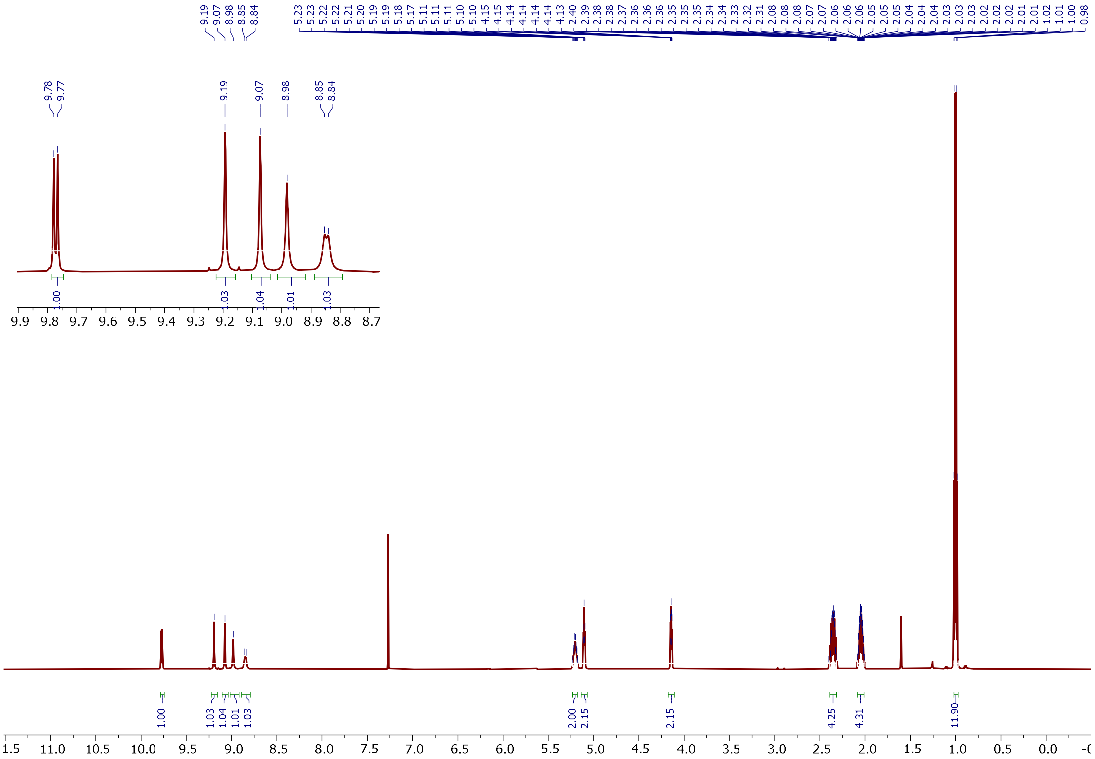

Figure S2. ^{1}H NMR spectrum of N_3-C2-NPDI-CN (600 MHz, $CDCl_3$)

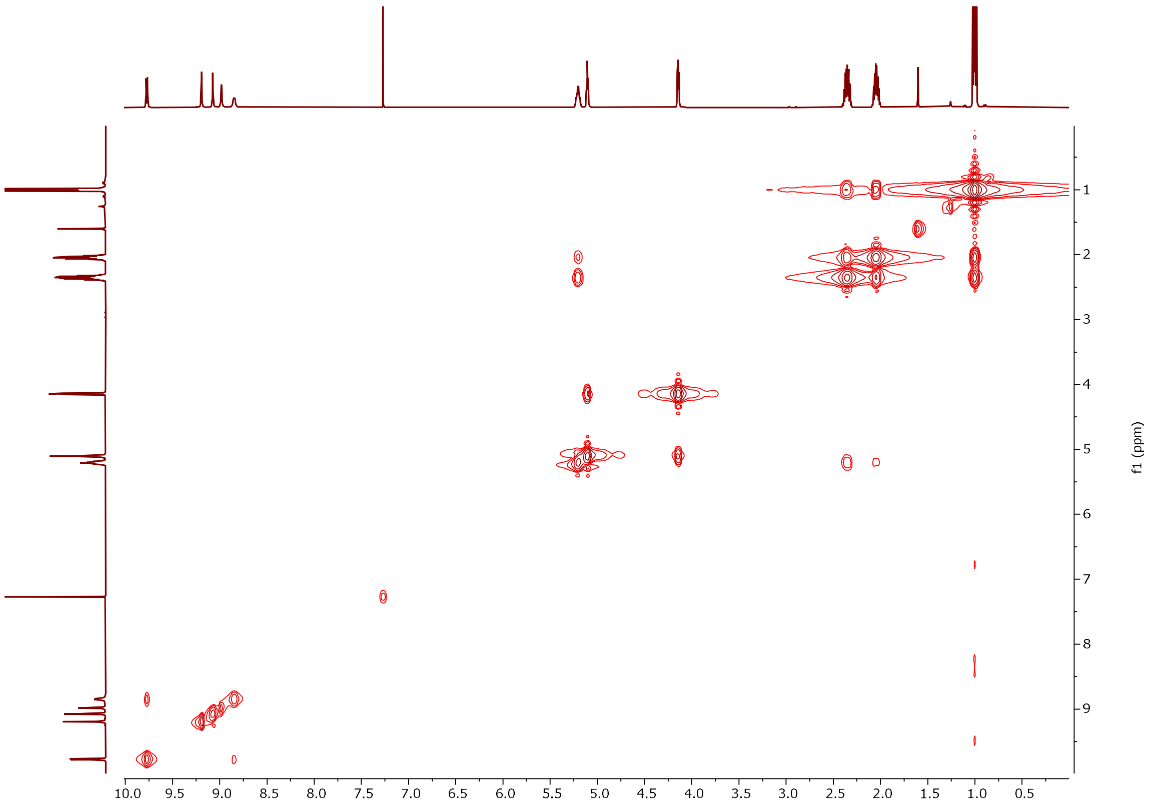

Figure S3. ^{1}H-^{1}H COSY spectrum of N_3-C2-NPDI-CN (600 MHz, $CDCl_3$)

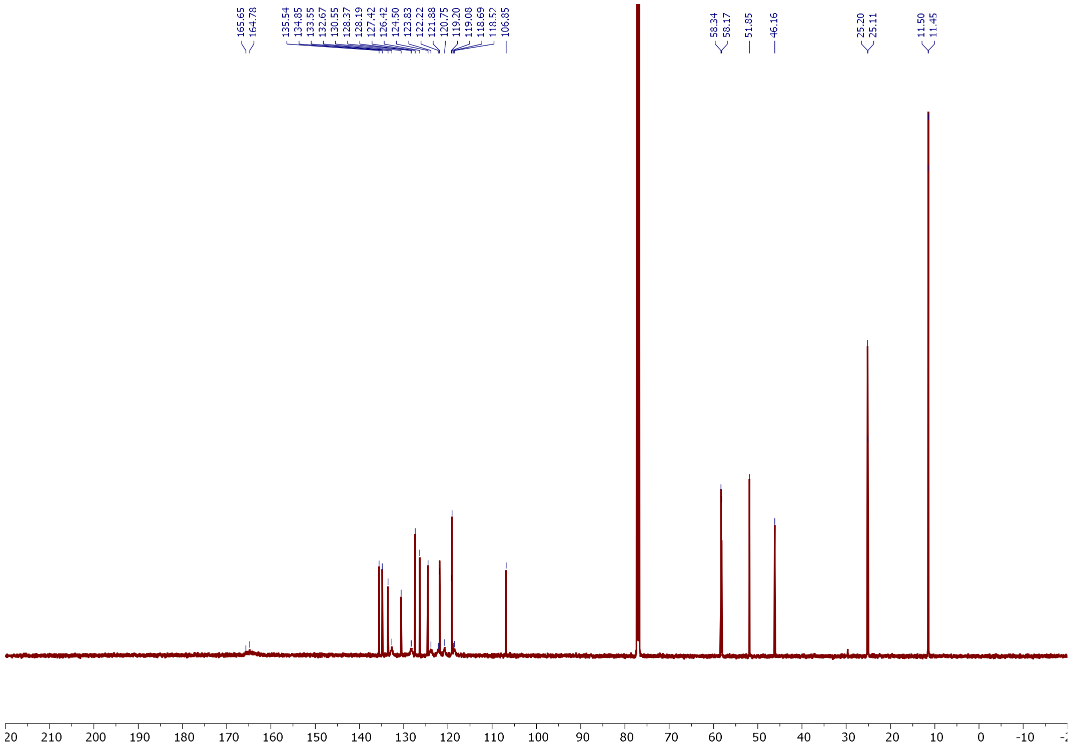

Figure S4. $^{13}C\{^{1}H\}$ NMR spectrum of N_3-C2-NPDI-CN (151 MHz, $CDCl_3$)

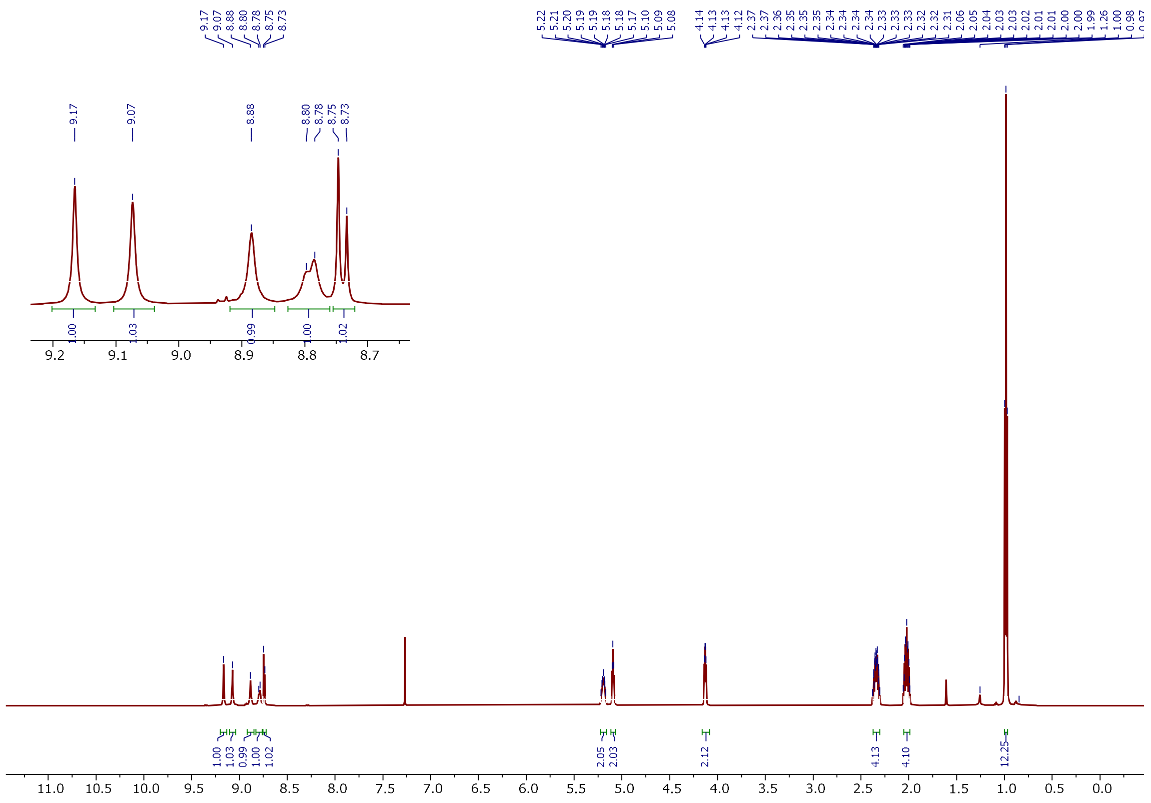

Figure S5. ^{1}H NMR spectrum of N_3-C2-NPDI-NO_2 (600 MHz, $CDCl_3$)

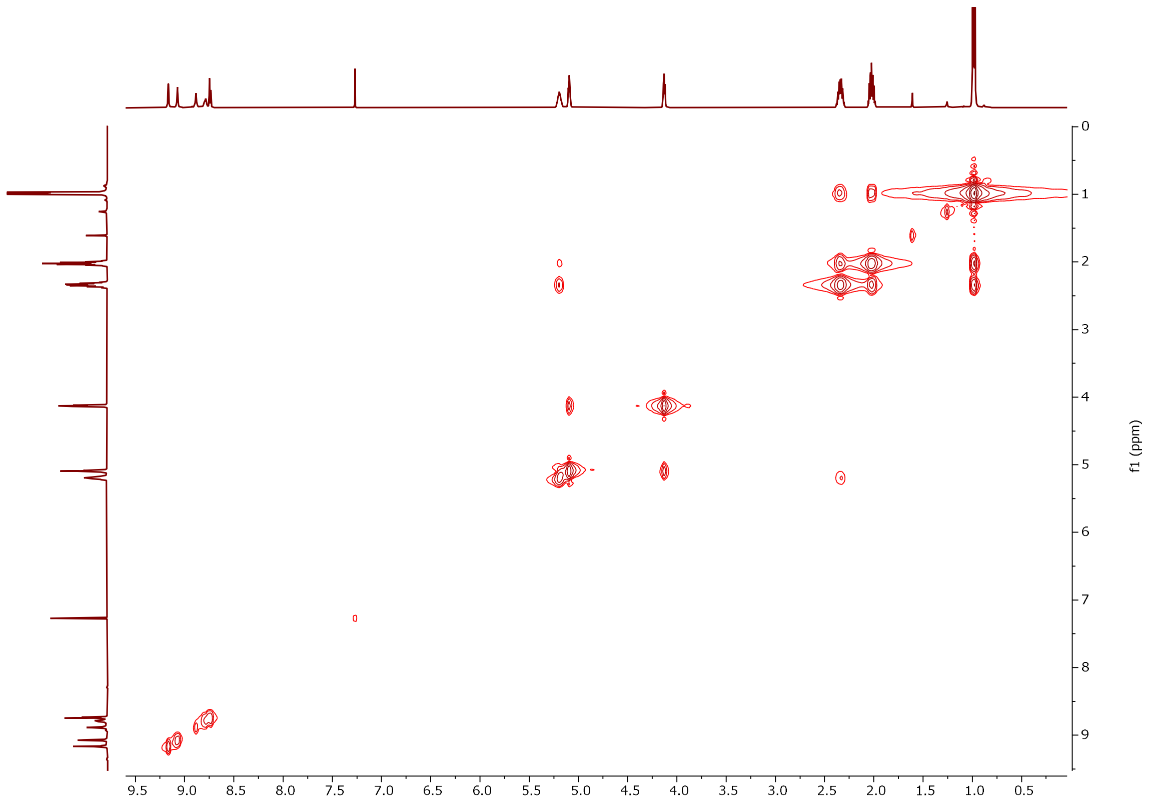

Figure S6. ^{1}H-^{1}H COSY spectrum of N_3-C2-NPDI-NO_2 (600 MHz, $CDCl_3$)

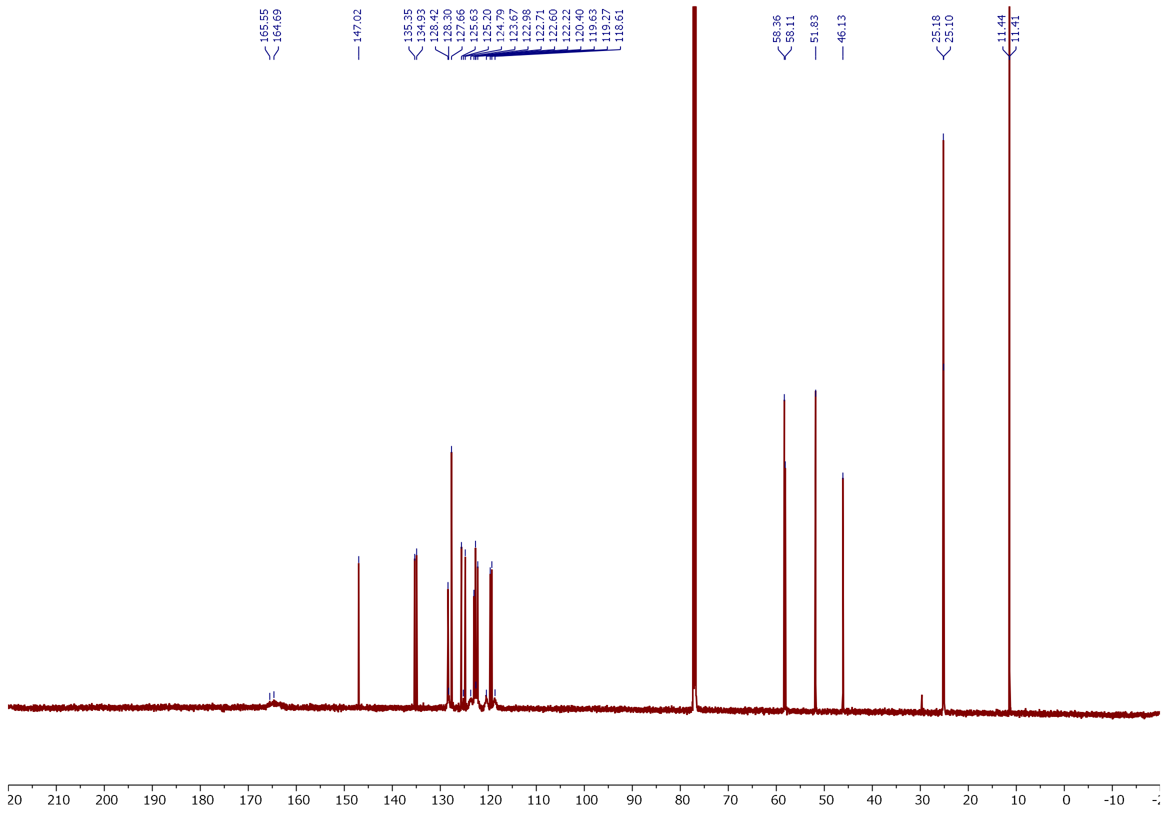

Figure S7. $^{13}C\{^{1}H\}$ NMR spectrum of N_3-C2-NPDI-NO_2 (151 MHz, $CDCl_3$)

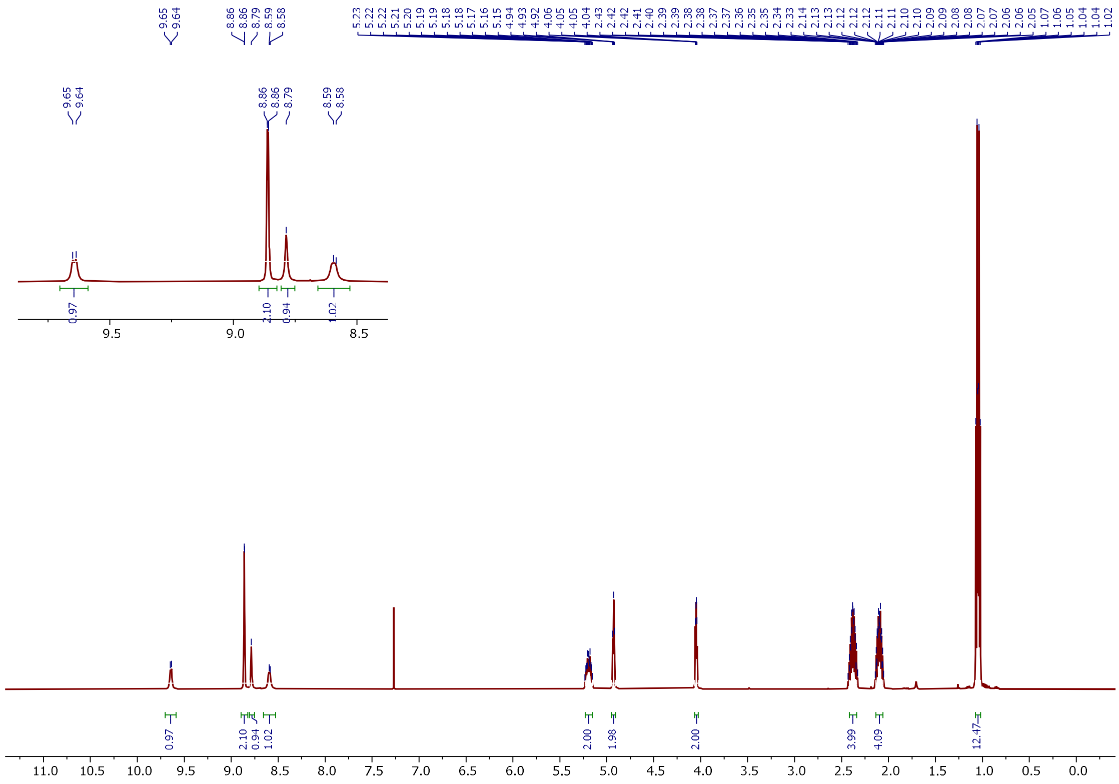

Figure S8. ^{1}H NMR spectrum of N_3-C2-NPDI-Br (600 MHz, $CDCl_3$)

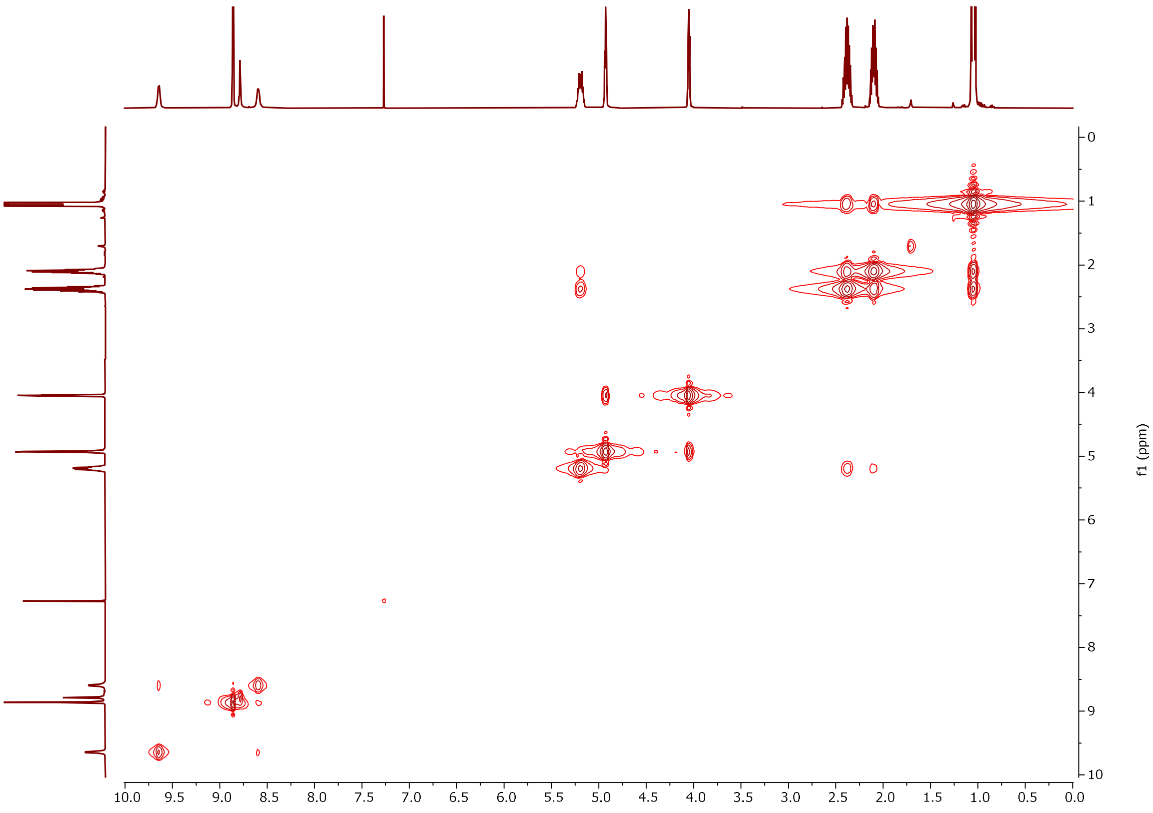

Figure S9. ^{1}H-^{1}H COSY spectrum of N_3-C2-NPDI-Br (600 MHz, $CDCl_3$)

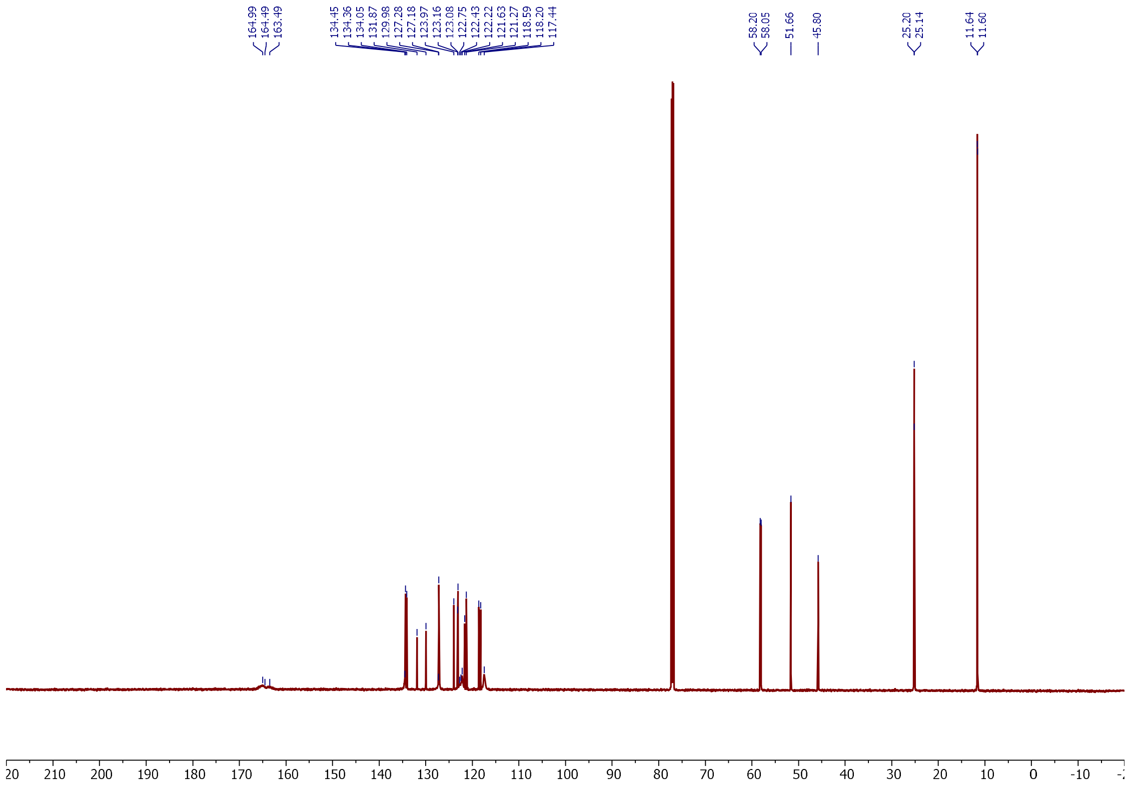

Figure S10. $^{13}C\{^{1}H\}$ NMR spectrum of N_3-C2-NPDI-Br (151 MHz, $CDCl_3$)

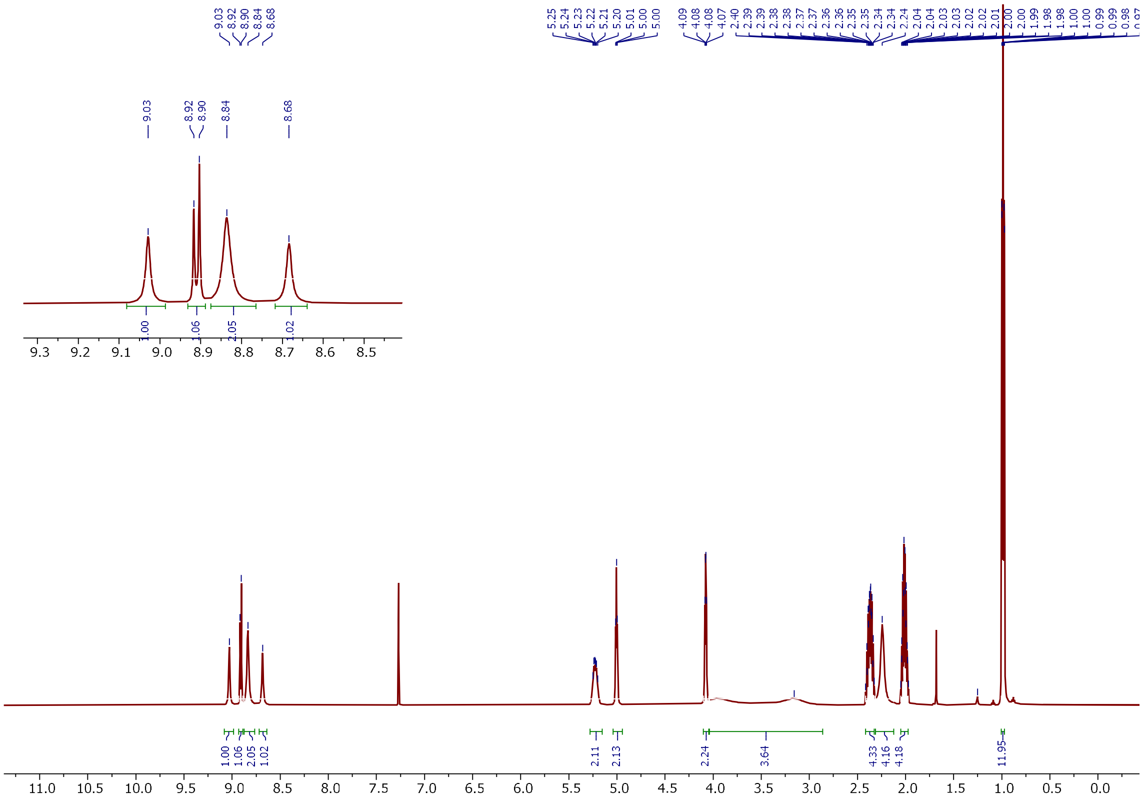

Figure S11. ^{1}H NMR spectrum of N_3-C2-NPDI-NR_2 (600 MHz, $CDCl_3$)

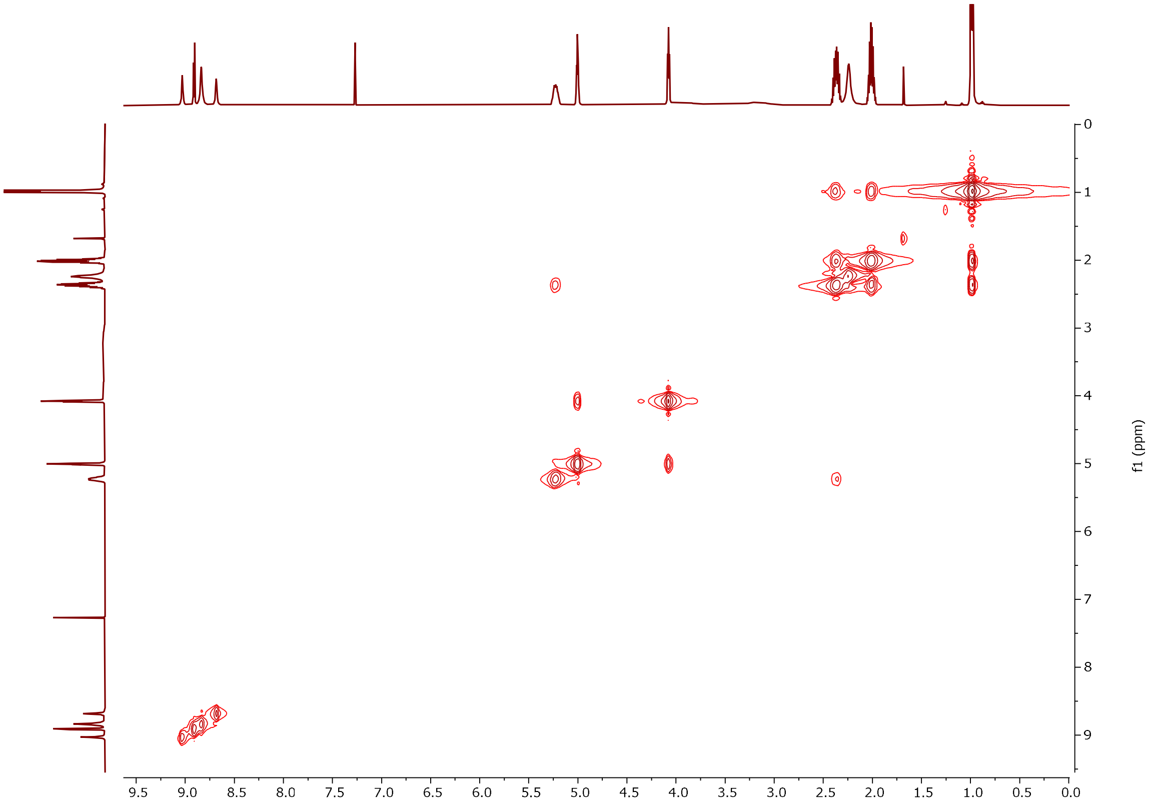

Figure S12. ^{1}H-^{1}H COSY spectrum of N_3-C2-NPDI-NR_2 (600 MHz, $CDCl_3$)

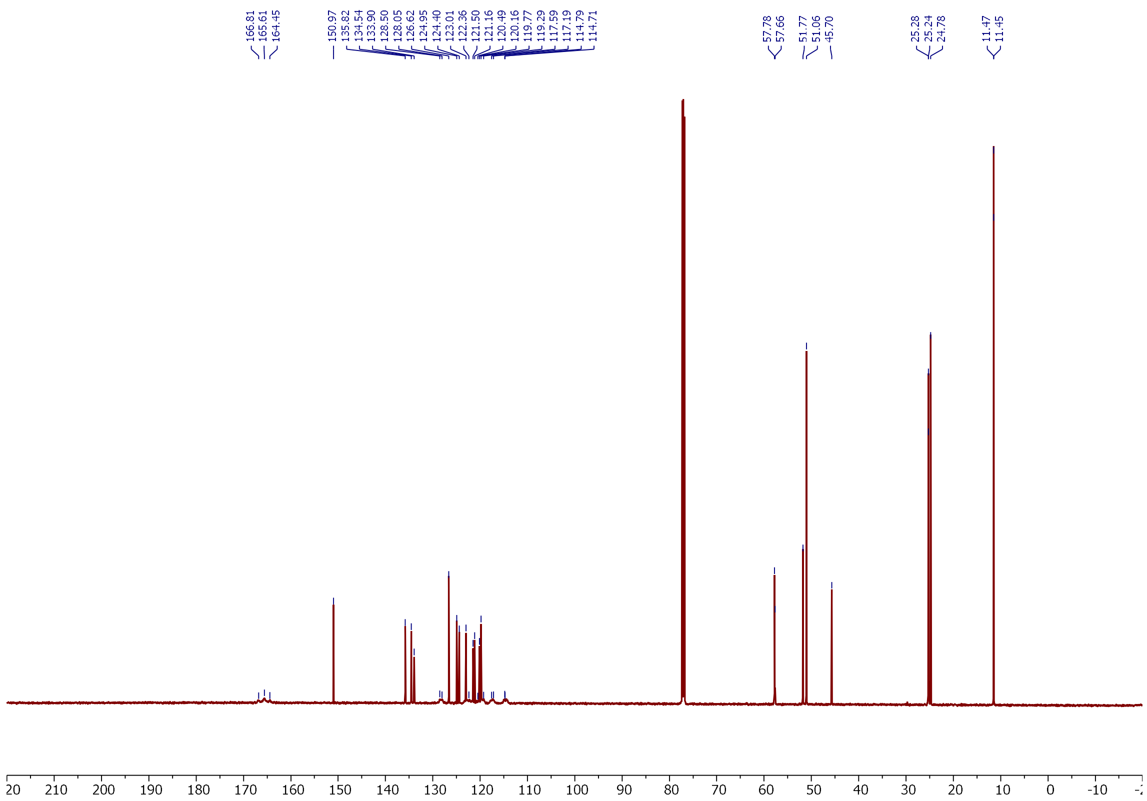

Figure S13. $^{13}C\{^{1}H\}$ NMR spectrum of N_3-C2-NPDI-NR_2 (151 MHz, $CDCl_3$)

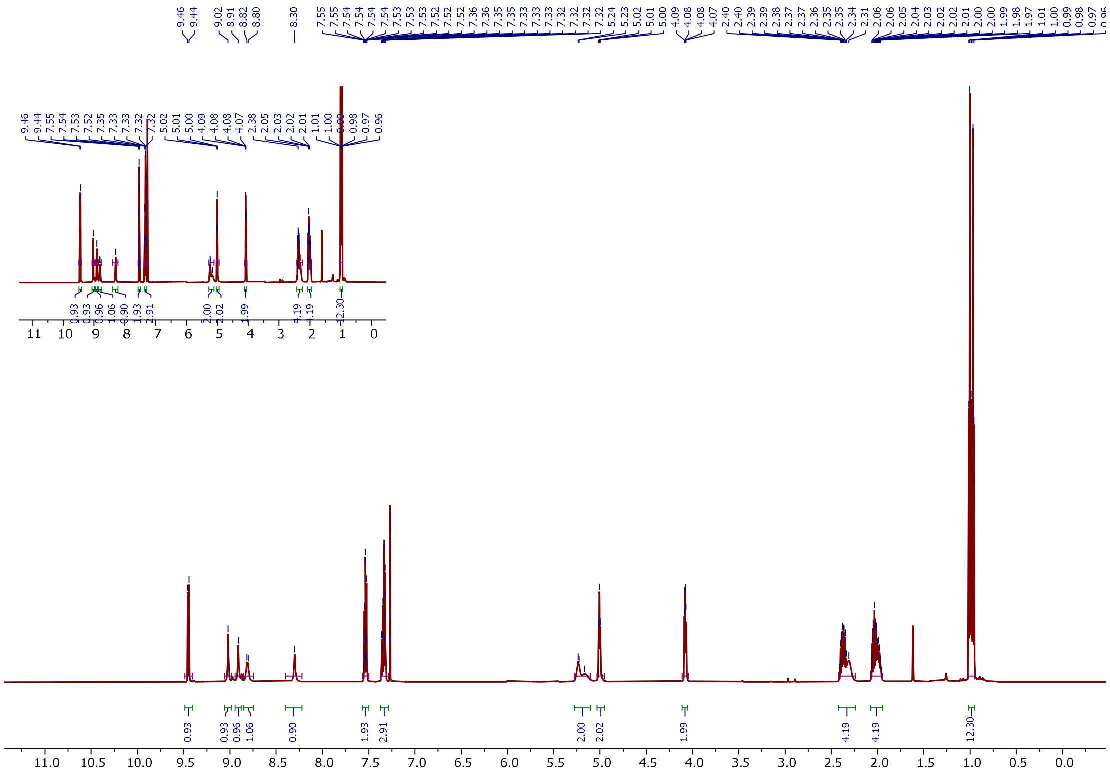

Figure S14. ^{1}H NMR spectrum of N_3-C2-NPDI-OPh (600 MHz, $CDCl_3$)

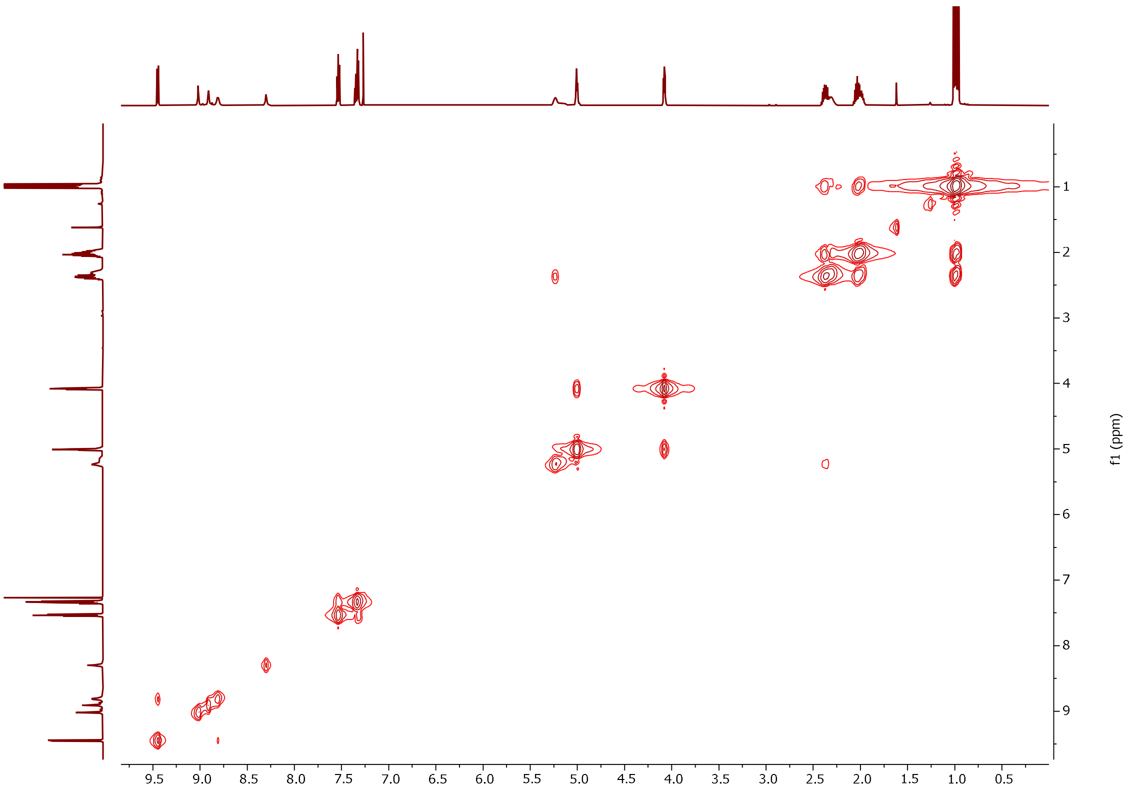

Figure S15. ^{1}H-^{1}H COSY spectrum of N_3-C2-NPDI-OPh (600 MHz, $CDCl_3$)

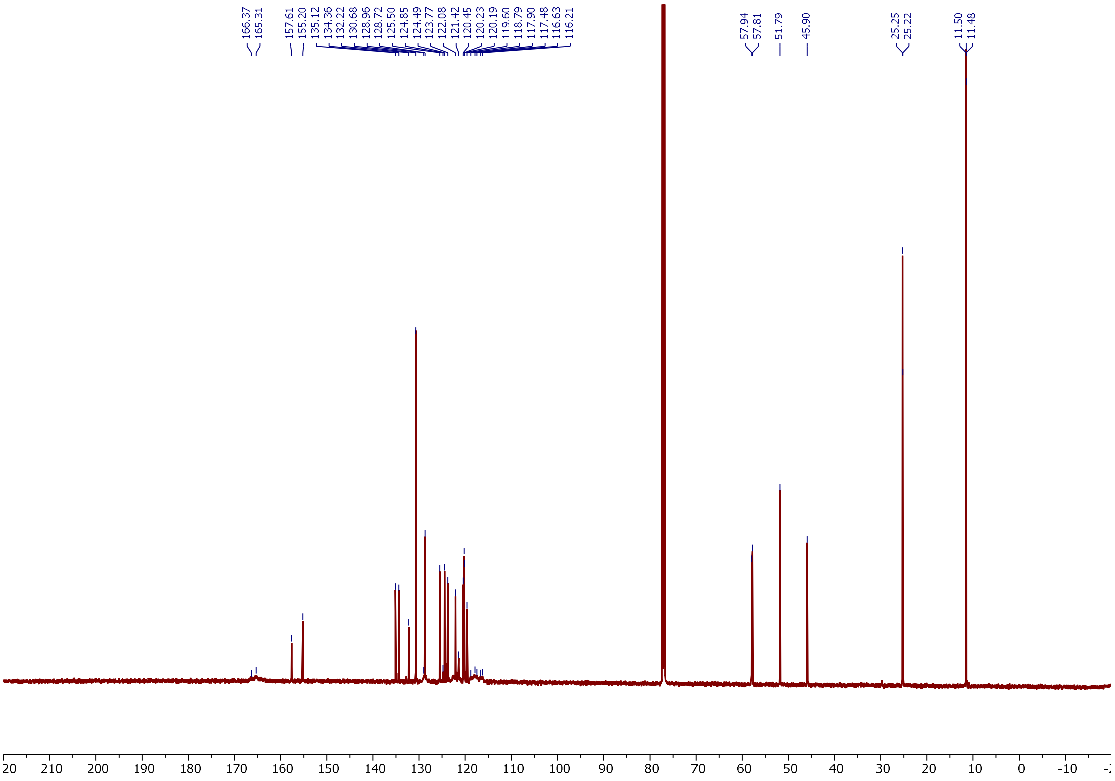

Figure S16. $^{13}C\{^{1}H\}$ NMR spectrum of N_3-C2-NPDI-OPh (151 MHz, $CDCl_3$)

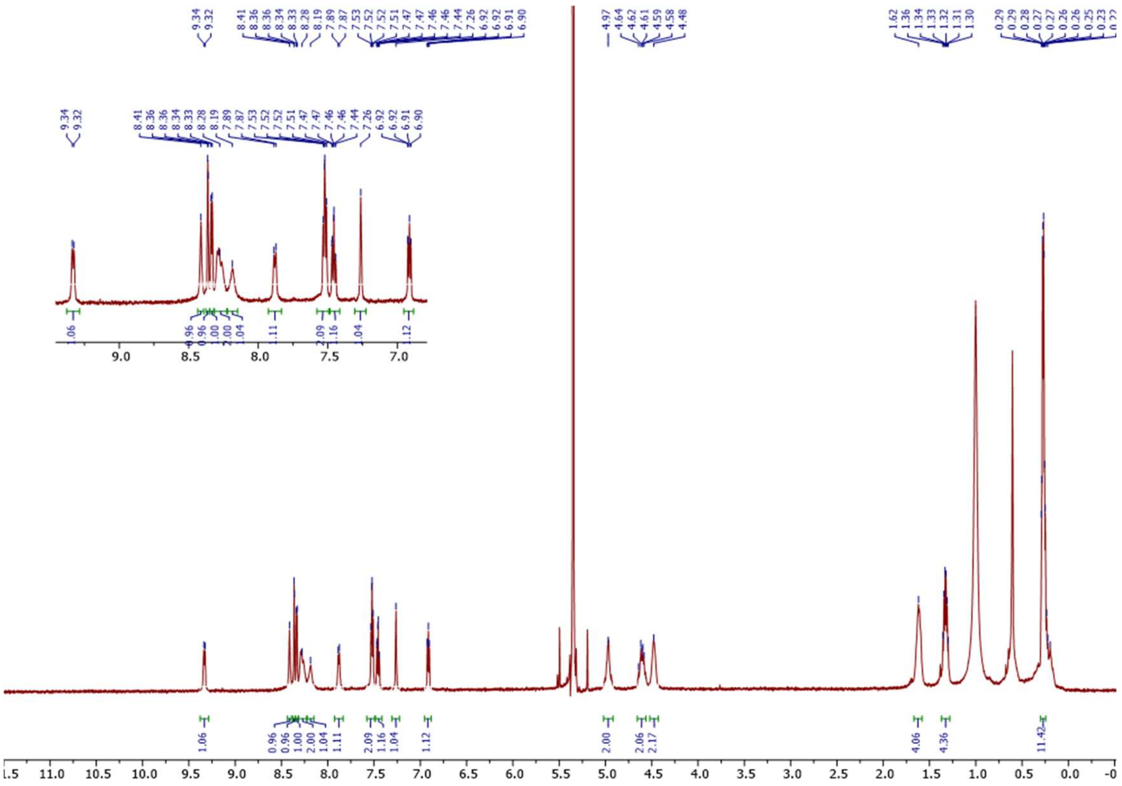

Figure S17. ^{1}H NMR spectrum of Re(bpy-C2-NPDI-CN) (600 MHz, TCE-d_2)

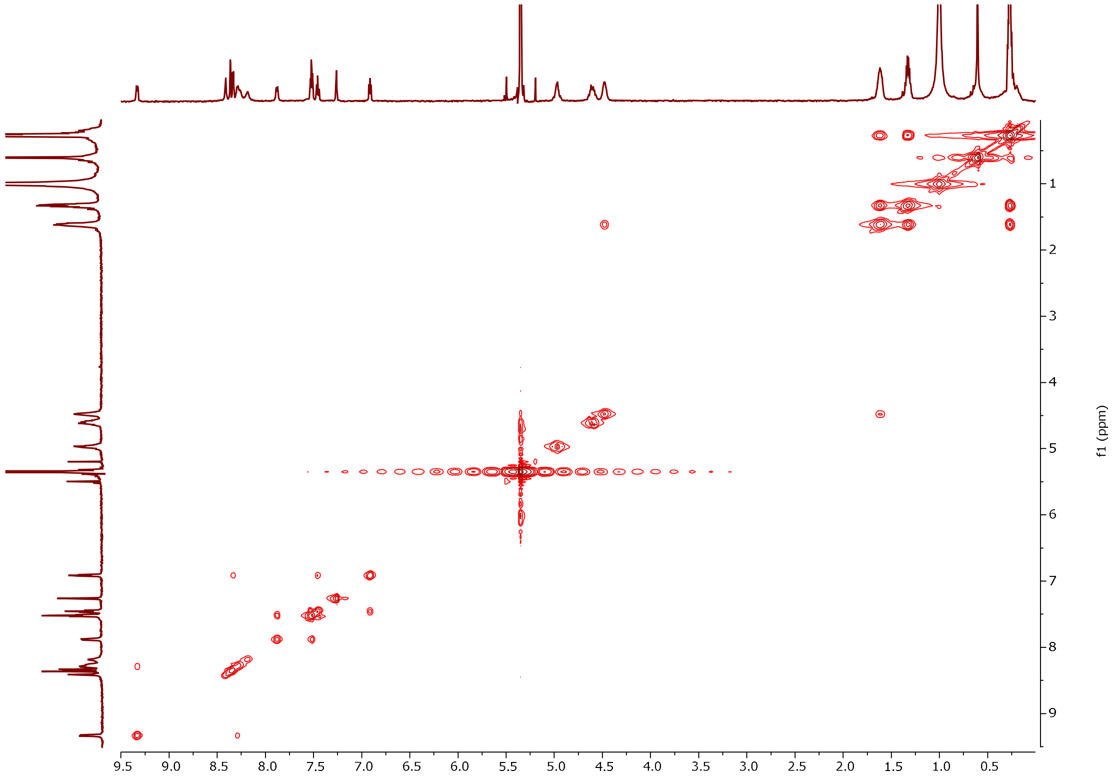

Figure S18. ^{1}H-^{1}H COSY spectrum of Re(bpy-C2-NPDI-CN) (600 MHz, TCE-d_2)

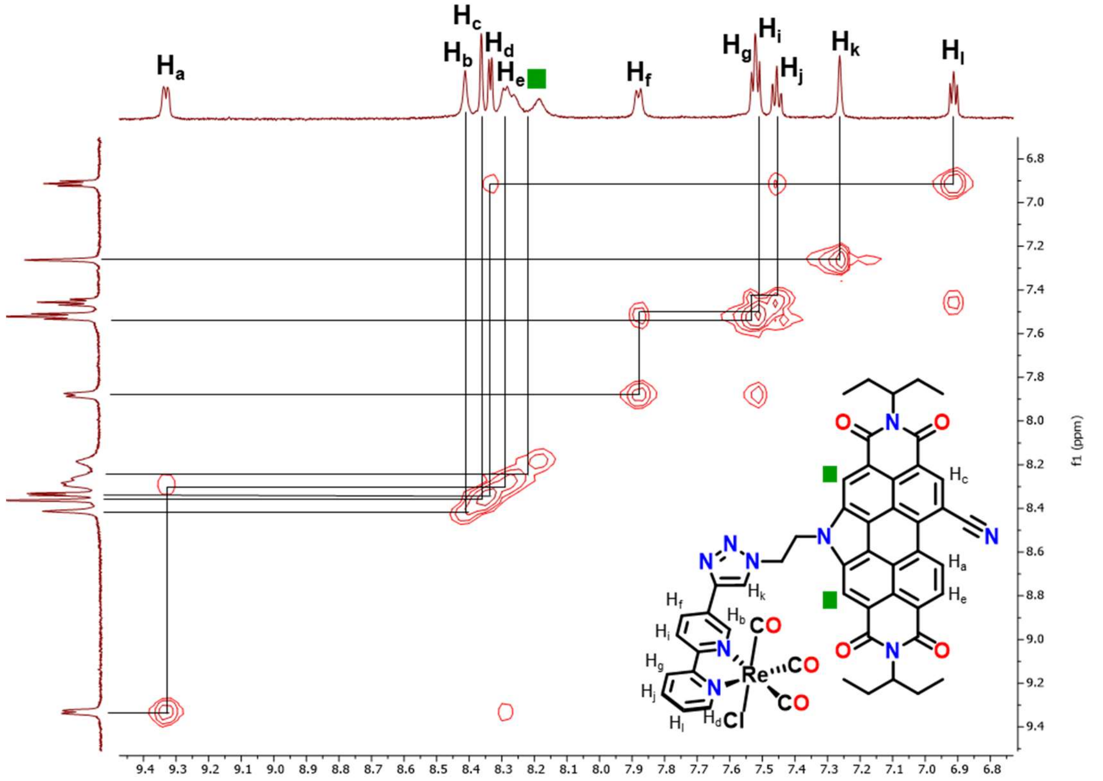

Figure S19. Enhanced view and assignment of aromatic protons in the ^1H-^1H COSY spectrum of Re(bpy-C2-NPDI-CN) (600 MHz, TCE-d$_2$)

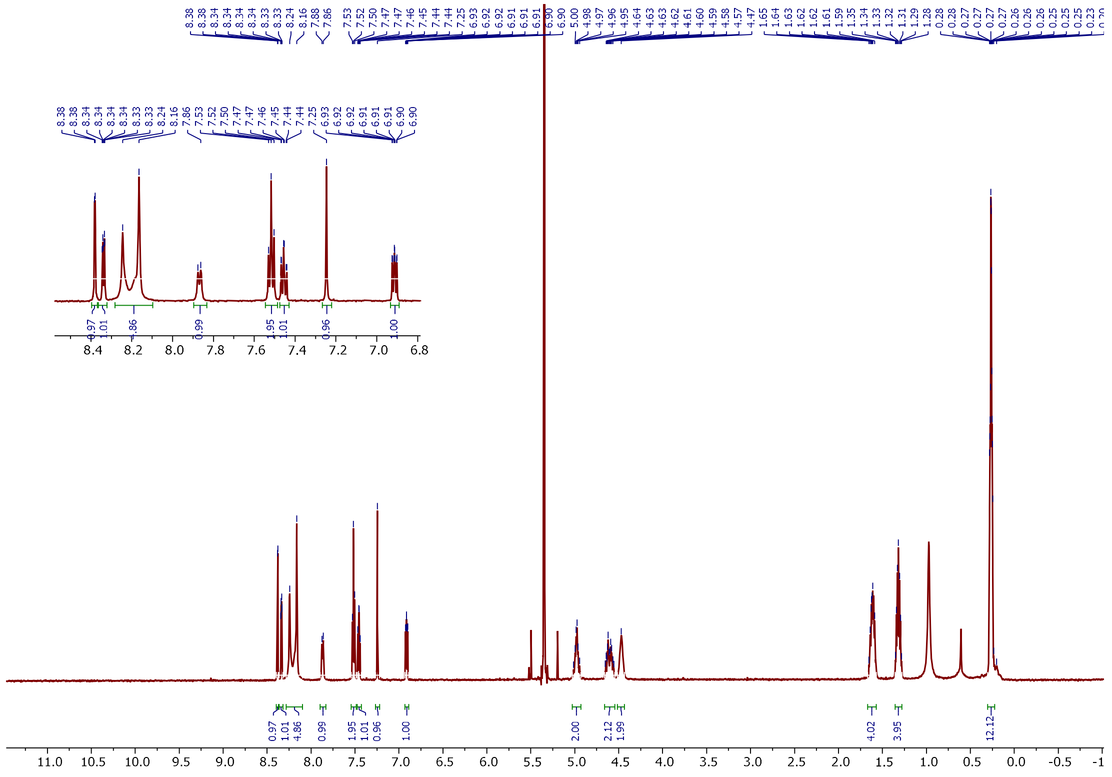

Figure S20. ^1H NMR spectrum of Re(bpy-C2-NPDI-NO_2) (600 MHz, TCE-d$_2$)

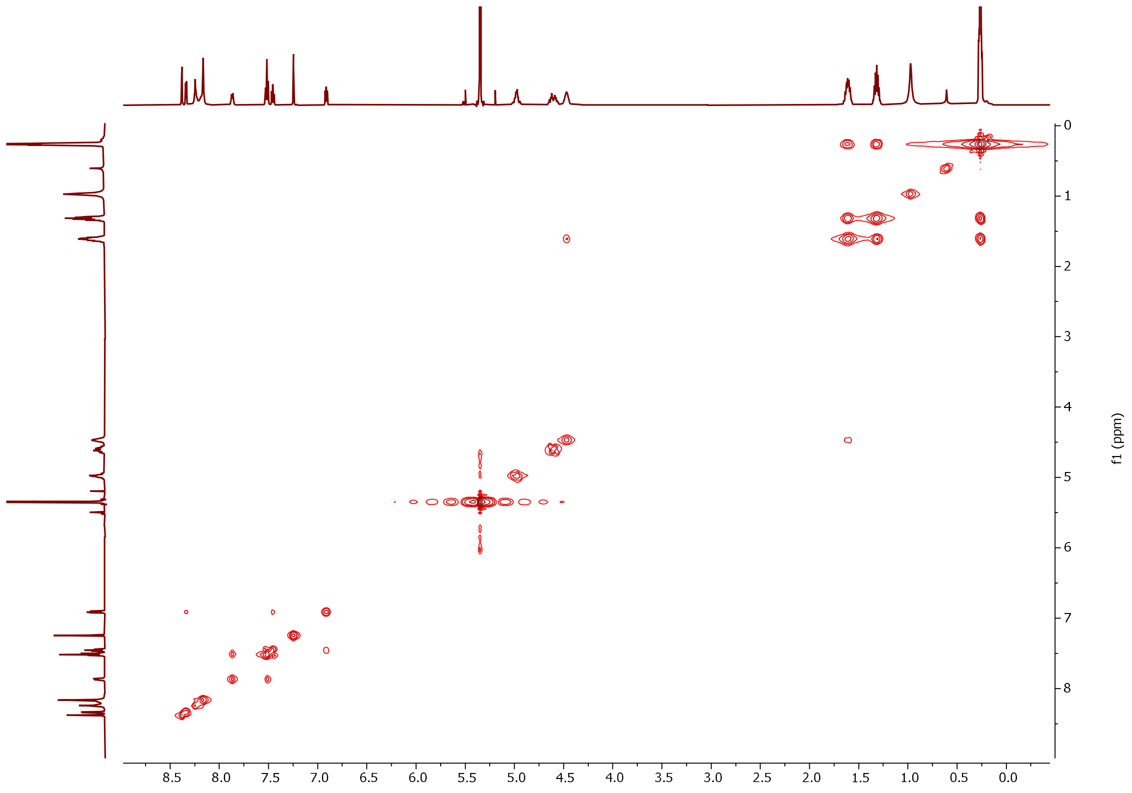

Figure S21. ^{1}H-^{1}H COSY spectrum of Re(bpy-C2-NPDI-NO_2) (600 MHz, TCE-d_2)

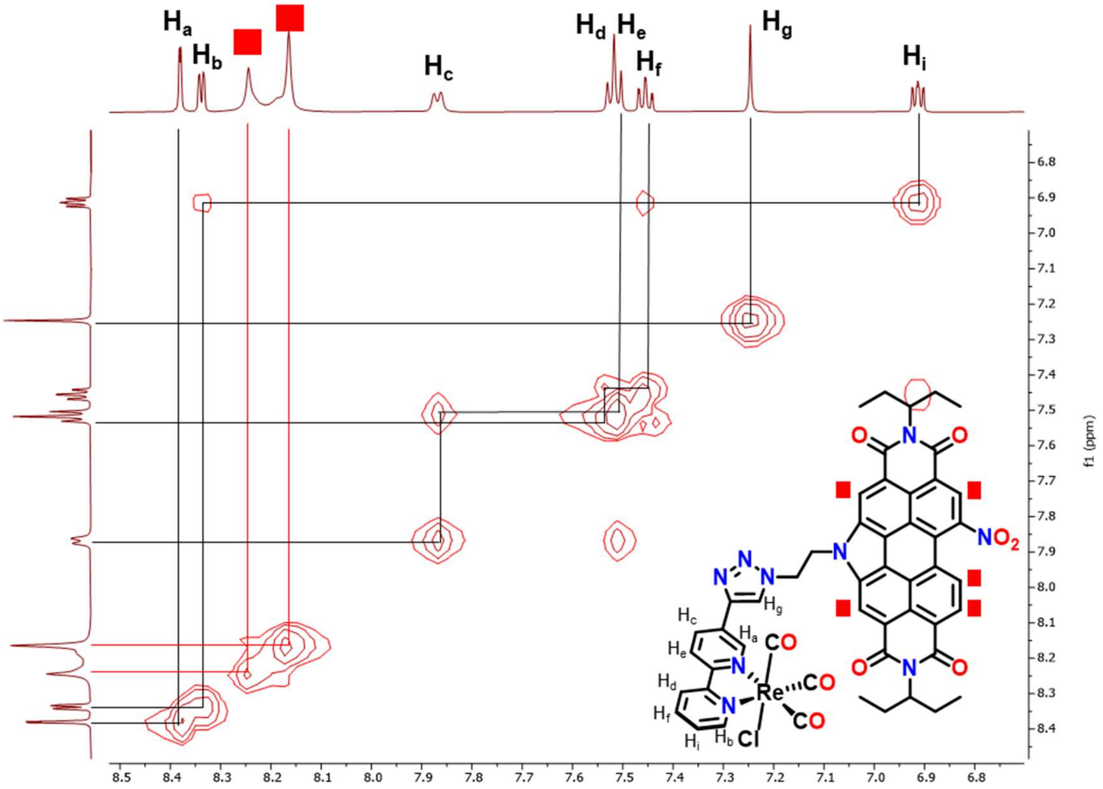

Figure S22. Enhanced view and assignment of aromatic protons in the ^{1}H-^{1}H COSY spectrum of Re(bpy-C2-NPDI-NO_2) (600 MHz, TCE-d_2)

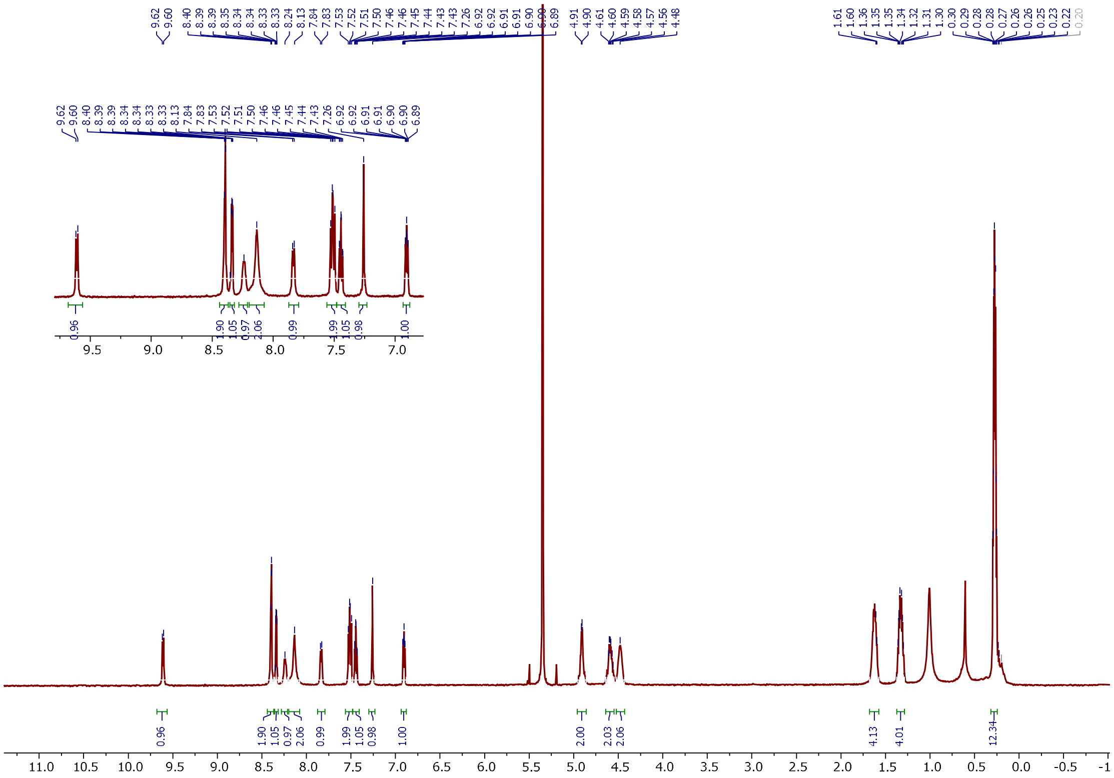

Figure S23. ^{1}H NMR spectrum of Re(bpy-C2-NPDI-Br) (600 MHz, TCE-d_2)

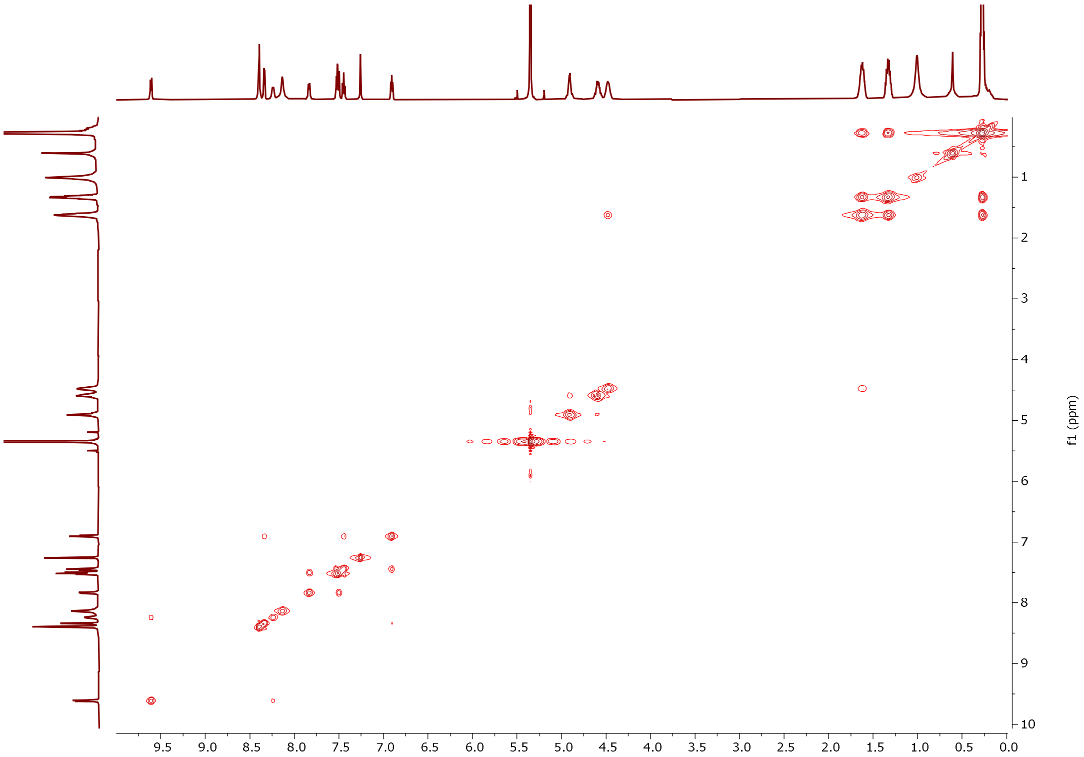

Figure S24. ^{1}H-^{1}H COSY spectrum of Re(bpy-C2-NPDI-Br) (600 MHz, TCE-d_2)

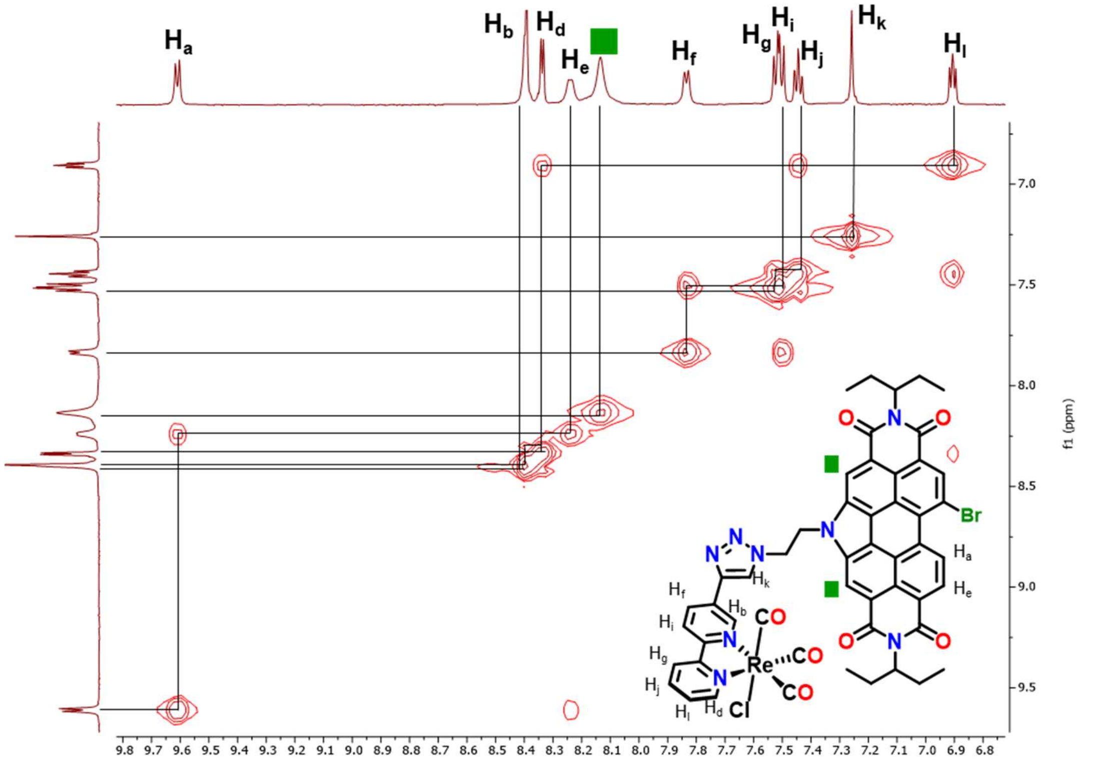

Figure S25. Enhanced view and assignment of aromatic protons in the ^{1}H-^{1}H COSY spectrum of Re(bpy-C2-NPDI-Br) (600 MHz, TCE-d_2)

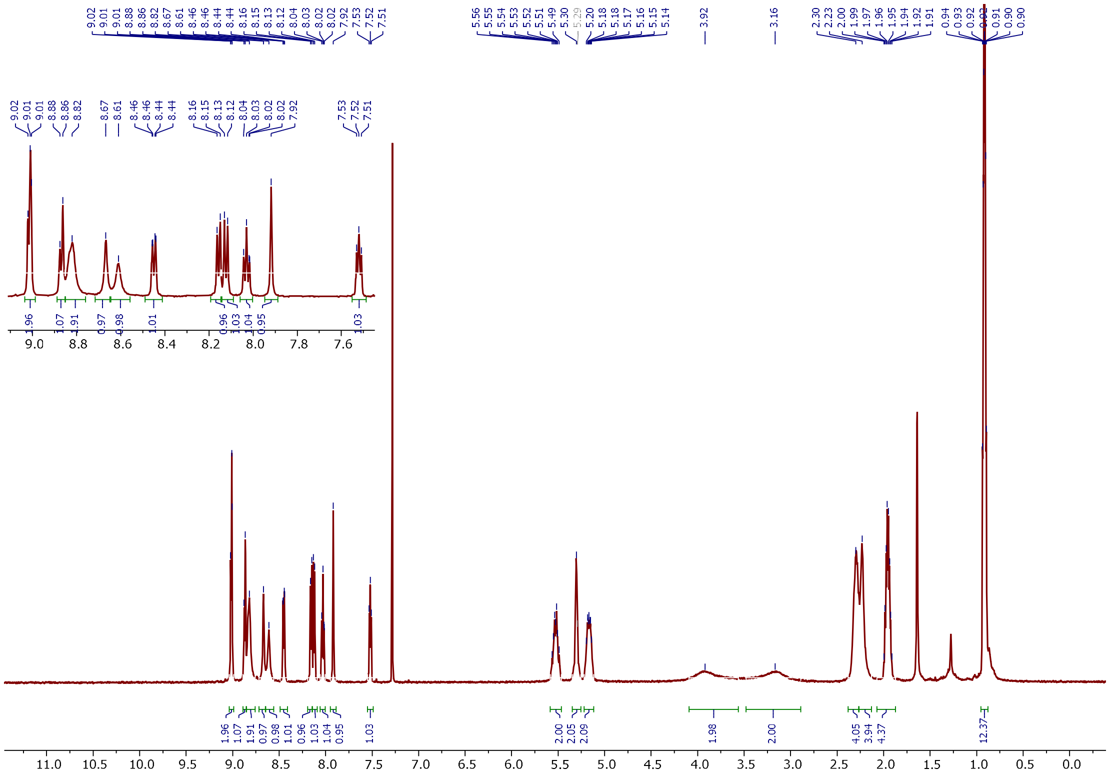

Figure S26. ^{1}H NMR spectrum of Re(bpy-C2-NPDI-NR_2) (600 MHz, $CDCl_3$)

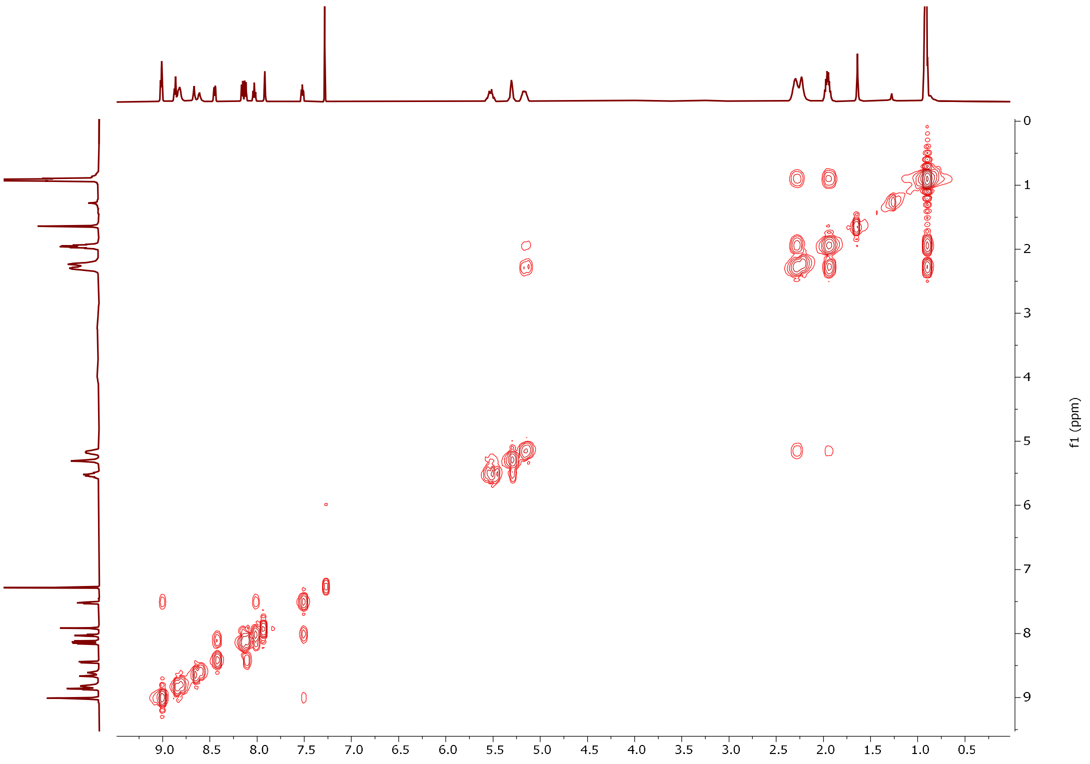

Figure S27. ^1H-^1H COSY spectrum of Re(bpy-C2-NPDI-NR$_2$) (600 MHz, $CDCl_3$)

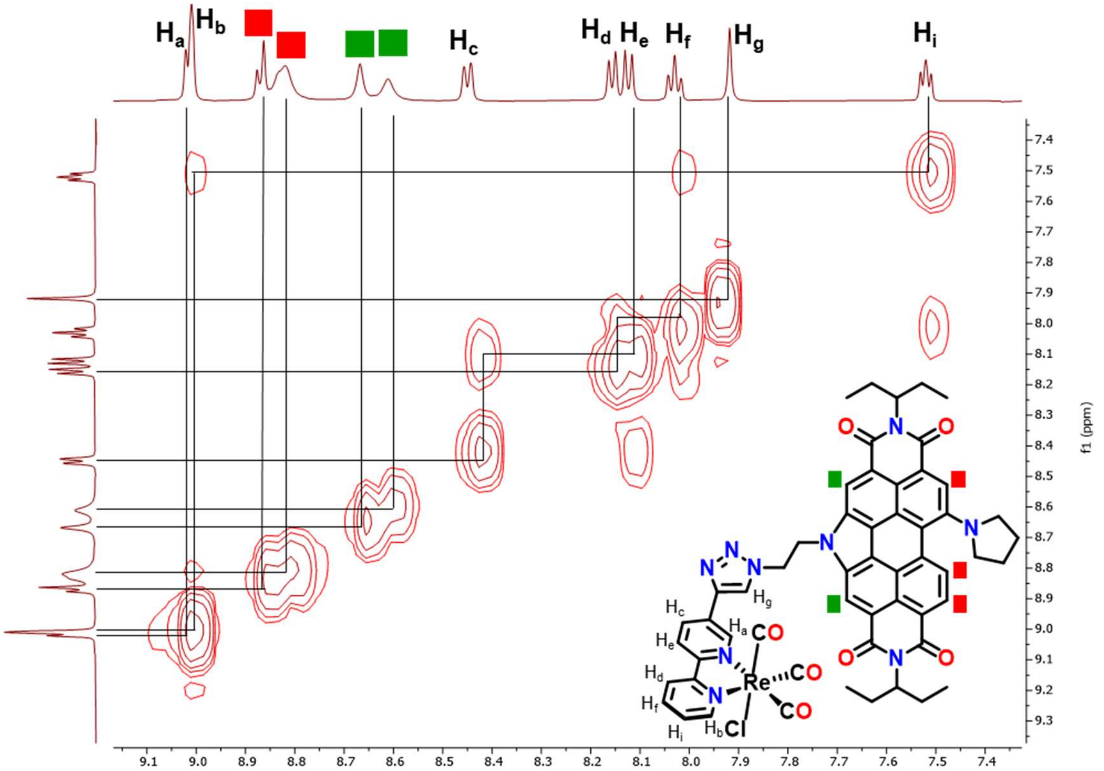

Figure S28. Enhanced view and assignment of aromatic protons in the ^1H-^1H COSY spectrum of Re(bpy-C2-NPDI-NR$_2$) (600 MHz, $CDCl_3$)

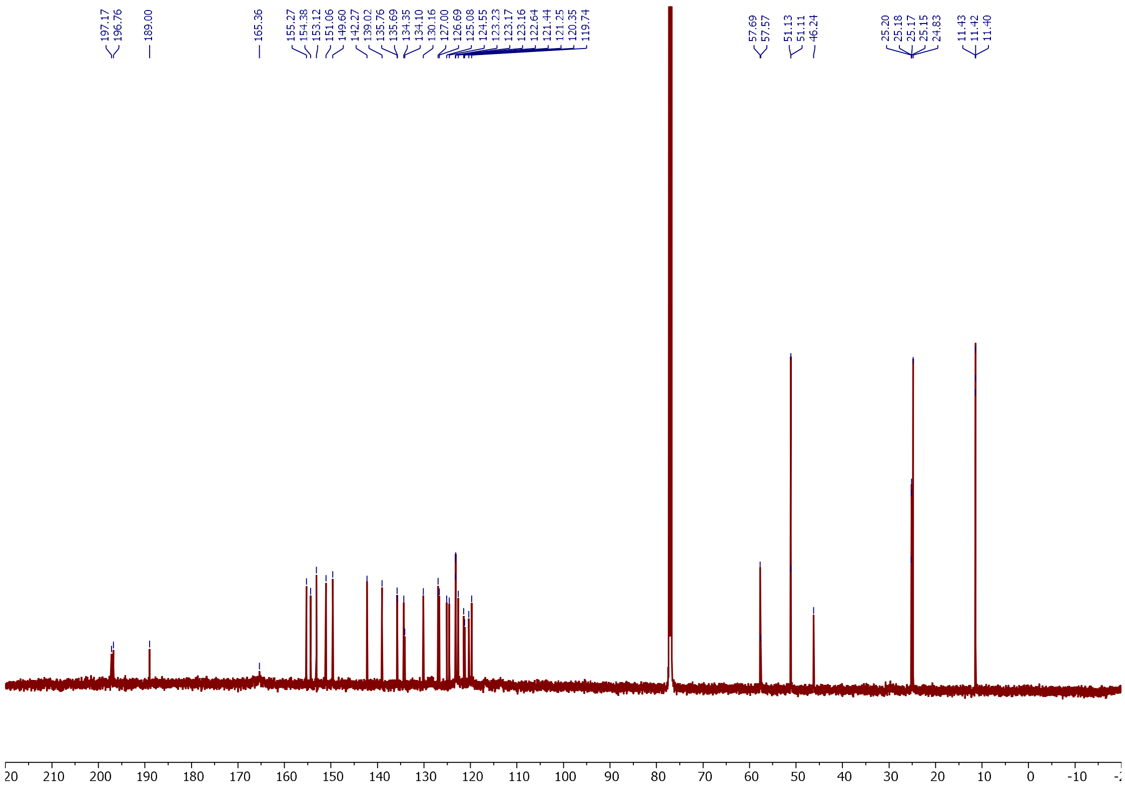

Figure S29. $^{13}C\{^{1}H\}$ NMR spectrum of Re(bpy-C2-NPDI-NR$_2$) (151 MHz, $CDCl_3$)

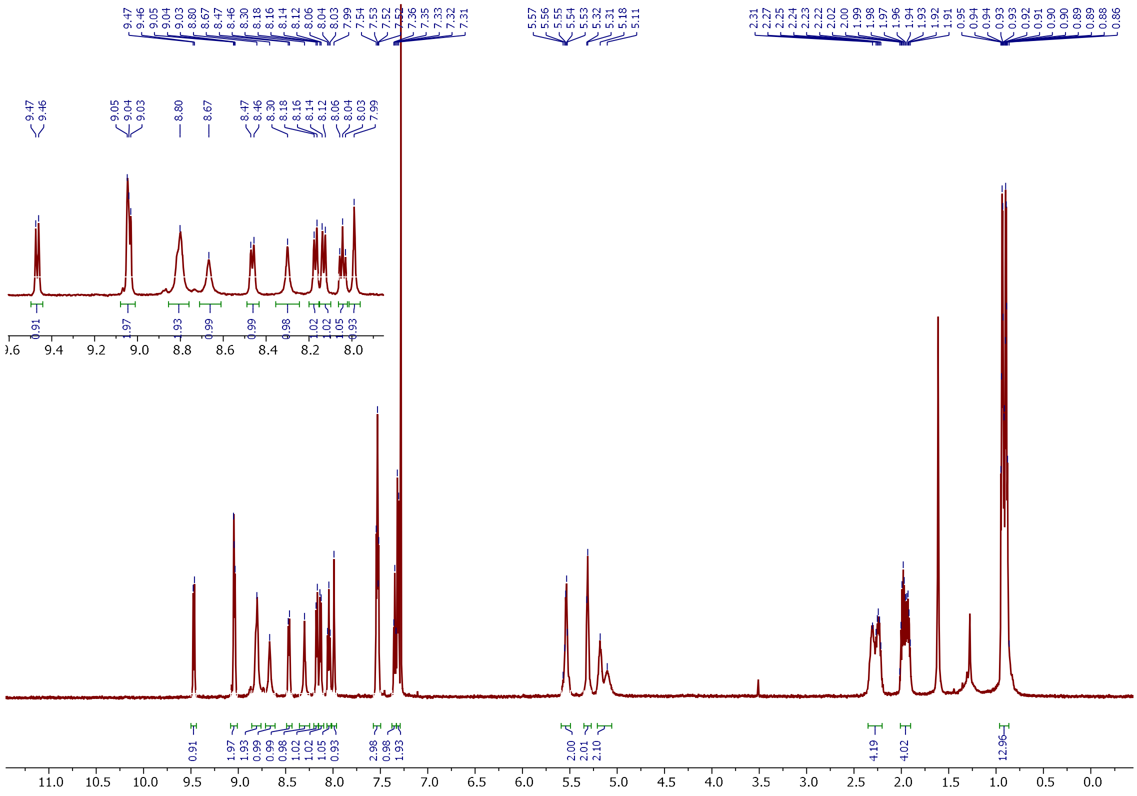

Figure S30. ^{1}H NMR spectrum of Re(bpy-C2-NPDI-OPh) (600 MHz, $CDCl_3$)

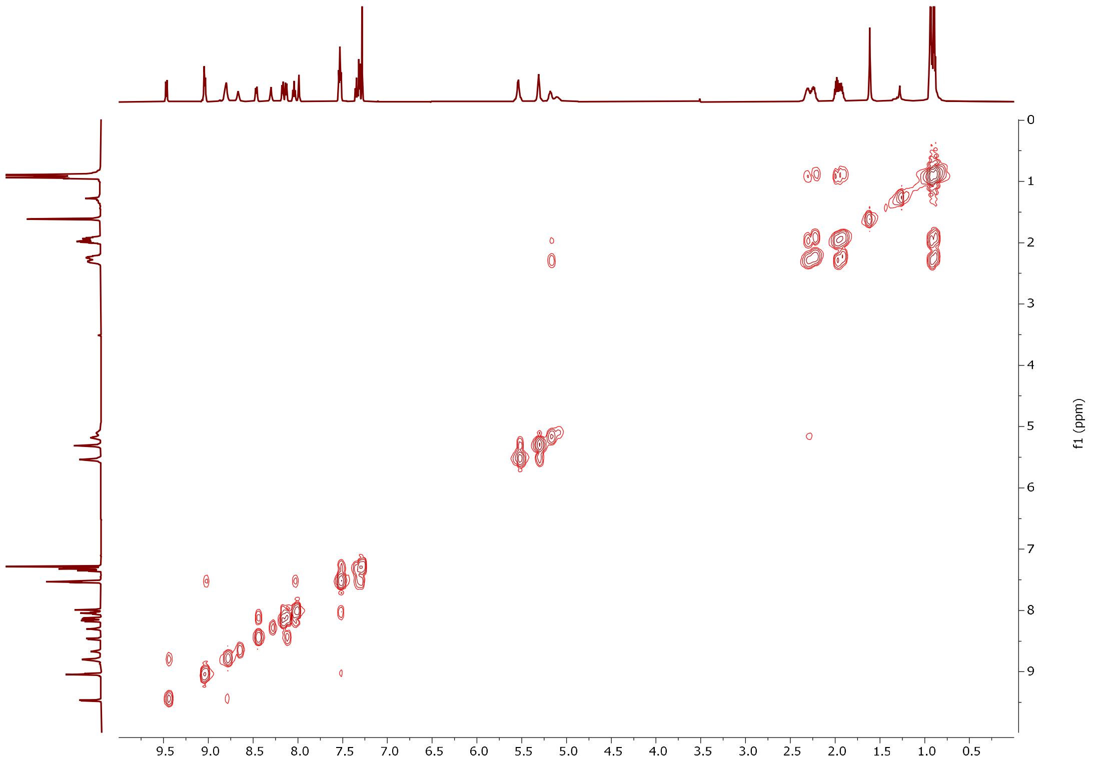

Figure S31. ^{1}H-^{1}H COSY spectrum of Re(bpy-C2-NPDI-OPh) (600 MHz, $CDCl_3$)

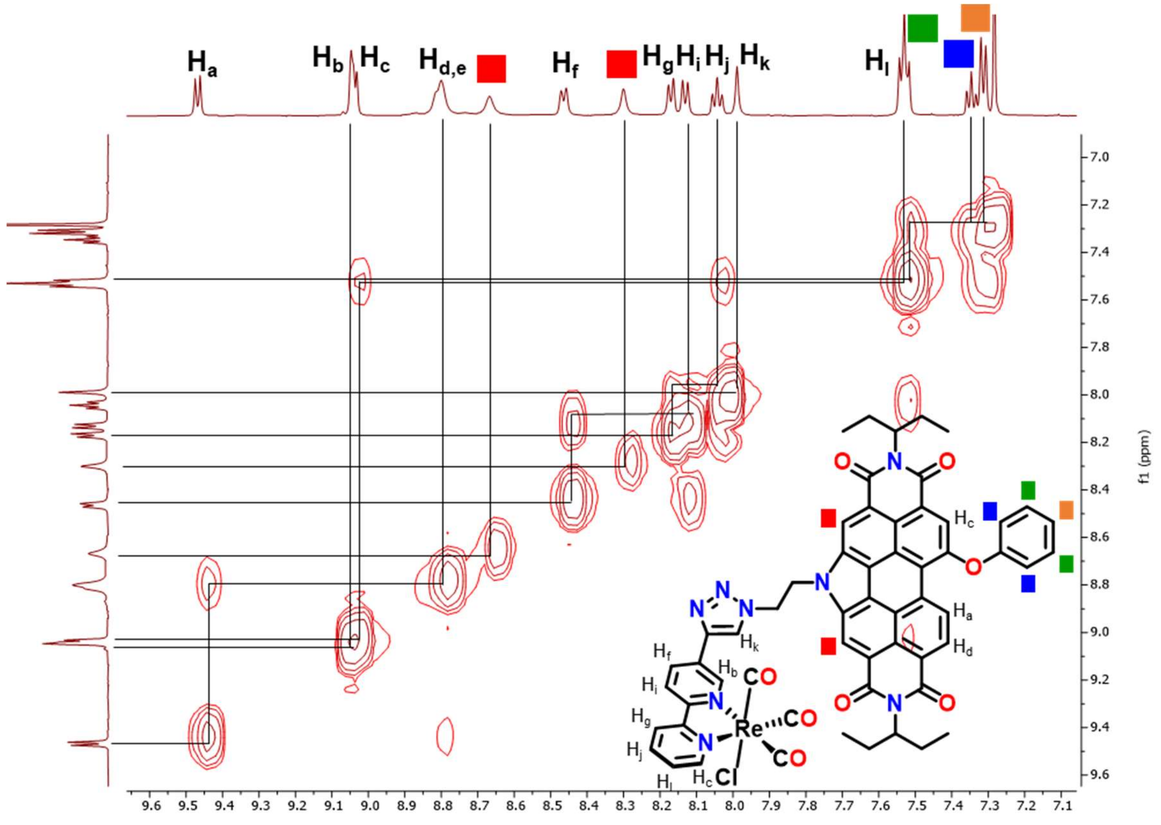

Figure S32. Enhanced view and assignment of aromatic protons in the ^{1}H-^{1}H COSY spectrum of Re(bpy-C2-NPDI-OPh) (600 MHz, $CDCl_3$)

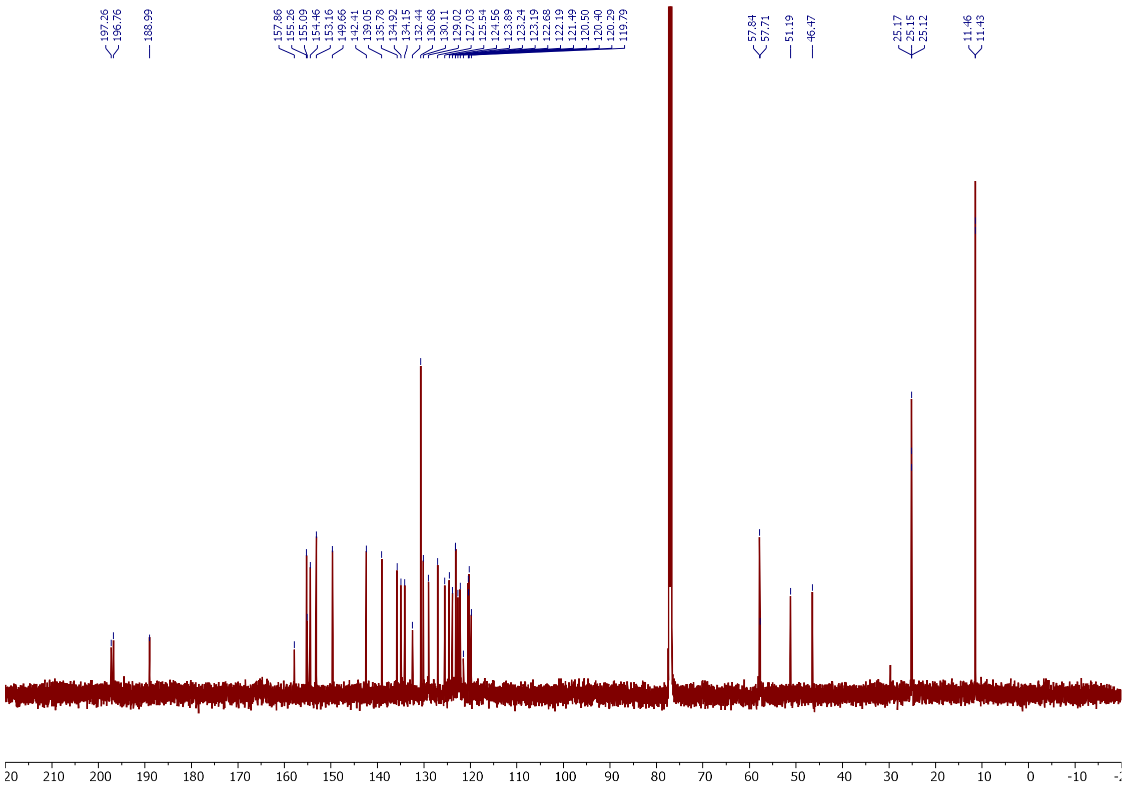

Figure S33. $^{13}C\{^{1}H\}$ NMR spectrum of Re(bpy-C2-NPDI-OPh) (151 MHz, $CDCl_3$)

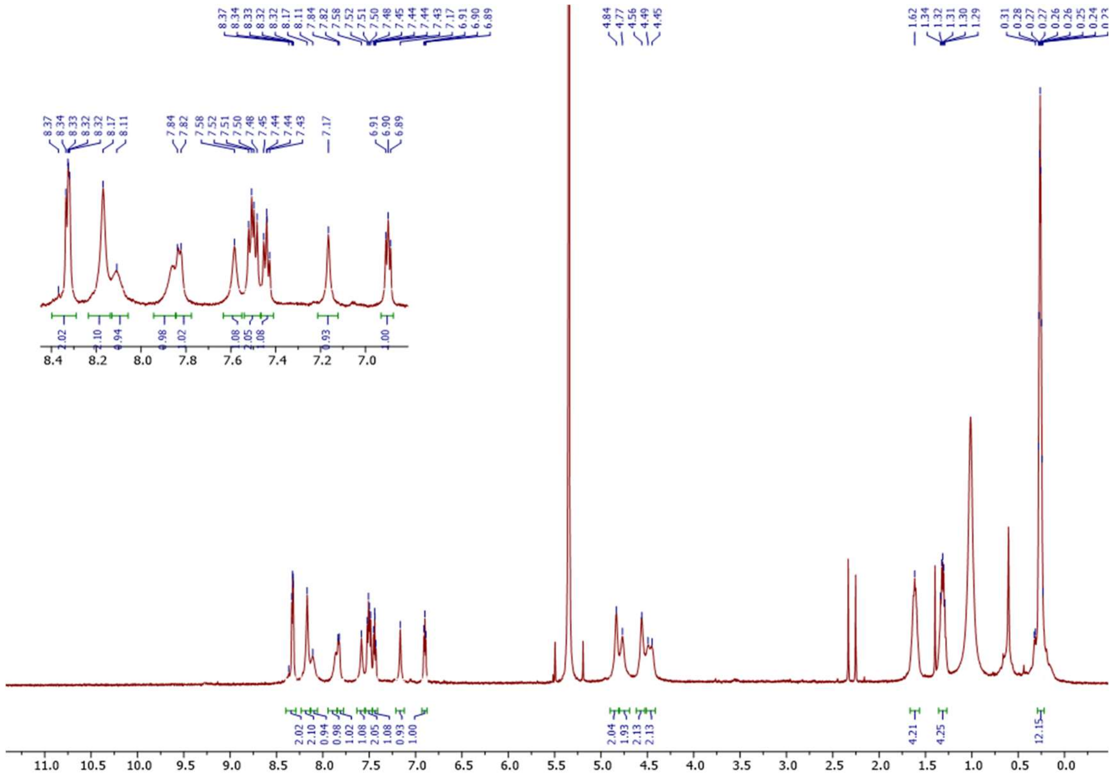

Figure S34. ^{1}H NMR spectrum of Re(bpy-C2-NPDI-NH_2) (600 MHz, TCE-d_2)

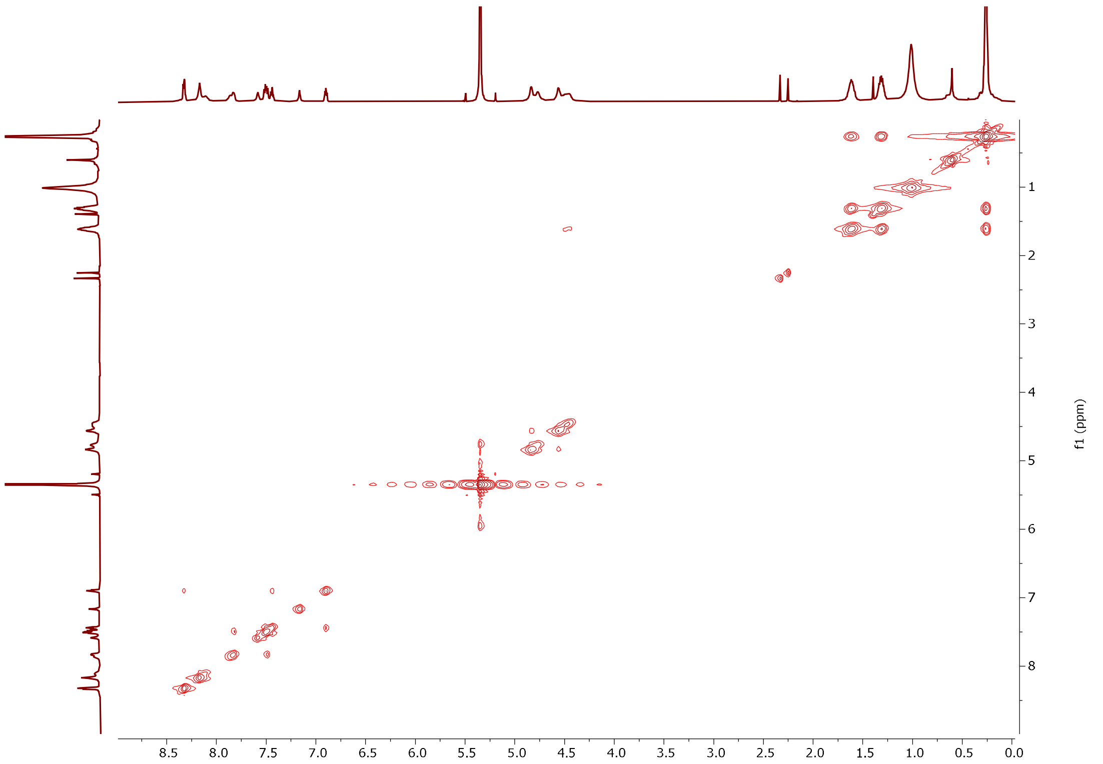

Figure S35. ^{1}H-^{1}H COSY spectrum of Re(bpy-C2-NPDI-NH_2) (600 MHz, TCE-d_2)

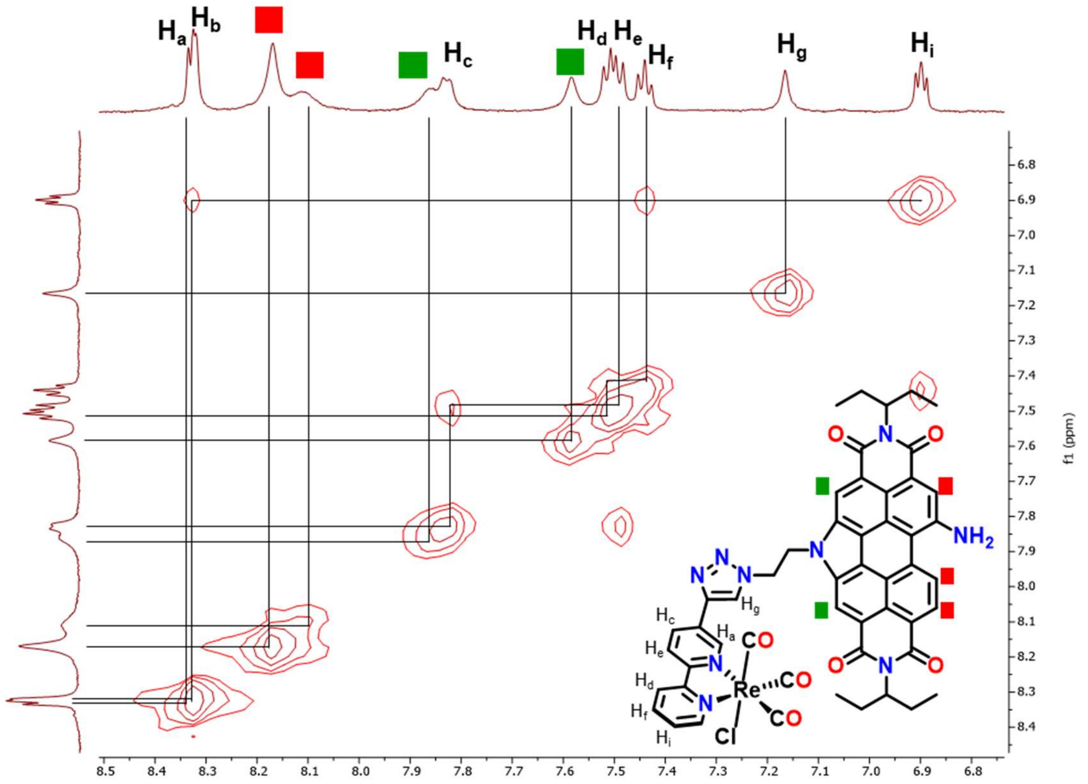

Figure S36. Enhanced view and assignment of aromatic protons in the ^{1}H-^{1}H COSY spectrum of Re(bpy-C2-NPDI-NH_2) (600 MHz, TCE-d_2)

4. MALDI-TOF MS & CHN Elemental Analysis

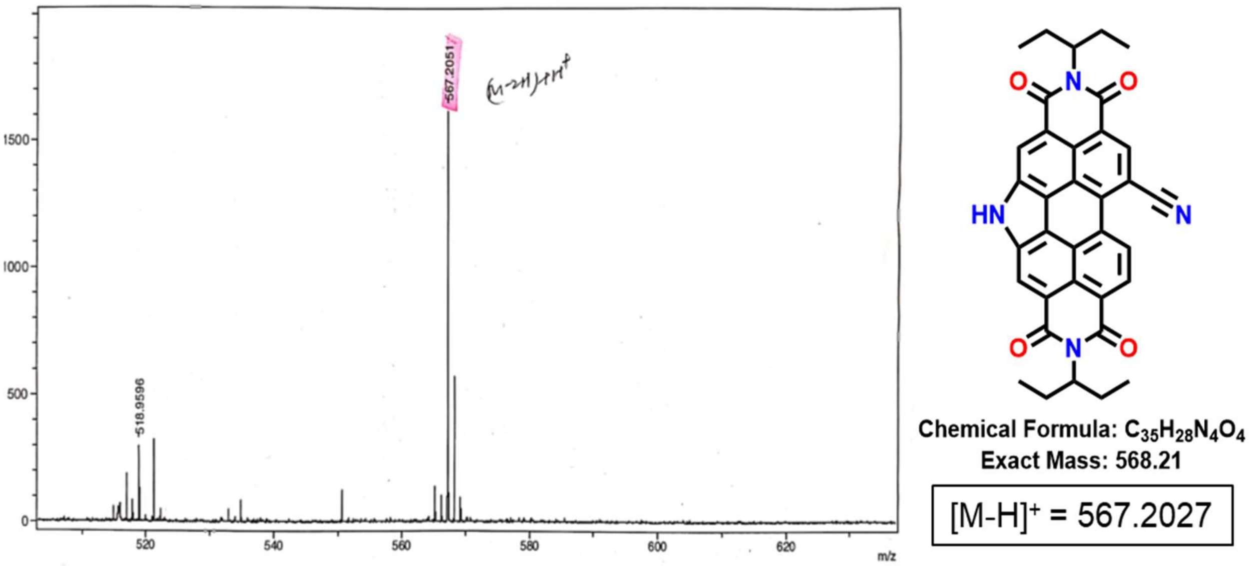

Figure S37. HR-MALDI-TOF mass spectrum of HNPDI-CN

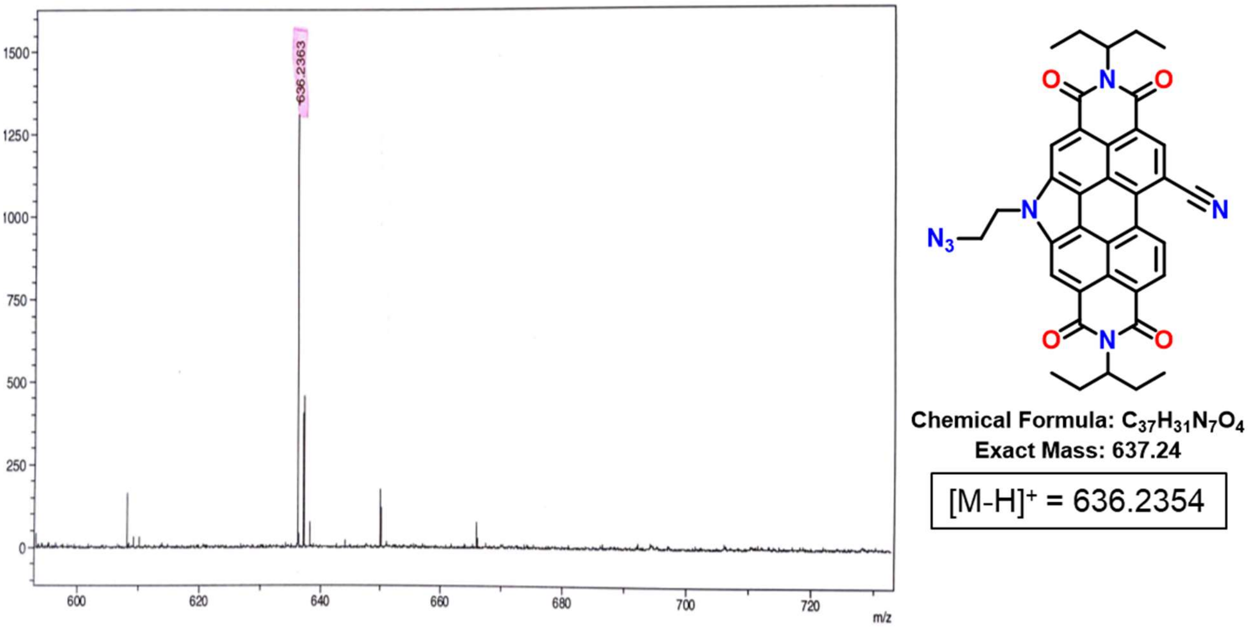

Figure S38. HR-MALDI-TOF mass spectrum of N_3-C2-NPDI-CN

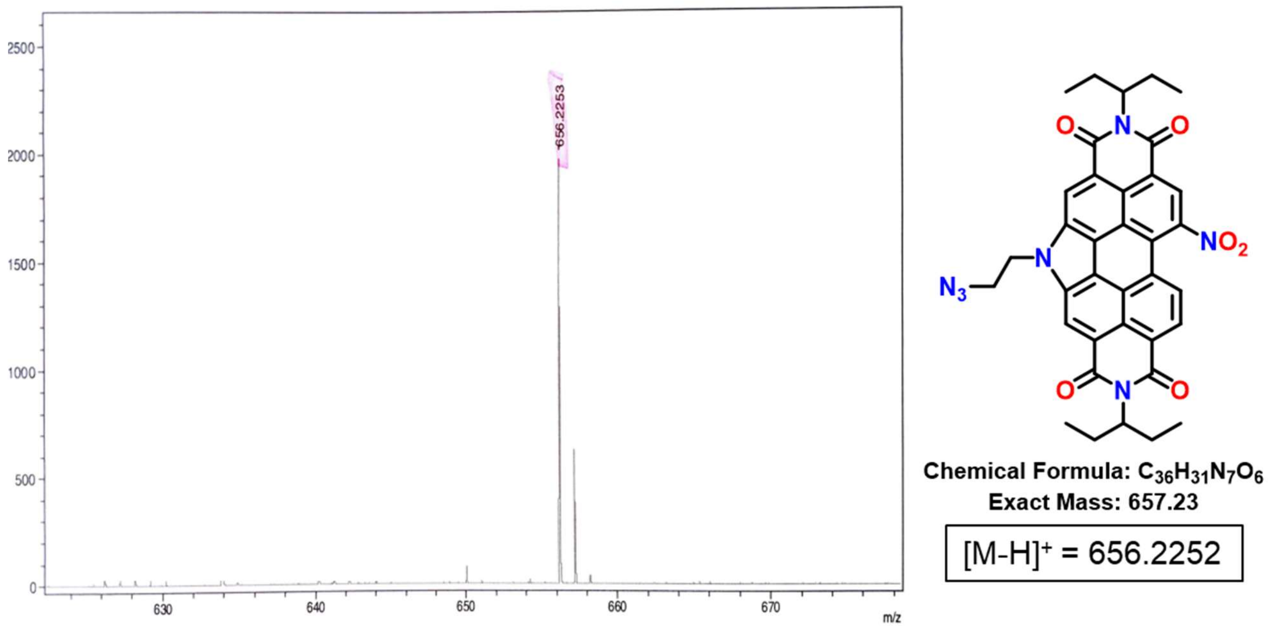

Figure S39. HR-MALDI-TOF mass spectrum of N_3-C2-NPDI-NO_2

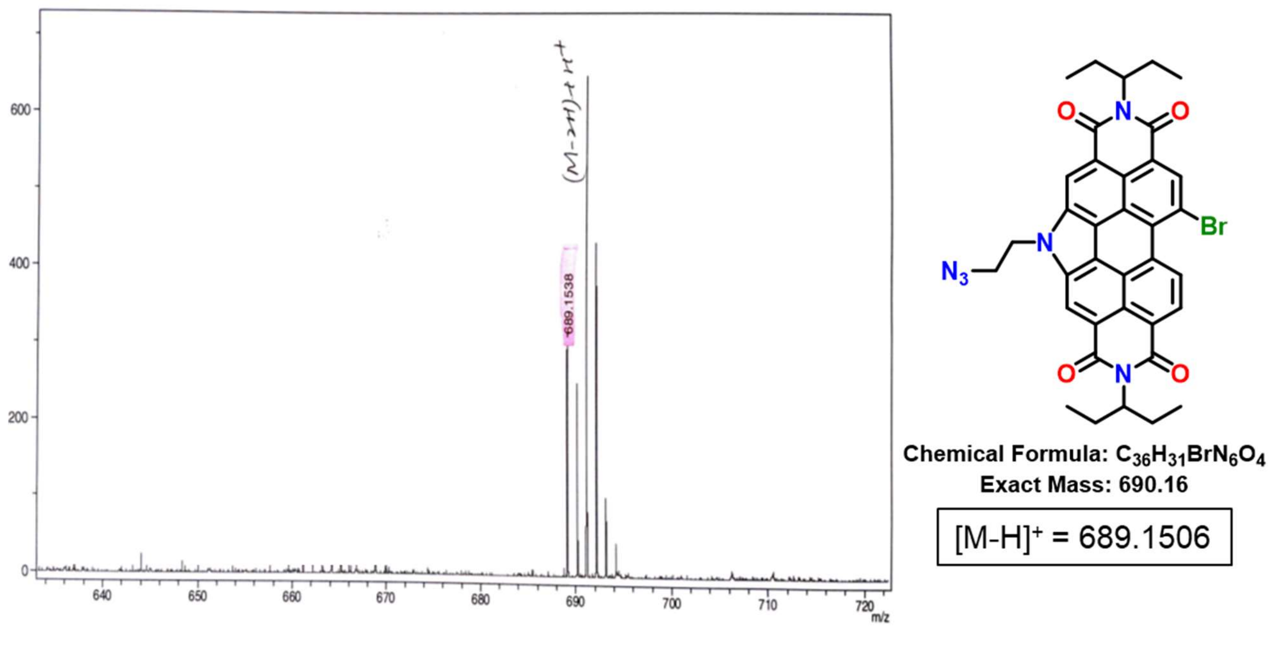

Figure S40. HR-MALDI-TOF mass spectrum of N_3-C2-NPDI-Br

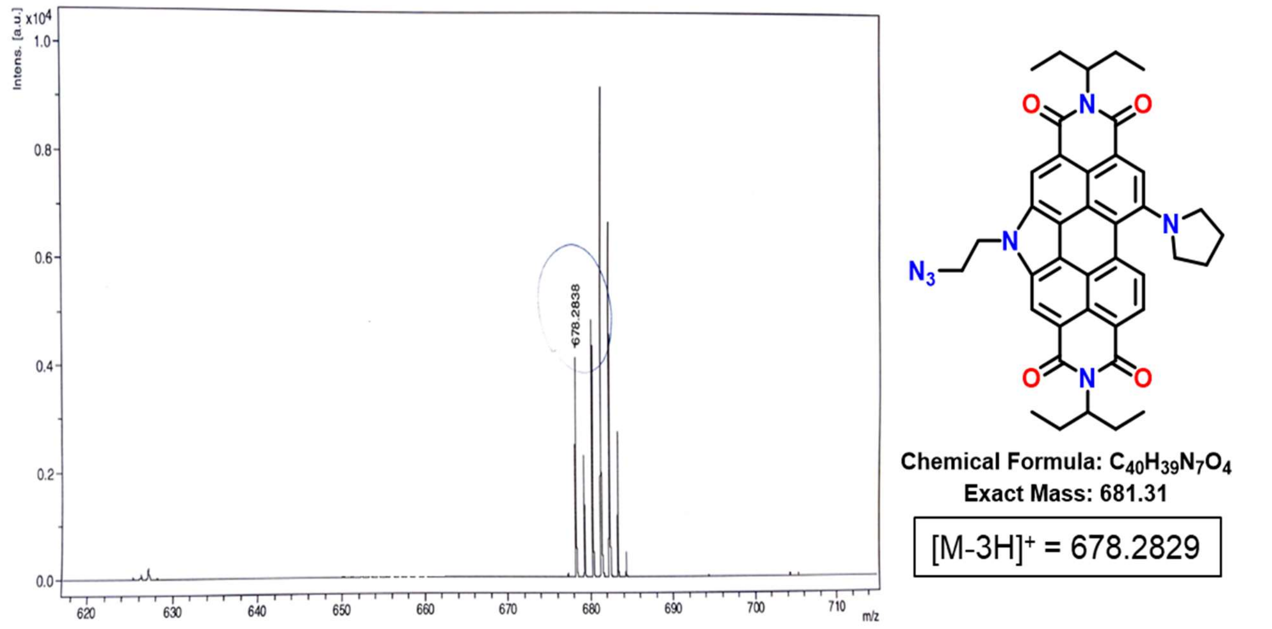

Figure S41. HR-MALDI-TOF mass spectrum of N_3-C2-NPDI-NR$_2$

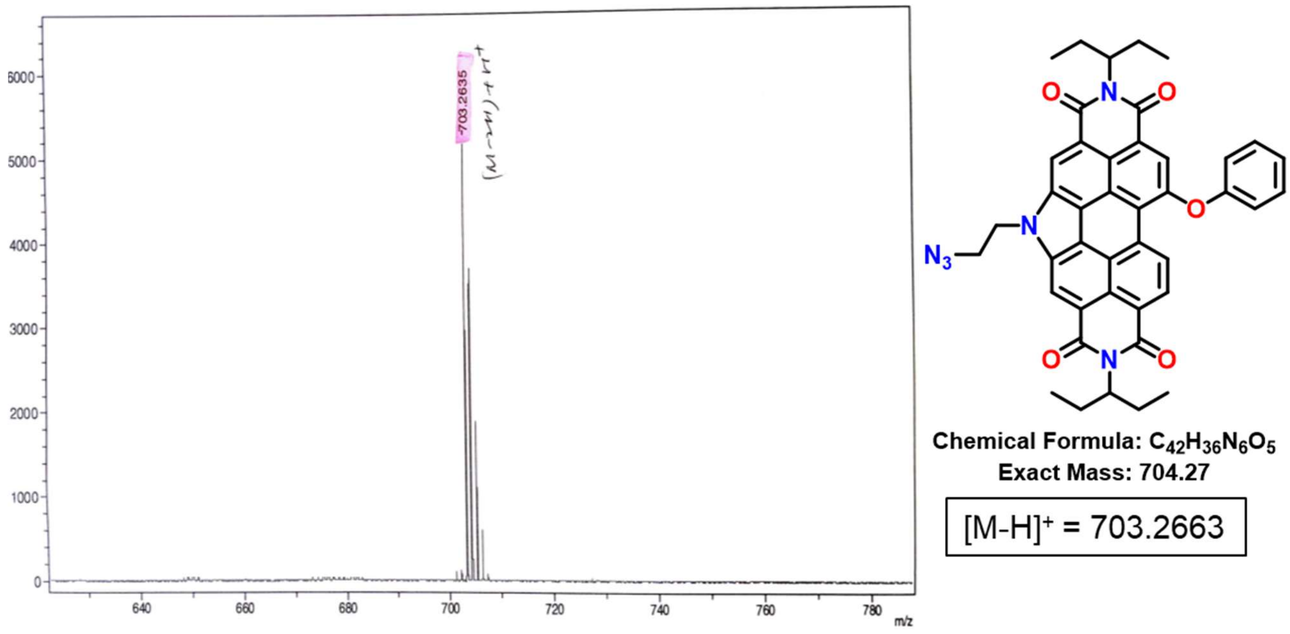

Figure S42. HR-MALDI-TOF mass spectrum of N_3-C2-NPDI-OPh

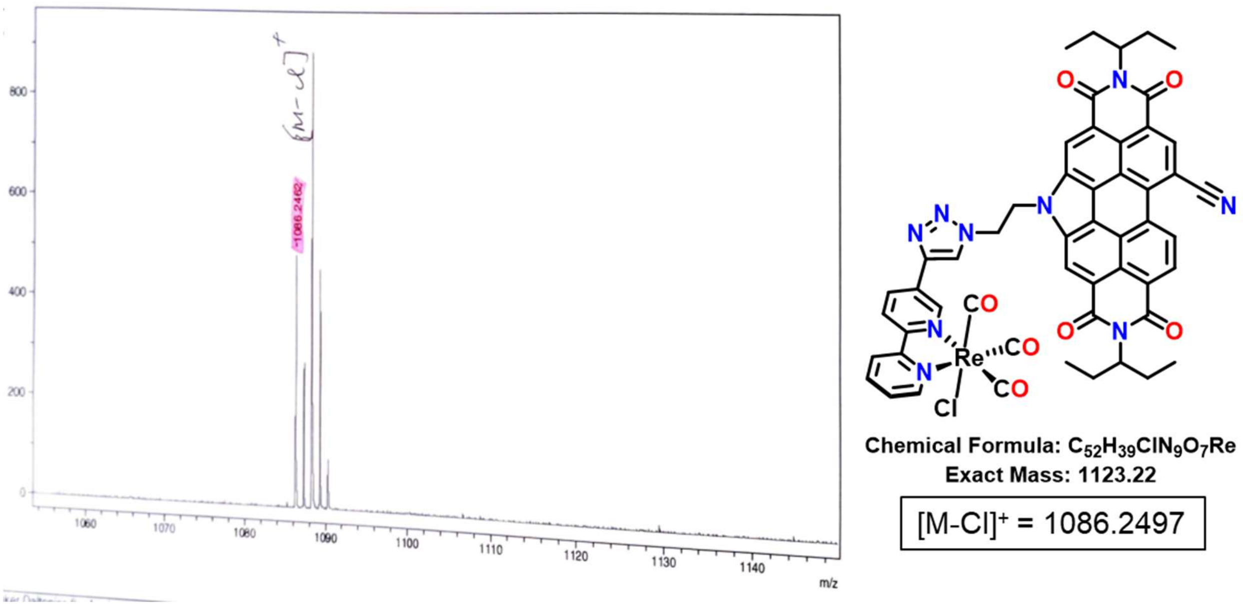

Figure S43. HR-MALDI-TOF mass spectrum of Re(bpy-C2-NPDI-CN)

University of Calgary
Department of Chemistry EA Date: 5/20/2021

Name: JOSH Group: GW

Sample: **Re(bpy-C2-NPDI-CN)** Weight (mg): 1.546

%C (Actual):	55.38	%C (Theorectical):	55.59
%H (Actual):	3.84	%H (Theoretical):	3.50
%N (Actual):	10.55	%N (Theoretical):	11.22

Figure S44. CHN elemental analysis of Re(bpy-C2-NPDI-CN)

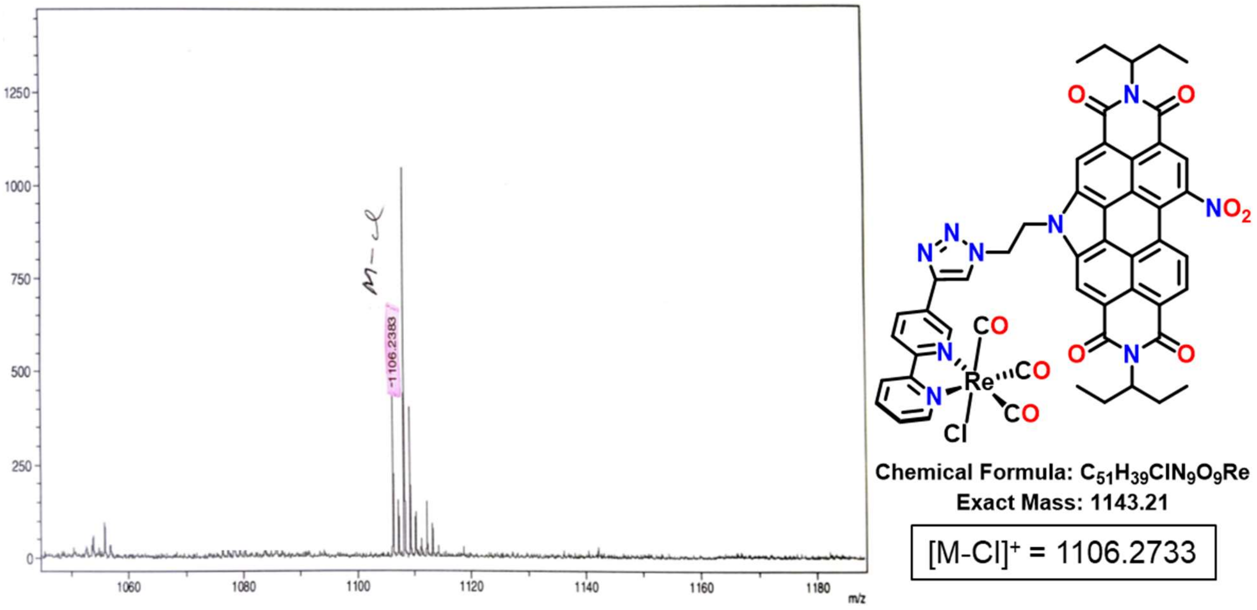

Figure S45. HR-MALDI-TOF mass spectrum of Re(bpy-C2-NPDI-NO_2)

University of Calgary
Department of Chemistry EA Date: 5/20/2021

Name:	JOSH	Group:	GW
Sample:	Re(bpy-C2-NPDI-NO_2)	Weight (mg):	1.725
%C (Actual):	53.17	%C (Theorectical):	53.57
%H (Actual):	3.57	%H (Theoretical):	3.44
%N (Actual):	10.58	%N (Theoretical):	11.02

Figure S46. CHN elemental analysis of Re(bpy-C2-NPDI-NO_2)

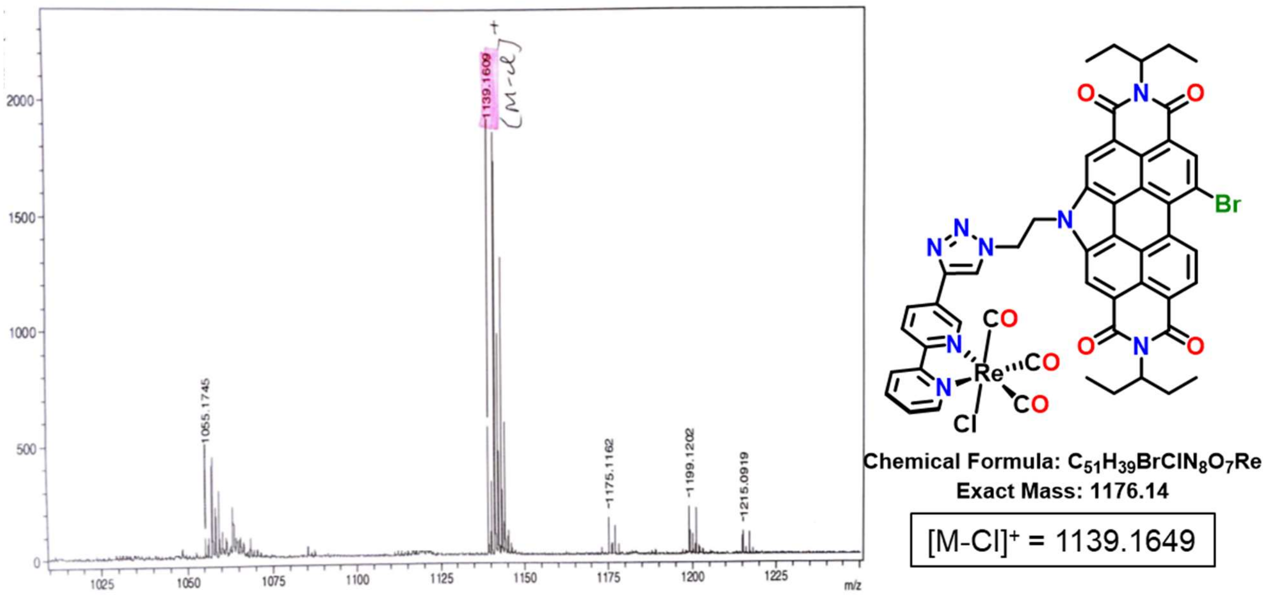

Figure S47. HR-MALDI-TOF mass spectrum of Re(bpy-C2-NPDI-Br)

University of Calgary

Department of Chemistry EA Date: 5/20/2021

Name:	JOSH	Group:	GW
Sample:	**Re(bpy-C2-NPDI-Br)**	Weight (mg):	1.9
%C (Actual):	52.38	%C (Theorectical):	52.02
%H (Actual):	3.69	%H (Theoretical):	3.34
%N (Actual):	8.99	%N (Theoretical):	9.52

Figure S48. CHN elemental analysis of Re(bpy-C2-NPDI-Br)

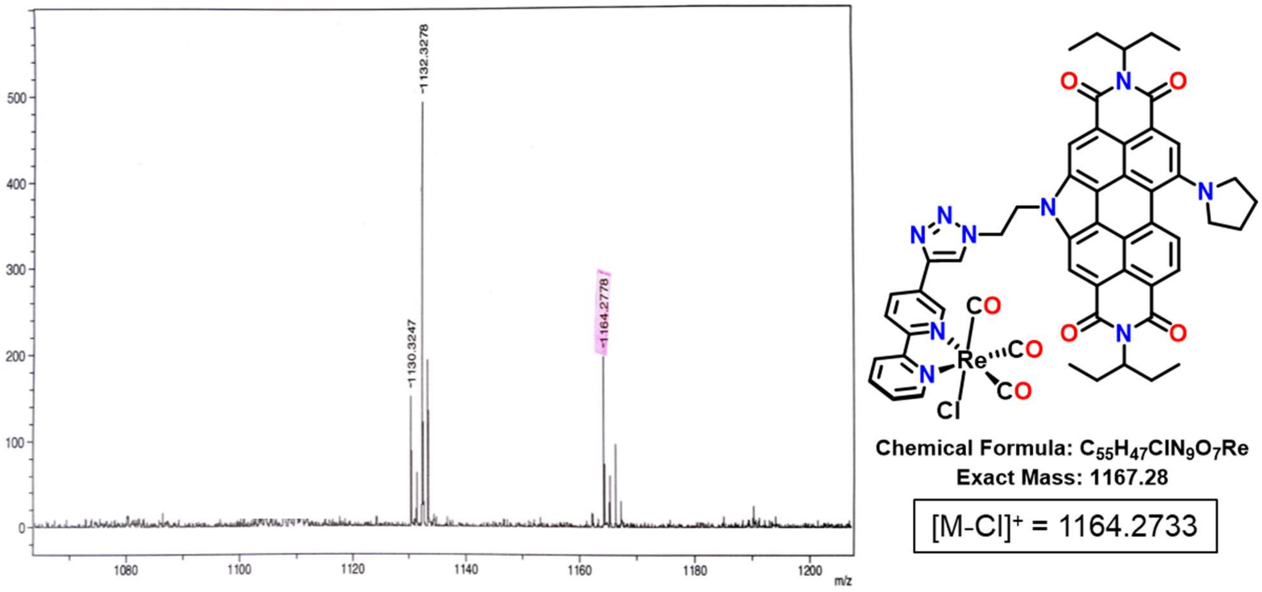

Figure S49. HR-MALDI-TOF mass spectrum of Re(bpy-C2-NPDI-NR$_2$)

University of Calgary
Department of Chemistry EA Date: 5/20/2021

Name:	JOSH	Group:	GW
Sample:	**Re(bpy-C2-NPDI-NR$_2$)**	Weight (mg):	1.069
%C (Actual):	56.15	%C (Theoretical):	56.57
%H (Actual):	4.05	%H (Theoretical):	4.06
%N (Actual):	10.31	%N (Theoretical):	10.80

Figure S50. CHN elemental analysis of Re(bpy-C2-NPDI-NR$_2$)

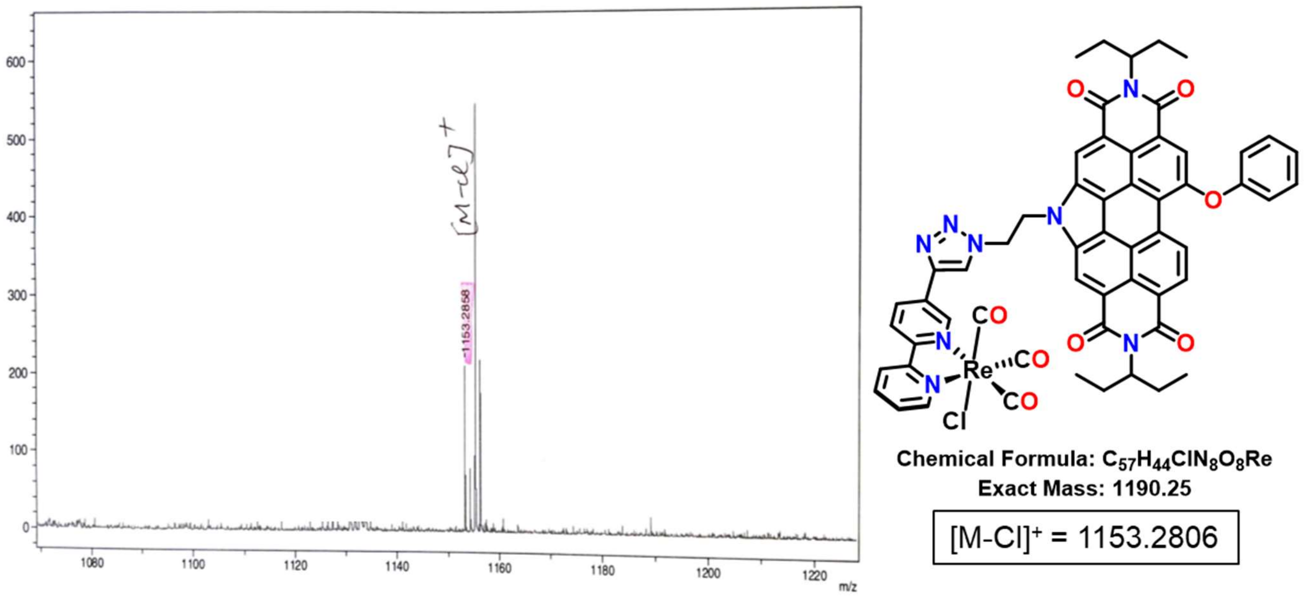

Figure S51. HR-MALDI-TOF mass spectrum of Re(bpy-C2-NPDI-OPh)

University of Calgary
Department of Chemistry EA Date: 5/20/2021

Name:	JOSH	Group:	GW
Sample:	**Re(bpy-C2-NPDI-OPh)**	Weight (mg):	1.995
%C (Actual):	56.89	%C (Theorectical):	57.50
%H (Actual):	4.07	%H (Theoretical):	3.72
%N (Actual):	9.06	%N (Theoretical):	9.41

Figure S52. CHN elemental analysis of Re(bpy-C2-NPDI-OPh)

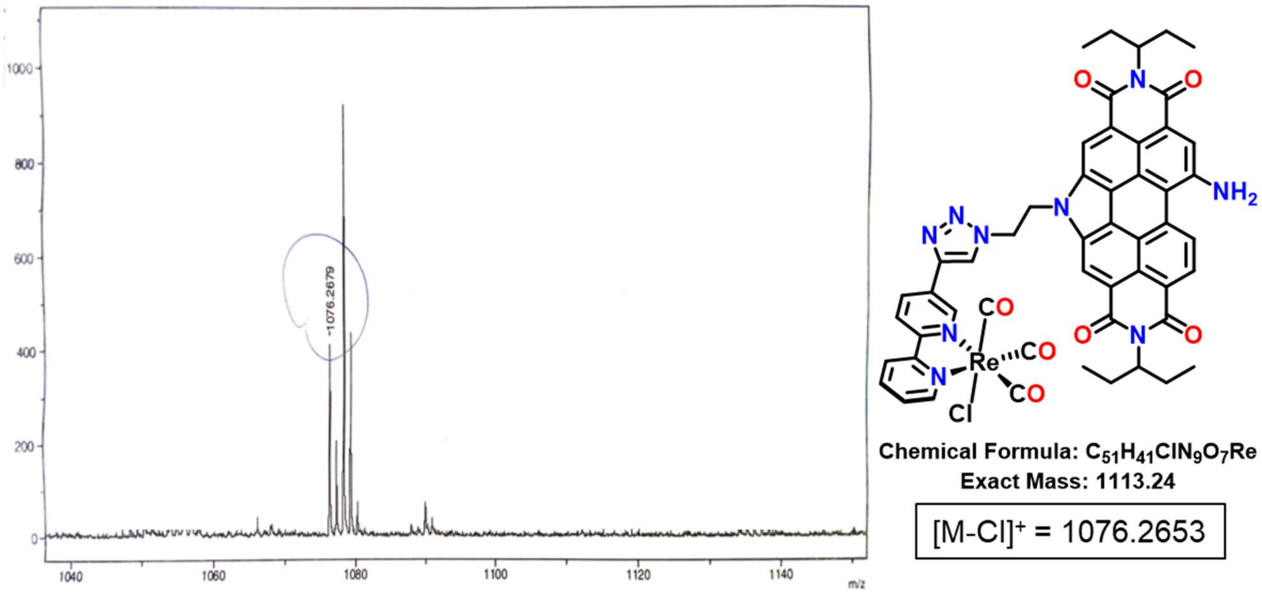

Figure S53. HR-MALDI-TOF mass spectrum of Re(bpy-C2-NPDI-NH$_2$)

University of Calgary
Department of Chemistry EA Date: 8/11/2021

Name:	JOSH	Group:	GW
Sample:	**Re(bpy-C2-NPDI-NH$_2$)**	Weight (mg):	1.309
%C (Actual):	53.87	%C (Theorectical):	55.01
%H (Actual):	3.95	%H (Theoretical):	3.71
%N (Actual):	10.35	%N (Theoretical):	11.32

Figure S54. CHN elemental analysis of Re(bpy-C2-NPDI-NH$_2$)

5. UV-Vis-nIR / FTIR Spectroscopies & Spectroelectrochemistry

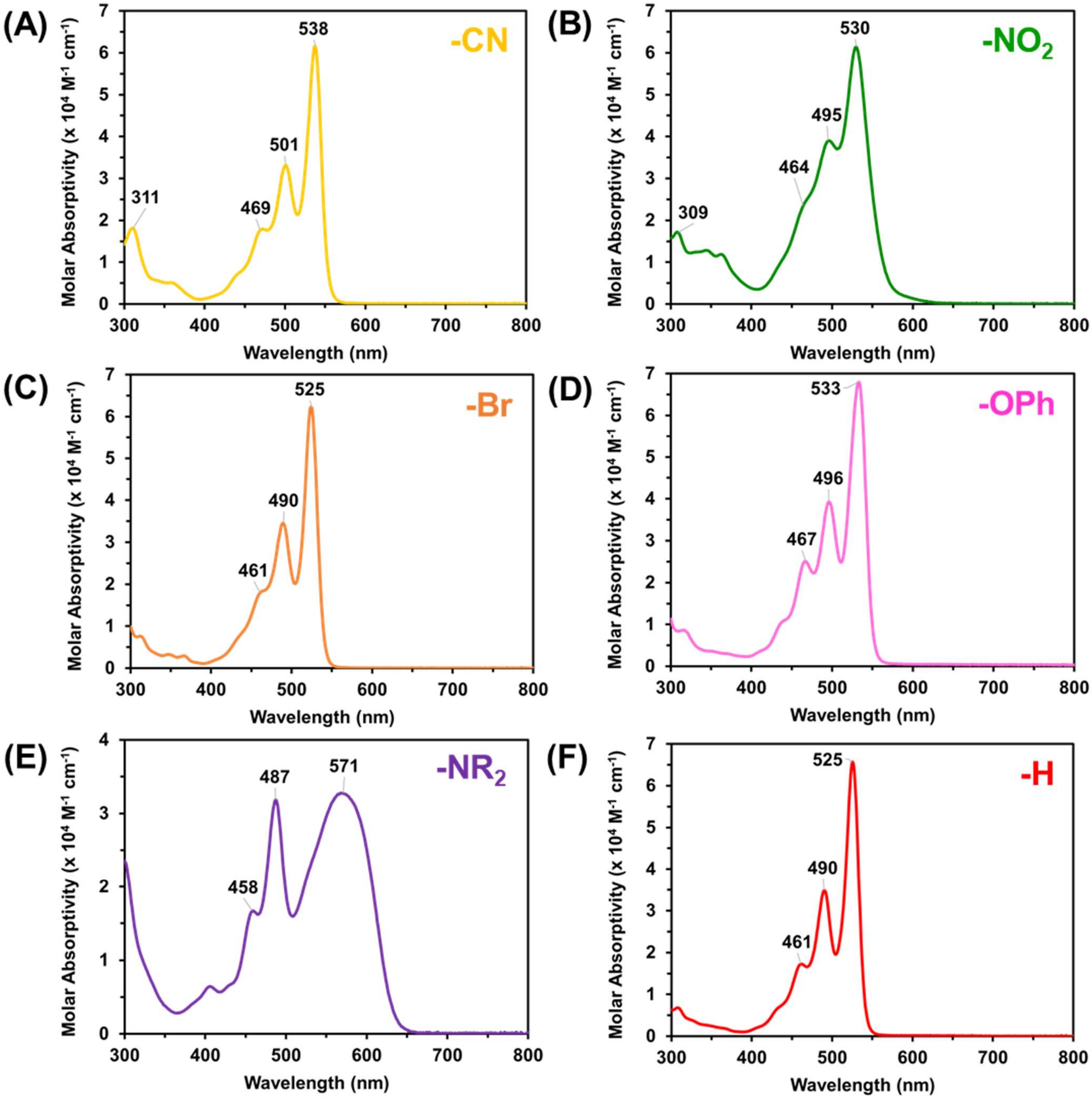

Figure S55. UV-vis absorbance spectra of (A) N_3-C2-NPDI-CN) (yellow), (B) N_3-C2-NPDI-NO_2 (green), (C) N_3-C2-NPDI-Br (orange), (D) N_3-C2-NPDI-OPh (magenta) , (E) N_3-C2-NPDI-NR_2 (purple), and (F) N_3-C2-NPDI-H (red).

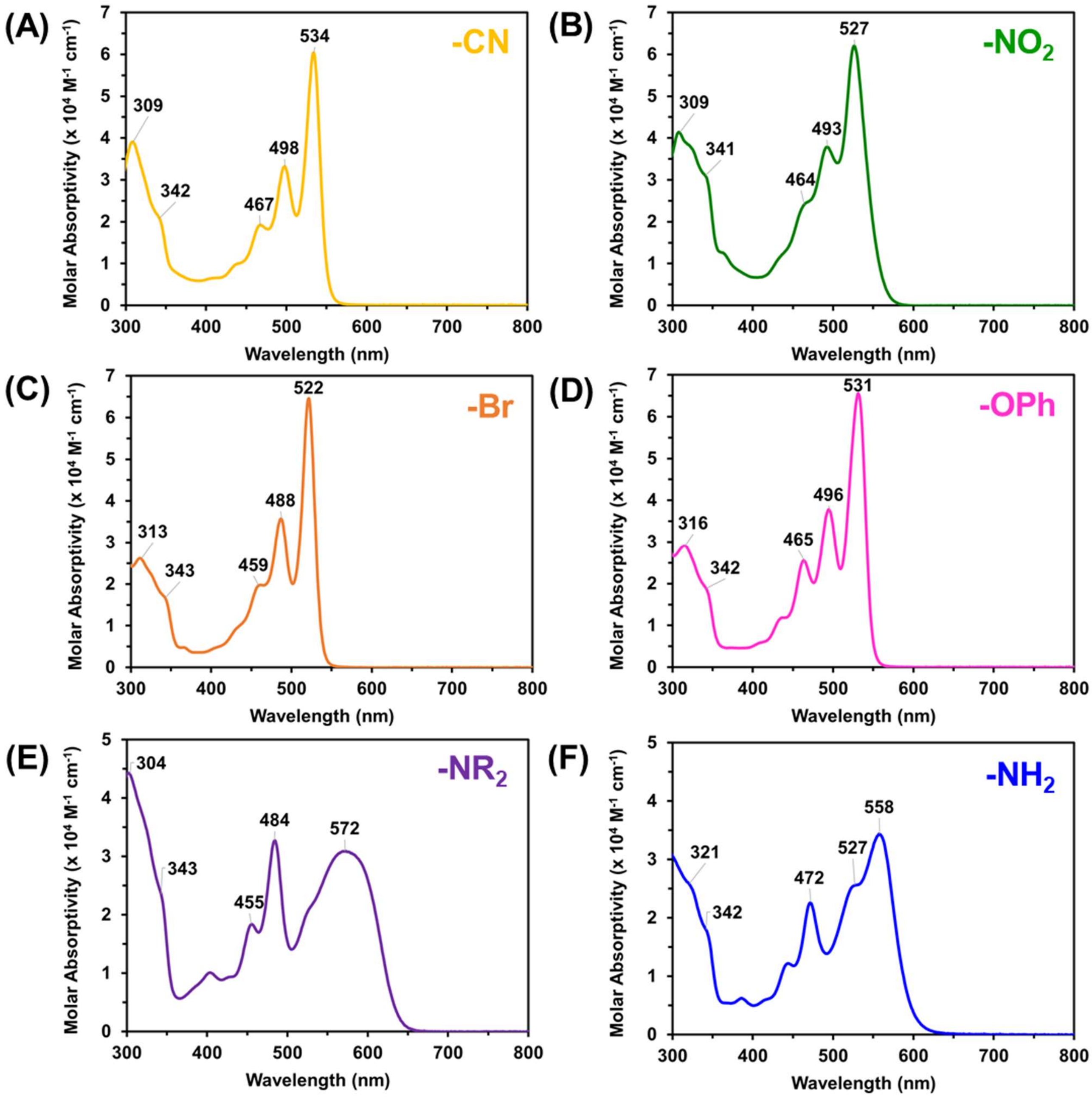

Figure S56. UV-vis absorbance spectra of (A) Re(bpy-C2-NPDI-CN) (yellow), (B) Re(bpy-C2-NPDI-NO_2) (green), (C) Re(bpy-C2-NPDI-Br) (orange), (D) Re(bpy-C2-NPDI-OPh) (magenta) , (E) Re(bpy-C2-NPDI-NR_2) (purple), and (F) Re(bpy-C2-NPDI-NH_2) (blue).

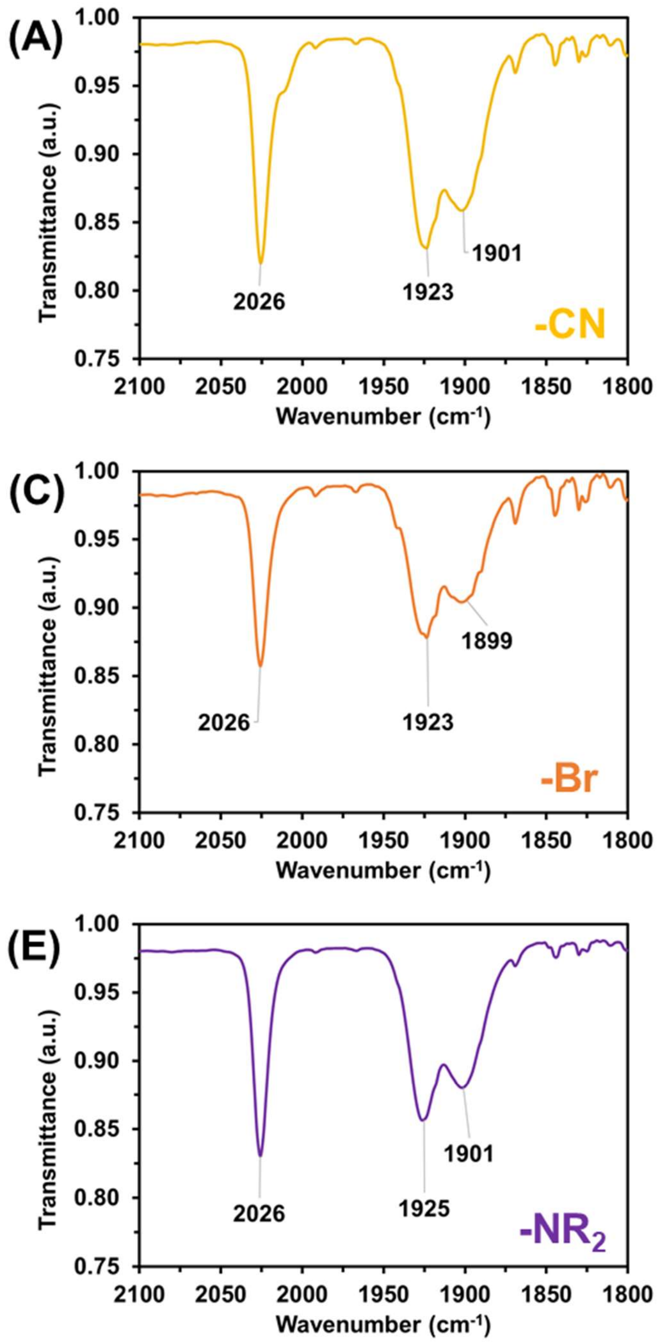

Figure S57. Fourier transform infrared spectra of (A) Re(bpy-C2-NPDI-CN) (yellow), (B) Re(bpy-C2-NPDI-NO_2) (green), (C) Re(bpy-C2-NPDI-Br) (orange), (D) Re(bpy-C2-NPDI-OPh) (magenta) , (E) Re(bpy-C2-NPDI-NR_2) (purple), and (F) Re(bpy-C2-NPDI-NH_2) (blue).

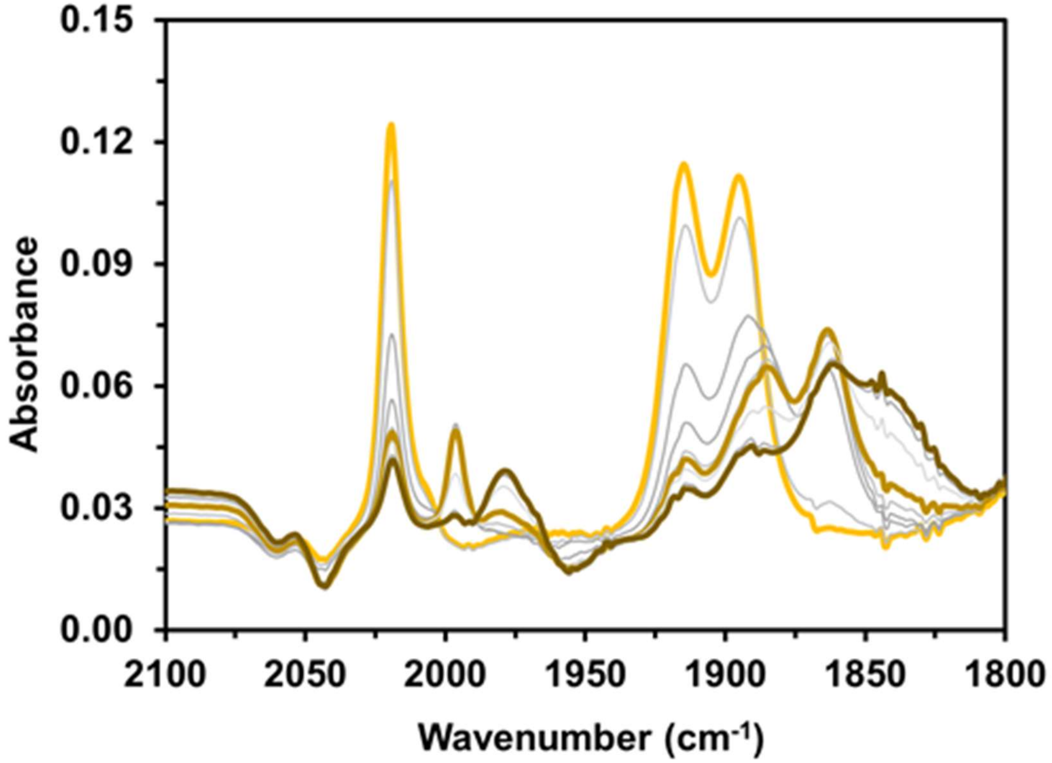

Figure S58. FTIR SEC absorbance spectra of Re(bpy-C2-NPDI-CN) obtained at an applied potential of R3 = -1.8 V vs. $Fc^{+/0}$. All experiments were performed in DMF with 0.1 M $TBAPF_6$ supporting electrolyte (WE = Pt-mesh, CE = Pt-wire, pseudo-RE = Ag-wire). Re^I(bpy-C2-NPDI-CN) (ν_{co} = 1894, 1915, 2019 cm^{-1}) undergoes a 3-electron reduction to give Re^I($bpy^{\bullet-}$-C2-$NPDI^{2-}$-CN) (ν_{co} = 1863, 1884, 1996 cm^{-1}). Over time, a chloro-dissociation equilibration process affords a 5-coordinate Re^0(bpy-C2-$NPDI^{2-}$-CN) species (ν_{co} = 1843, 1861, 1979 cm^{-1}).

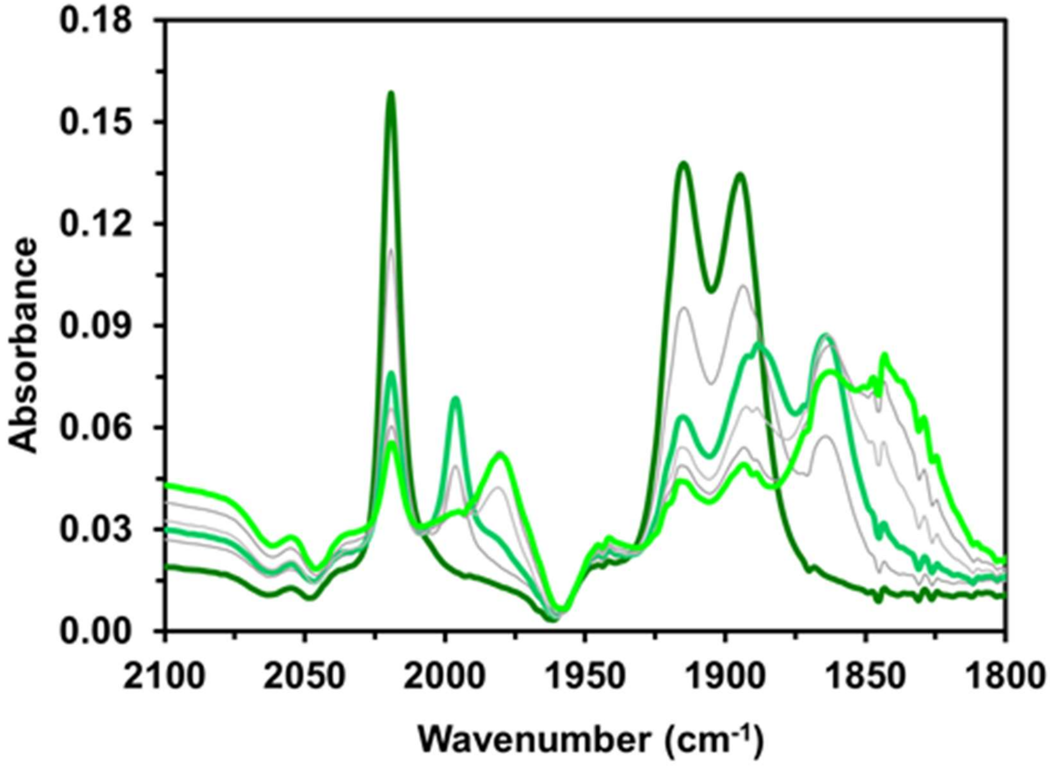

Figure S59. FTIR SEC absorbance spectra of Re(bpy-C2-NPDI-NO_2) obtained at an applied potential of R3 = -1.8 V vs. $Fc^{+/0}$. All experiments were performed in DMF with 0.1 M $TBAPF_6$ supporting electrolyte (WE = Pt-mesh, CE = Pt-wire, pseudo-RE = Ag-wire). Re^{I}(bpy-C2-NPDI-NO_2) (ν_{co} = 1894, 1915, 2019 cm^{-1}) undergoes a 3-electron reduction to give Re^{I}($bpy^{\bullet-}$-C2-$NPDI^{2-}$-NO_2) (ν_{co} = 1864, 1888, 1996 cm^{-1}). Over time, a chloro-dissociation equilibration process affords a 5-coordinate Re^{0}(bpy-C2-$NPDI^{2-}$-NO_2) species (ν_{co} = 1843, 1863, 1979 cm^{-1}).

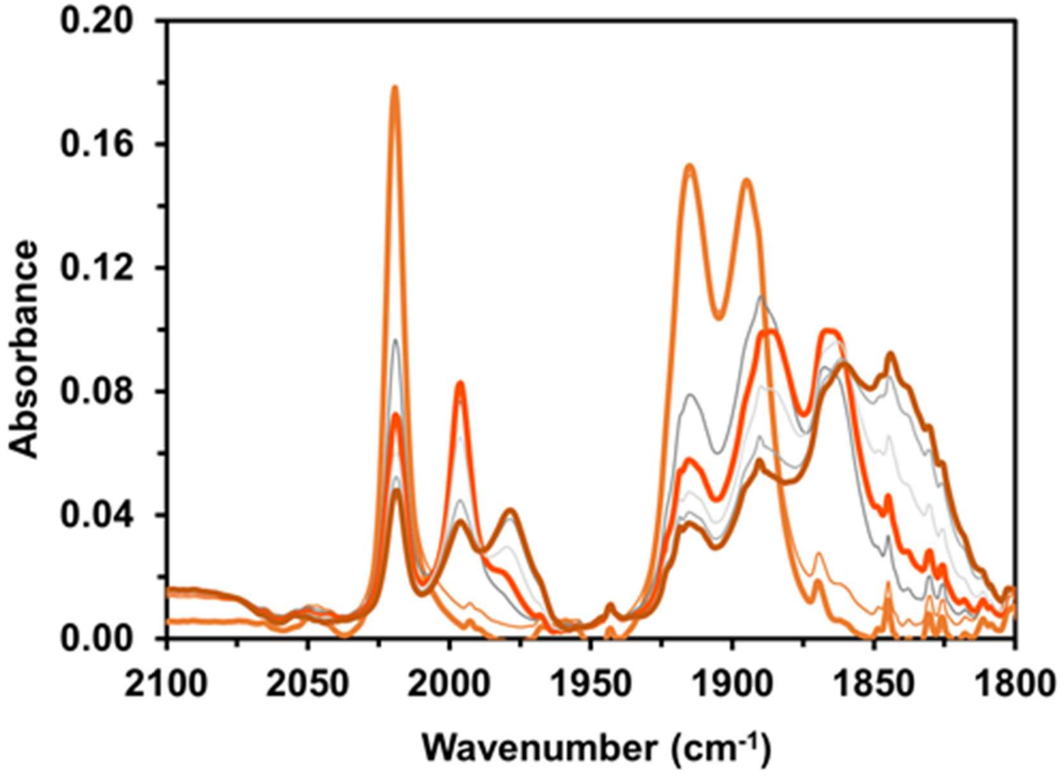

Figure S60. FTIR SEC absorbance spectra of Re(bpy-C2-NPDI-Br) obtained at an applied potential of R3 = -1.8 V vs. $Fc^{+/0}$. All experiments were performed in DMF with 0.1 M $TBAPF_6$ supporting electrolyte (WE = Pt-mesh, CE = Pt-wire, pseudo-RE = Ag-wire). Re^{I}(bpy-C2-NPDI-Br) (ν_{co} = 1894, 1915, 2019 cm^{-1}) undergoes a 3-electron reduction to give Re^{I}(bpy$^{\bullet-}$-C2-NPDI^{2-}-Br) (ν_{co} = 1865, 1886, 1996 cm^{-1}). Over time, a chloro-dissociation equilibration process affords a 5-coordinate Re^{0}(bpy-C2-NPDI^{2-}-Br) species (ν_{co} = 1843, 1860, 1977 cm^{-1}).

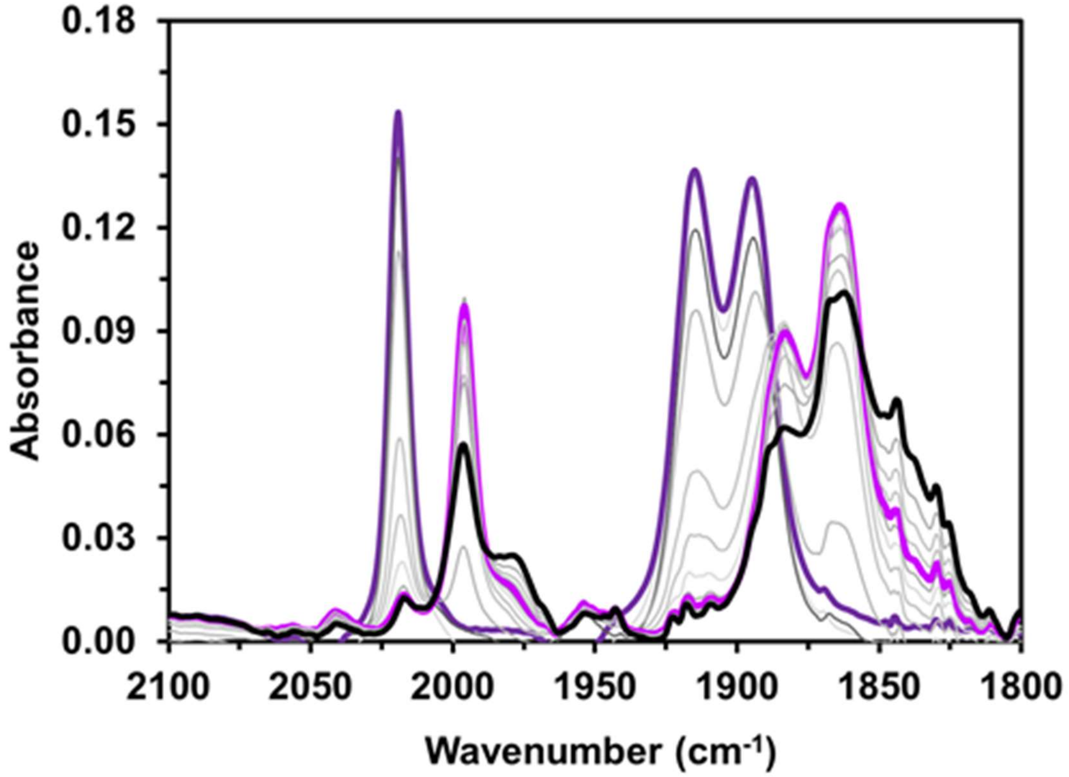

Figure S61. FTIR SEC absorbance spectra of Re(bpy-C2-NPDI-NR_2) obtained at an applied potential of R3 = -1.8 V vs. $Fc^{+/0}$. All experiments were performed in DMF with 0.1 M $TBAPF_6$ supporting electrolyte (WE = Pt-mesh, CE = Pt-wire, pseudo-RE = Ag-wire). Re^I(bpy-C2-NPDI-NR_2) (ν_{co} = 1894, 1915, 2019 cm^{-1}) undergoes a 3-electron reduction to give Re^I($bpy^{\bullet-}$-C2-$NPDI^{2-}$-NR_2) (ν_{co} = 1864, 1883, 1996 cm^{-1}). Only a small degree of chloro-dissociation to give the 5-coordinate Re^0(bpy-C2-$NPDI^{2-}$-NR_2) species (ν_{co} = 1843, 1861, 1977 cm^{-1}) was observed.

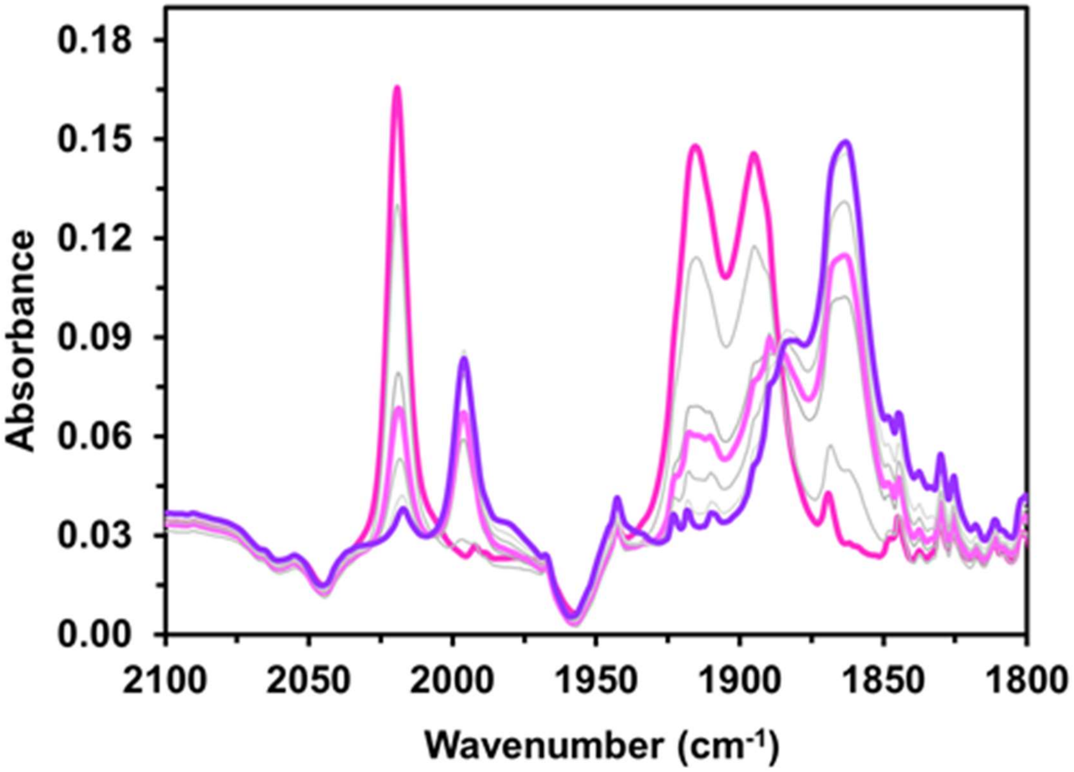

Figure S62. FTIR SEC absorbance spectra of Re(bpy-C2-NPDI-OPh) obtained at an applied potential of R3 = -1.8 V vs. $Fc^{+/0}$. All experiments were performed in DMF with 0.1 M $TBAPF_6$ supporting electrolyte (WE = Pt-mesh, CE = Pt-wire, pseudo-RE = Ag-wire). Re^{I}(bpy-C2-NPDI-OPh) (ν_{co} = 1894, 1914, 2019 cm^{-1}) undergoes a 3-electron reduction to give Re^{I}($bpy^{\bullet-}$-C2-$NPDI^{2-}$-OPh) (ν_{co} = 1862, 1885, 1997 cm^{-1}). Only a small degree of chloro-dissociation to give the 5-coordinate Re^{0}(bpy-C2-$NPDI^{2-}$-OPh) species (ν_{co} = 1844, 1862, 1977 cm^{-1}) was observed.

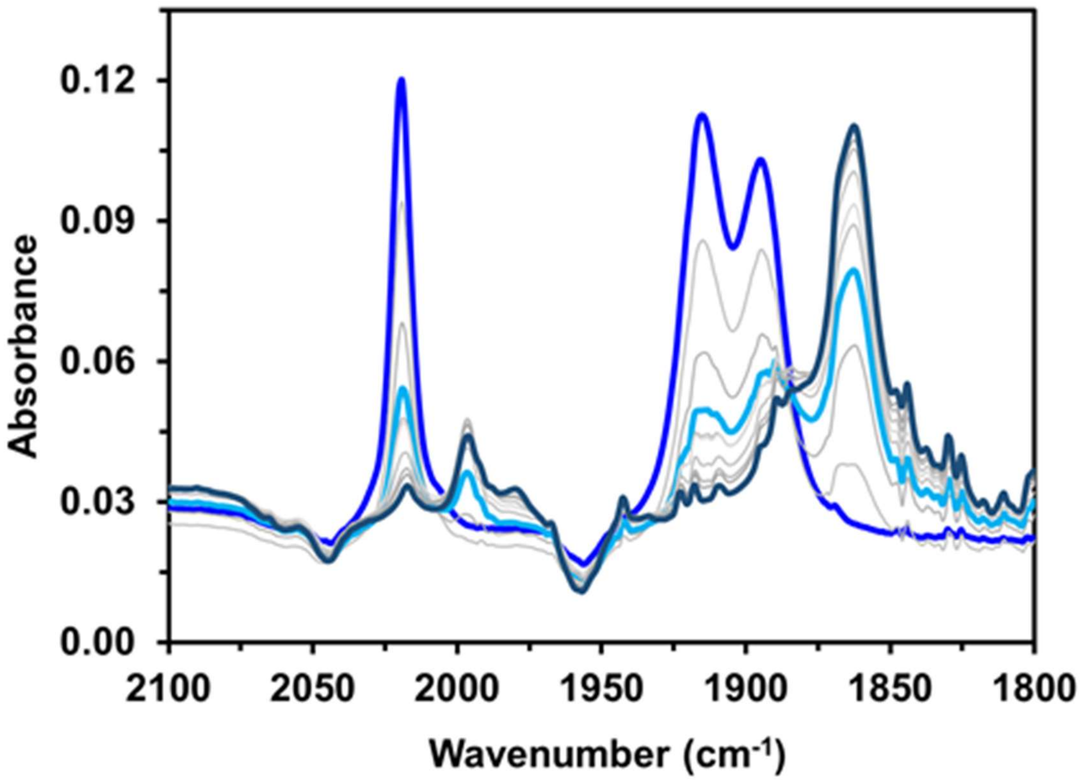

Figure S63. FTIR SEC absorbance spectra of Re(bpy-C2-NPDI-NH_2) obtained at an applied potential of R3 = -1.8 V vs. $Fc^{+/0}$. All experiments were performed in DMF with 0.1 M $TBAPF_6$ supporting electrolyte (WE = Pt-mesh, CE = Pt-wire, pseudo-RE = Ag-wire). Re^{I}(bpy-C2-NPDI-NH_2) (ν_{co} = 1894, 1915, 2019 cm^{-1}) undergoes a 3-electron reduction to give Re^{I}($bpy^{\bullet-}$-C2-$NPDI^{2-}$-NH_2) (ν_{co} = 1863, 1888, 1996 cm^{-1}). Only a small degree of chloro-dissociation to give the 5-coordinate Re^{0}(bpy-C2-$NPDI^{2-}$-OPh) species (ν_{co} = 1844, 1862, 1978 cm^{-1}) was observed.

6. Cyclic Voltammetry

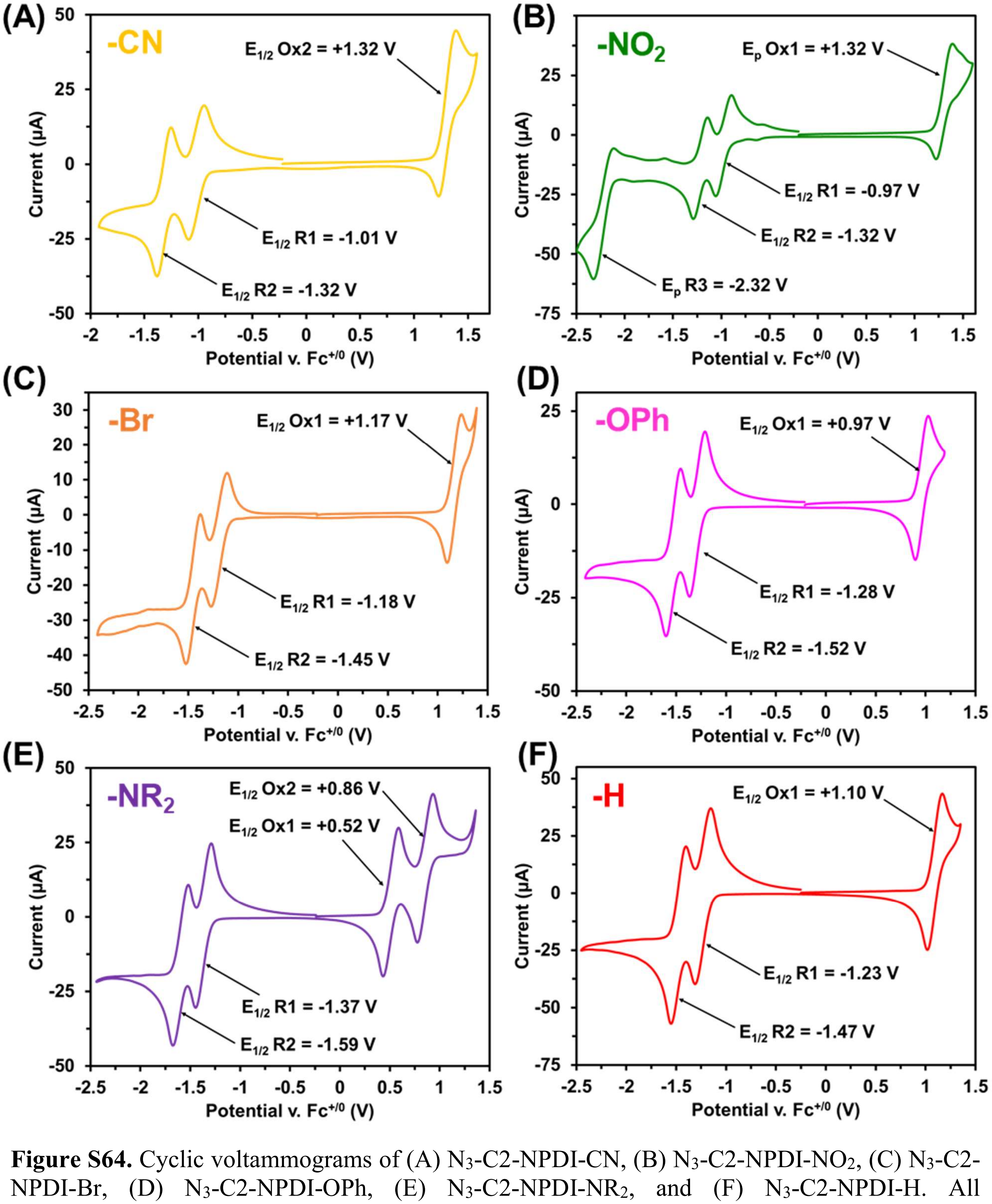

Figure S64. Cyclic voltammograms of (A) N_3-C2-NPDI-CN, (B) N_3-C2-NPDI-NO_2, (C) N_3-C2-NPDI-Br, (D) N_3-C2-NPDI-OPh, (E) N_3-C2-NPDI-NR_2, and (F) N_3-C2-NPDI-H. All measurements were recorded at 100 mV/s, under argon in CH_2Cl_2 with 0.1 M $TBAPF_6$ supporting electrolyte (WE = glassy carbon, CE = Pt-wire, RE = Ag/AgCl, and $Fc^{+/0}$ as internal reference standard)

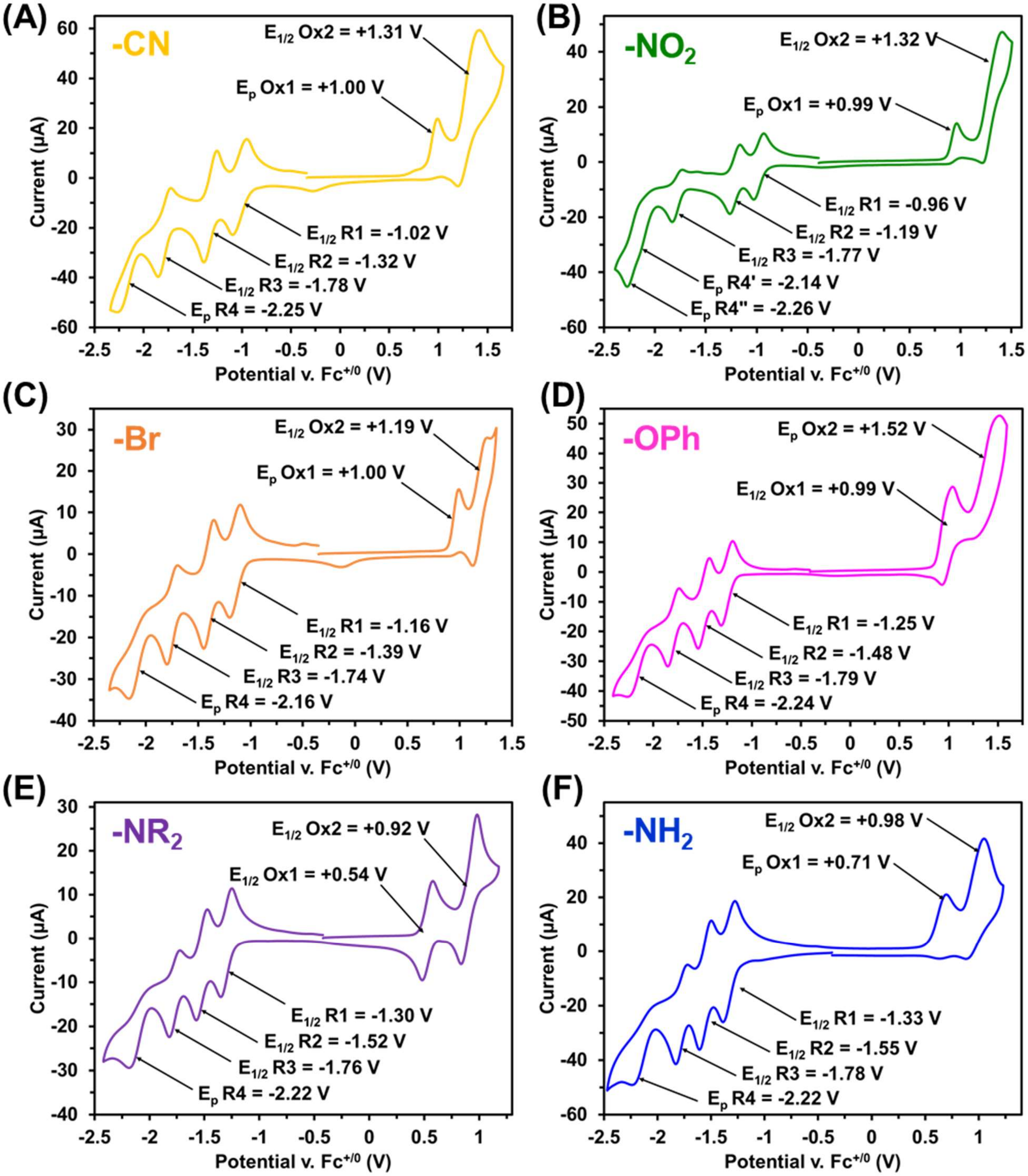

Figure S65. Cyclic voltammograms of (A) Re(bpy-C2-NPDI-CN), (B) Re(bpy-C2-NPDI-NO_2), (C) Re(bpy-C2-NPDI-Br), (D) Re(bpy-C2-NPDI-OPh), (E) Re(bpy-C2-NPDI-NR_2), and (F) Re(bpy-C2-NPDI-NH_2). All measurements were recorded at 100 mV/s, under argon in CH_2Cl_2 with 0.1 M $TBAPF_6$ supporting electrolyte (WE = glassy carbon, CE = Pt-wire, RE = Ag/AgCl, and $Fc^{+/0}$ as internal reference standard)

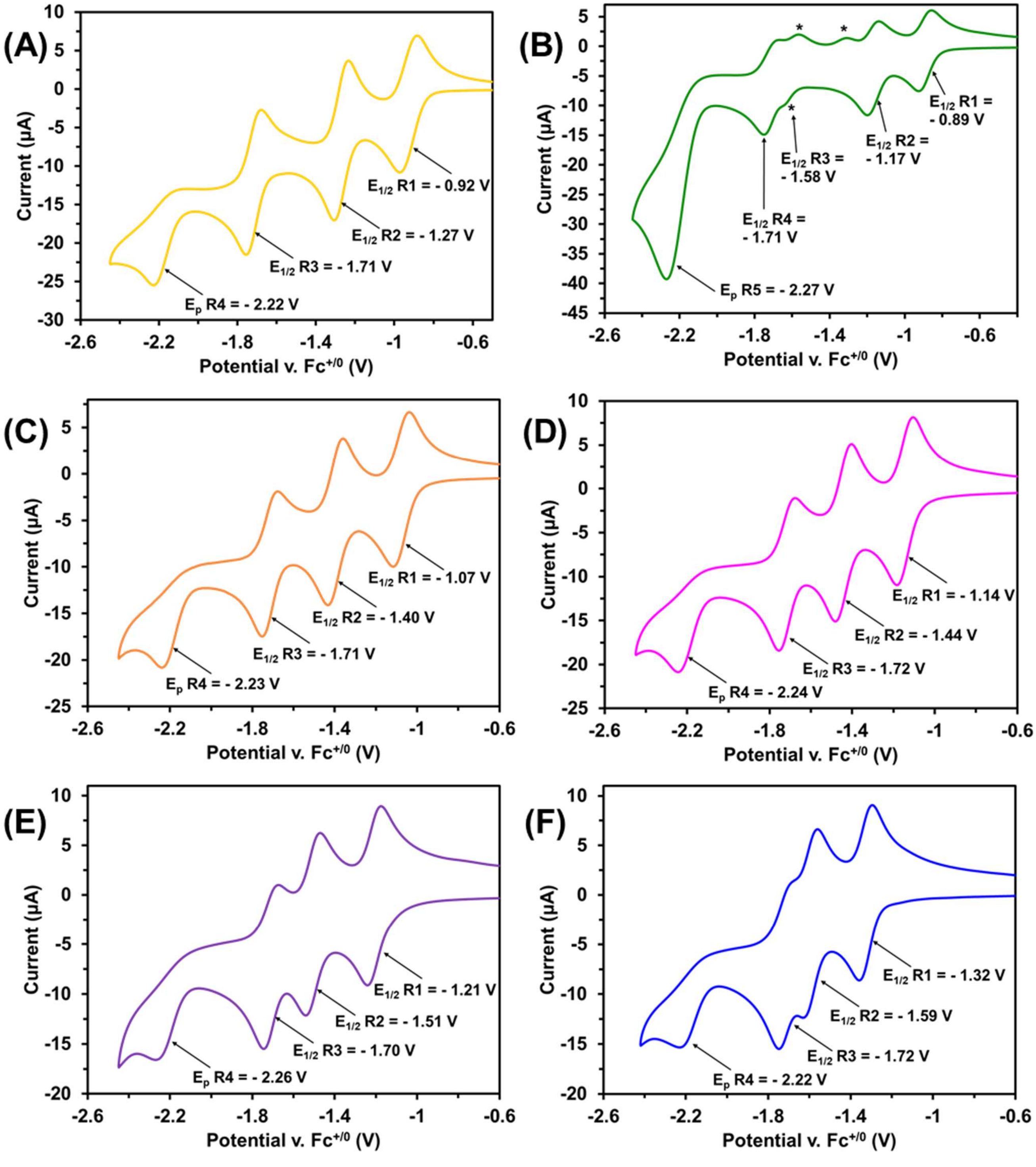

Figure S66. Cyclic voltammograms of reduction events in DMF for (A) Re(bpy-C2-NPDI-CN), (B) Re(bpy-C2-NPDI-NO_2), (C) Re(bpy-C2-NPDI-Br), (D) Re(bpy-C2-NPDI-OPh), (E) Re(bpy-C2-NPDI-NR_2), and (F) Re(bpy-C2-NPDI-NH_2). All measurements were recorded at 100 mV/s with 0.1 M $TBAPF_6$ supporting electrolyte (WE = glassy carbon, CE = Pt-wire, RE = Ag/AgCl, and $Fc^{+/0}$ as internal reference standard). *Note = (*) denote oxidation events which result from the in-situ conversion of Re(bpy-C2-NPDI-NO_2) to Re(bpy-C2-NPDI-NH_2)*

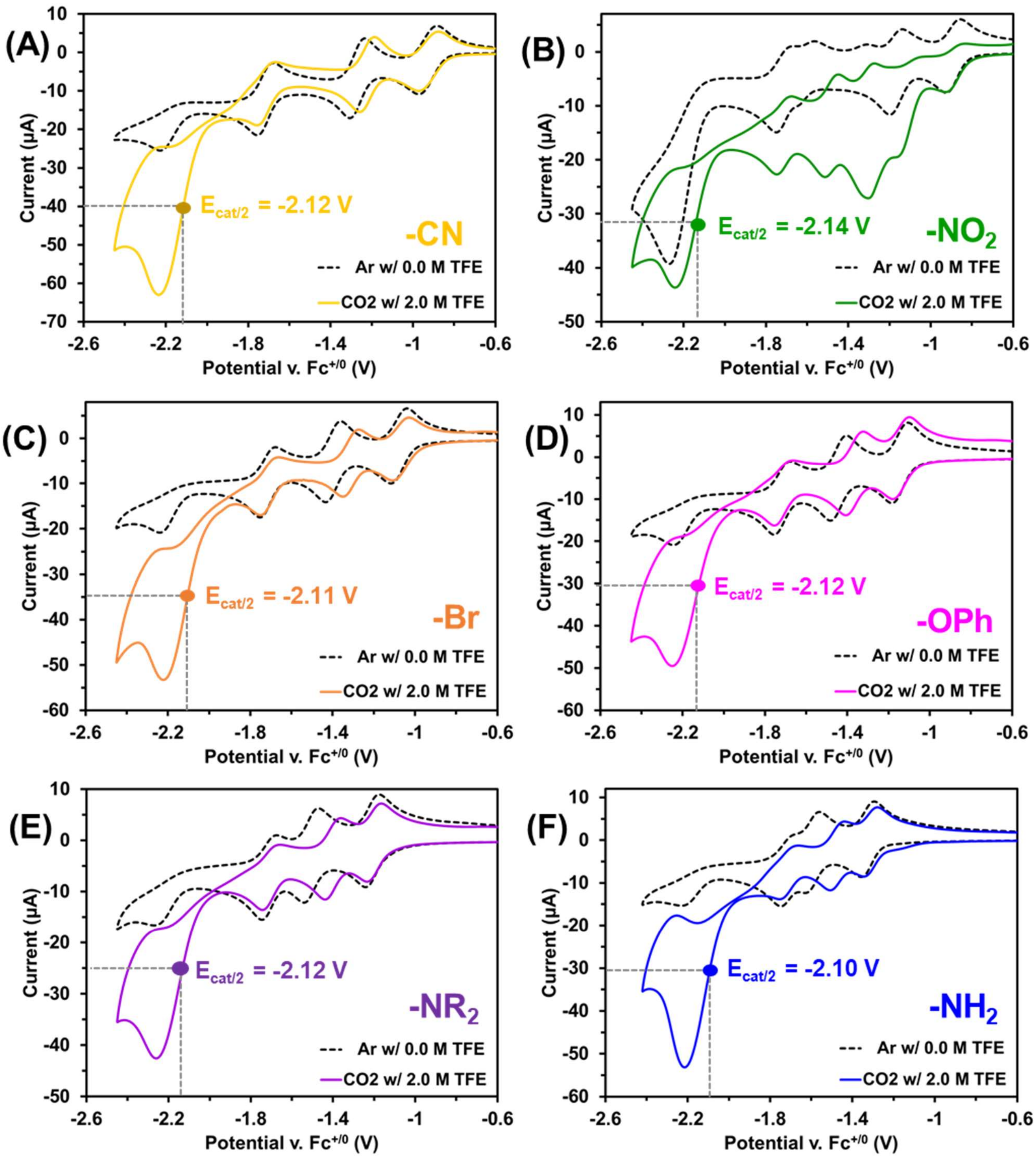

Figure S67. CV determined $E_{cat/2}$ values for (A) Re(bpy-C2-NPDI-CN), (B) Re(bpy-C2-NPDI-NO_2), (C) Re(bpy-C2-NPDI-Br), (D) Re(bpy-C2-NPDI-OPh), (E) Re(bpy-C2-NPDI-NR_2), and (F) Re(bpy-C2-NPDI-NH_2). All measurements were recorded at 100 mV/s, under argon in DMF with 0.1 M $TBAPF_6$ supporting electrolyte (WE = glassy carbon, CE = Pt-wire, RE = Ag/AgCl, and $Fc^{+/0}$ as internal reference standard)

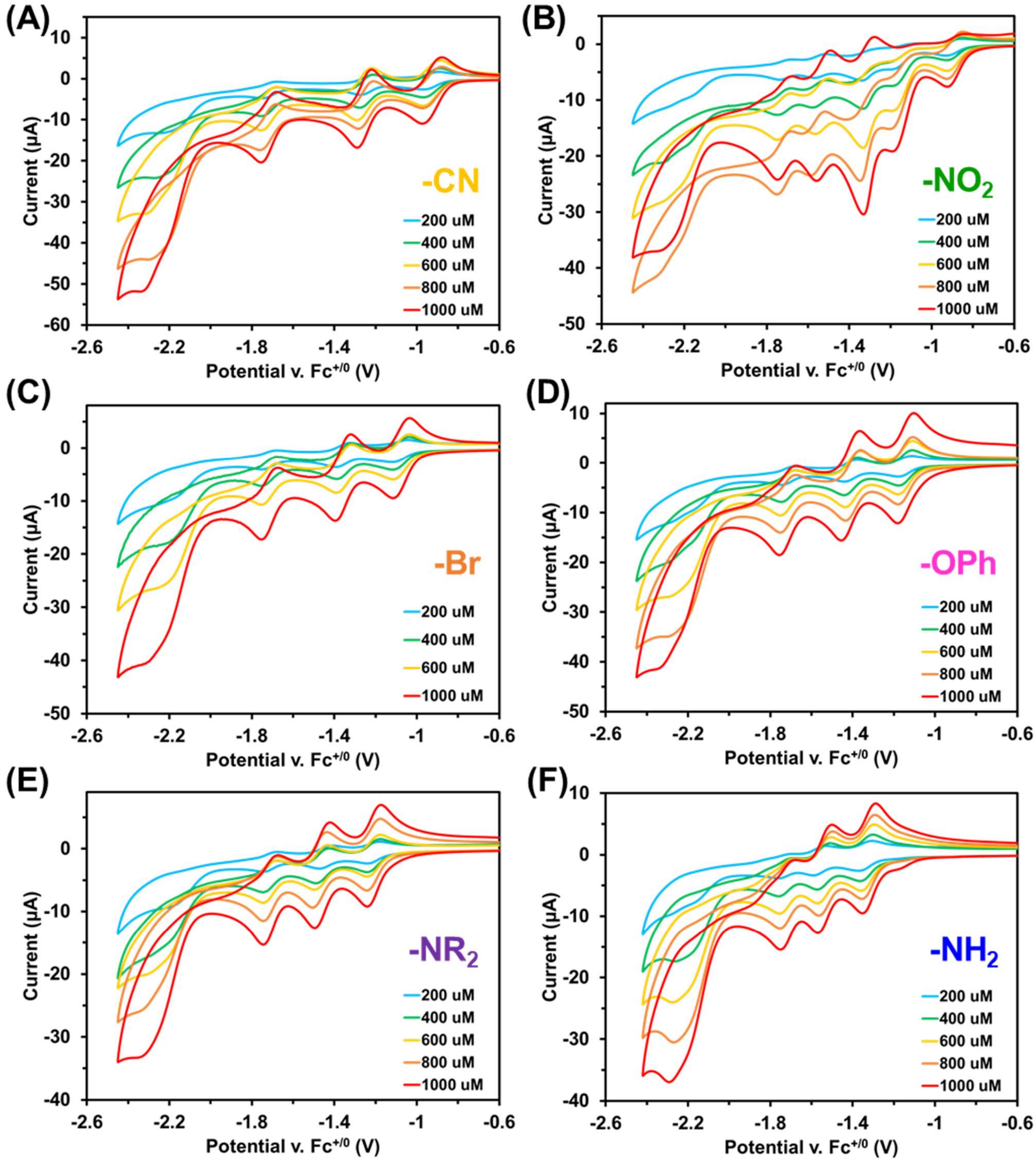

Figure S68. CV current enhancement effects as a function of (A) Re(bpy-C2-NPDI-CN), (B) Re(bpy-C2-NPDI-NO_2), (C) Re(bpy-C2-NPDI-Br), (D) Re(bpy-C2-NPDI-OPh), (E) Re(bpy-C2-NPDI-NR_2), and (F) Re(bpy-C2-NPDI-NH_2) concentration. Under an atmosphere of CO_2, CVs of each catalyst were measured at 0.2 mM (blue), 0.4 mM (green), 0.6 mM (yellow), 0.8 mM (orange), and 1.0 mM (red) were. All measurements were recorded at 100 mV/s, in DMF with 1 M TFE and 0.1 M $TBAPF_6$ supporting electrolyte (WE = glassy carbon, CE = Pt-wire, RE = Ag/AgCl, and $Fc^{+/0}$ as internal reference standard).

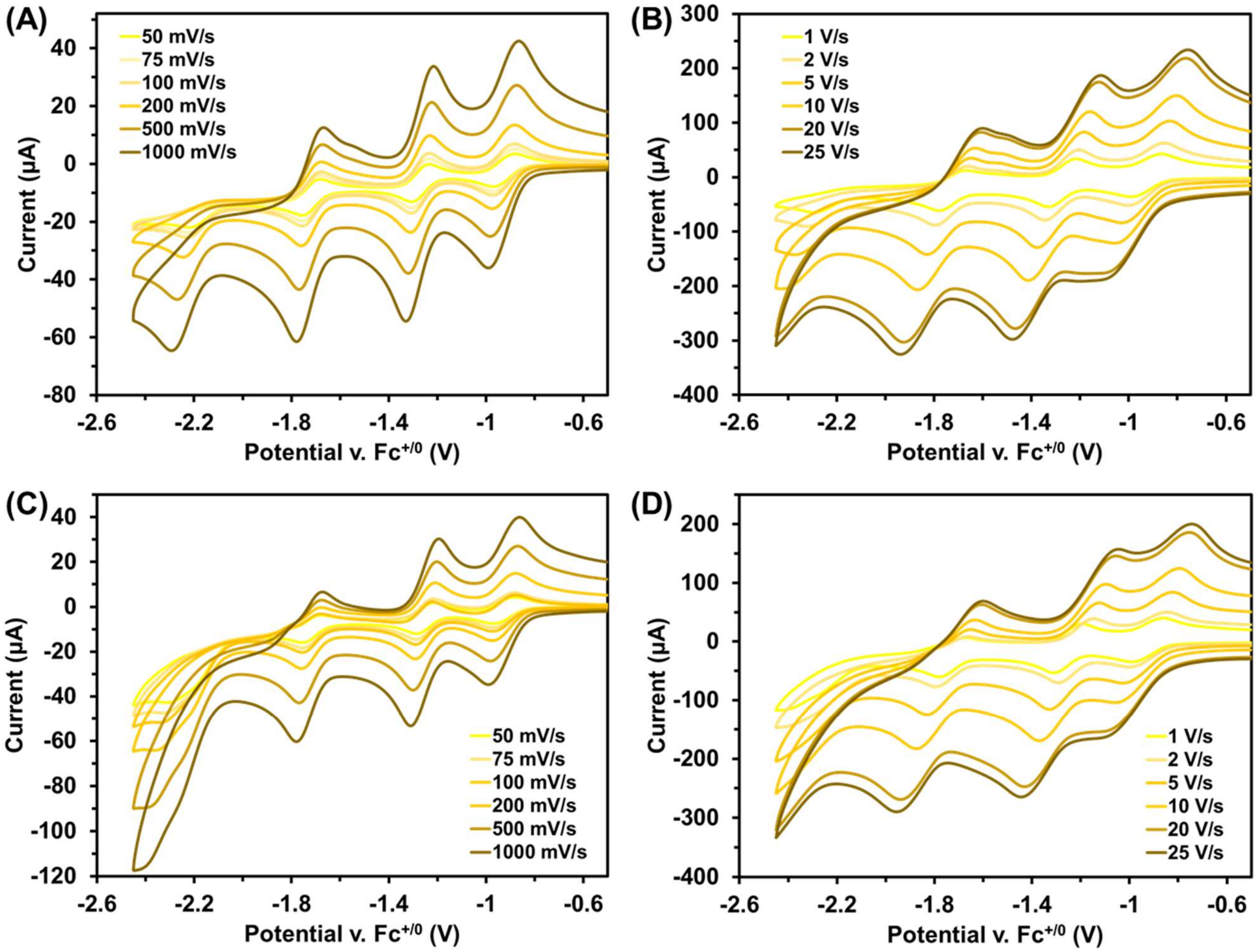

Figure S69. CVs of Re(bpy-C2-NPDI-CN) recorded at variable scan rate under argon (A and B) and under CO_2 (C and D) in DMF with 0.1 M $TBAPF_6$ supporting electrolyte (WE = glassy carbon, CE = Pt-wire, RE = Ag/AgCl, and $Fc^{+/0}$ as internal reference standard). Under argon, linear fitting of the scan rate to the Randles-Sevcik equation demonstrates that Re(bpy-C2-NPDI-CN) undergoes a diffusion-limited current response, with ***D = 3.4 x 10^{-6} cm^{2} s^{-1}.*** Using the i_{cat}/i_p method (in DMF with 2 M TFE at v = 25 V/s), the electrochemical CO_2 reduction rate constant was determined to be ***k_{obs} = 370 s^{-1}*** at $E_{cat/2}$ = -2.12 V (vs. $Fc^{+/0}$).

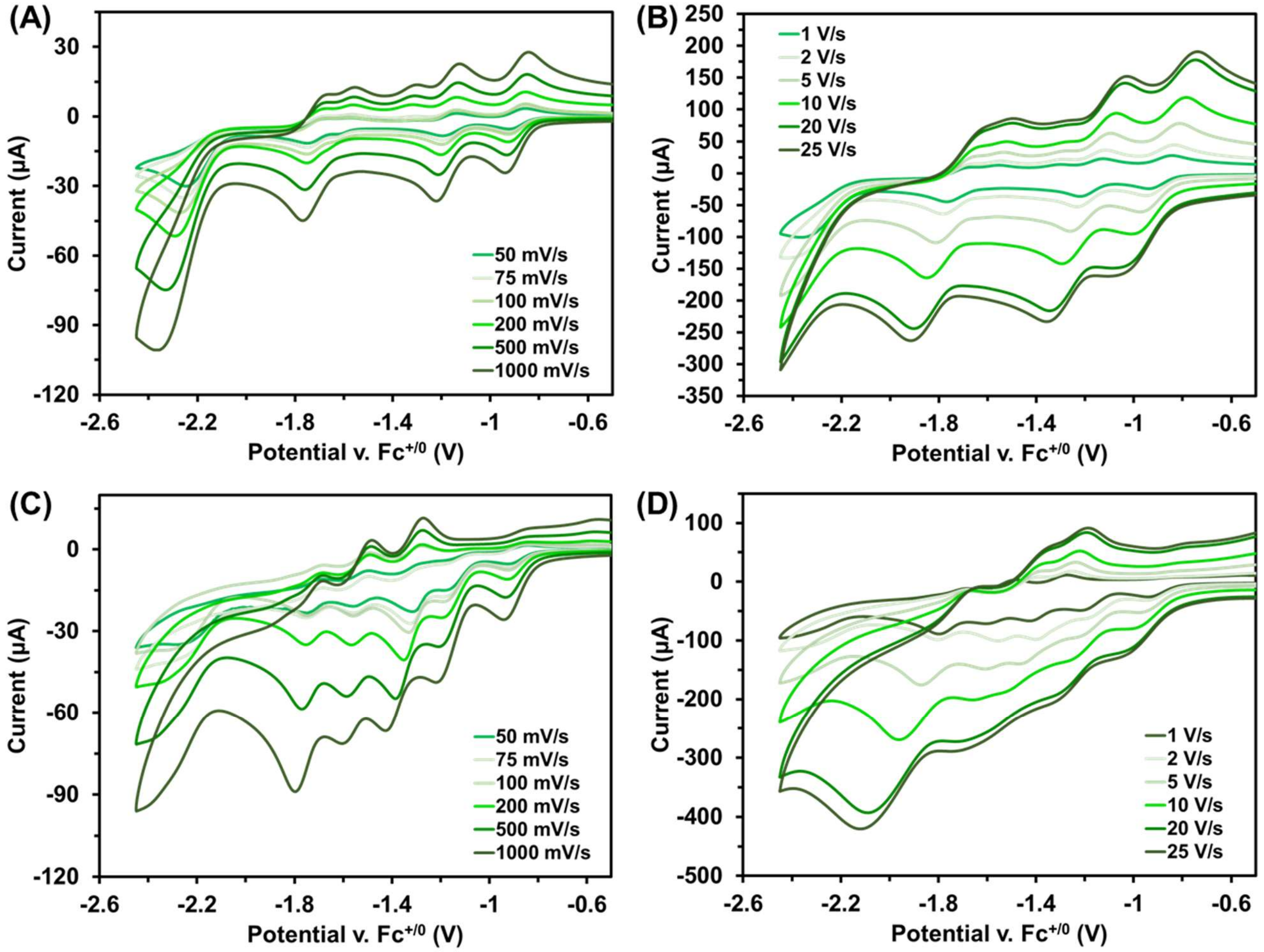

Figure S70. CVs of Re(bpy-C2-NPDI-NO_2) recorded at variable scan rate under argon (A and B) and under CO_2 (C and D) in DMF with 0.1 M $TBAPF_6$ supporting electrolyte (WE = glassy carbon, CE = Pt-wire, RE = Ag/AgCl, and $Fc^{+/0}$ as internal reference standard). Under argon, linear fitting of the scan rate to the Randles-Sevcik equation demonstrates that Re(bpy-C2-NPDI-NO_2) undergoes a diffusion-limited current response, with ***$D = 2.4 \times 10^{-6}$ cm² s⁻¹***. Using the i_{cat}/i_p method (in DMF with 2 M TFE at v = 25 V/s), the electrochemical CO_2 reduction rate constant was determined to be ***$k_{obs} = 523\ s^{-1}$*** at $E_{cat/2}$ = -2.14 V (vs. $Fc^{+/0}$).

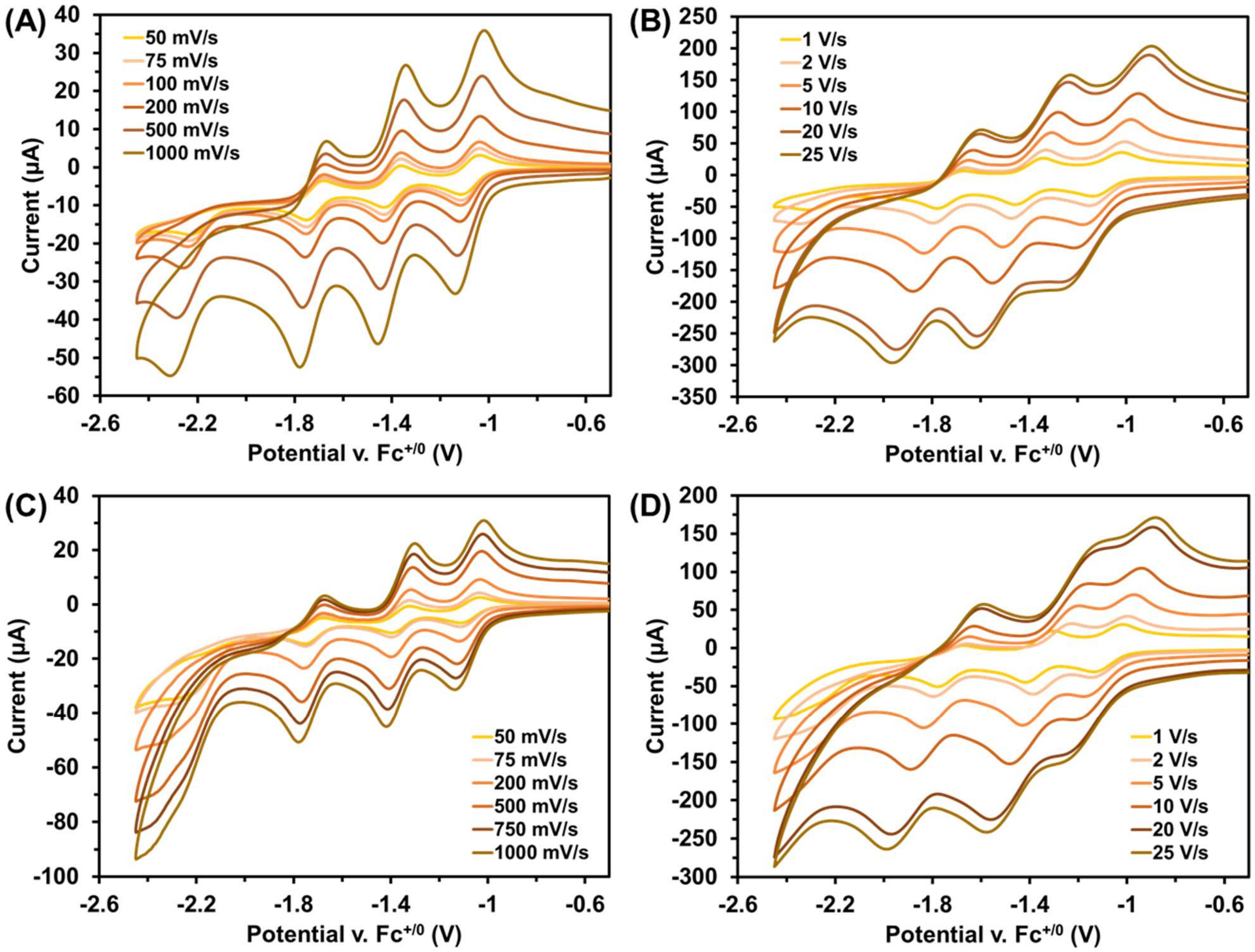

Figure S71. CVs of Re(bpy-C2-NPDI-Br) recorded at variable scan rate under argon (A and B) and under CO_2 (C and D) in DMF with 0.1 M $TBAPF_6$ supporting electrolyte (WE = glassy carbon, CE = Pt-wire, RE = Ag/AgCl, and $Fc^{+/0}$ as internal reference standard). Under argon, linear fitting of the scan rate to the Randles-Sevcik equation demonstrates that Re(bpy-C2-NPDI-Br) undergoes a diffusion-limited current response, with ***$D = 2.9 \times 10^{-6}$ cm^2 s^{-1}***. Using the i_{cat}/i_p method (in DMF with 2 M TFE at v = 25 V/s), the electrochemical CO_2 reduction rate constant was determined to be ***k_{obs} = 351 s^{-1}*** at $E_{cat/2}$ = -2.11 V (vs. $Fc^{+/0}$).

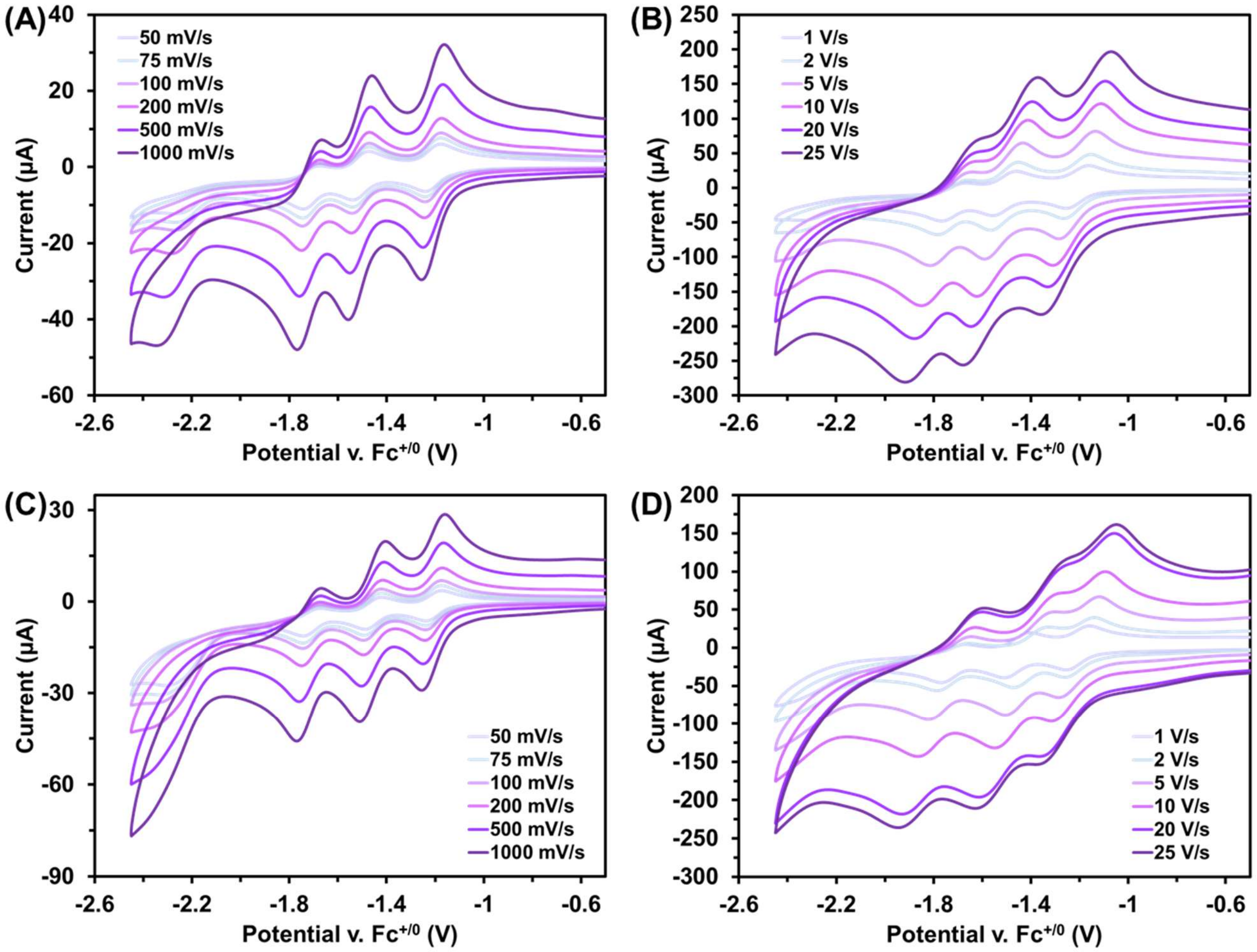

Figure S72. CVs of Re(bpy-C2-NPDI-NR$_2$) recorded at variable scan rate under argon (A and B) and under CO_2 (C and D) in DMF with 0.1 M TBAPF$_6$ supporting electrolyte (WE = glassy carbon, CE = Pt-wire, RE = Ag/AgCl, and Fc$^{+/0}$ as internal reference standard). Under argon, linear fitting of the scan rate to the Randles-Sevcik equation demonstrates that Re(bpy-C2-NPDI-NR$_2$) undergoes a diffusion-limited current response, with ***D = 4.4 x 10^{-6} cm^2 s^{-1}.*** Using the i_{cat}/i_p method (in DMF with 2 M TFE at v = 25 V/s), the electrochemical CO_2 reduction rate constant was determined to be ***k_{obs} = 332 s^{-1}*** at $E_{cat/2}$ = -2.12 V (vs. Fc$^{+/0}$).

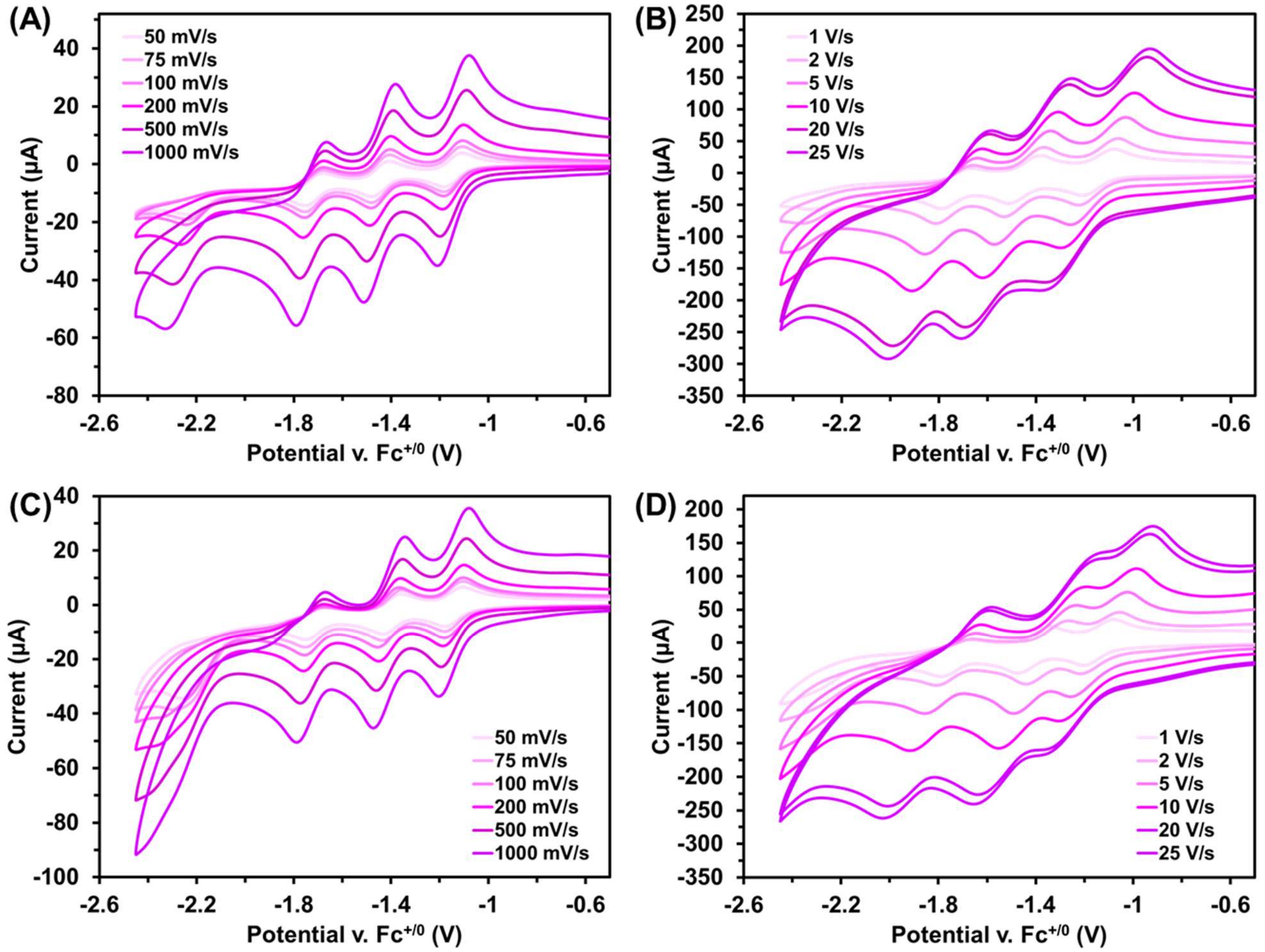

Figure S73. CVs of Re(bpy-C2-NPDI-OPh) recorded at variable scan rate under argon (A and B) and under CO_2 (C and D) in DMF with 0.1 M $TBAPF_6$ supporting electrolyte (WE = glassy carbon, CE = Pt-wire, RE = Ag/AgCl, and $Fc^{+/0}$ as internal reference standard). Under argon, linear fitting of the scan rate to the Randles-Sevcik equation demonstrates that Re(bpy-C2-NPDI-OPh) undergoes a diffusion-limited current response, with ***$D = 3.3 \times 10^{-6}$ cm^2 s^{-1}*.** Using the i_{cat}/i_p method (in DMF with 2 M TFE at v = 25 V/s), the electrochemical CO_2 reduction rate constant was determined to be ***$k_{obs} = 350$ s^{-1}*** at $E_{cat/2}$ = -2.12 V (vs. $Fc^{+/0}$).

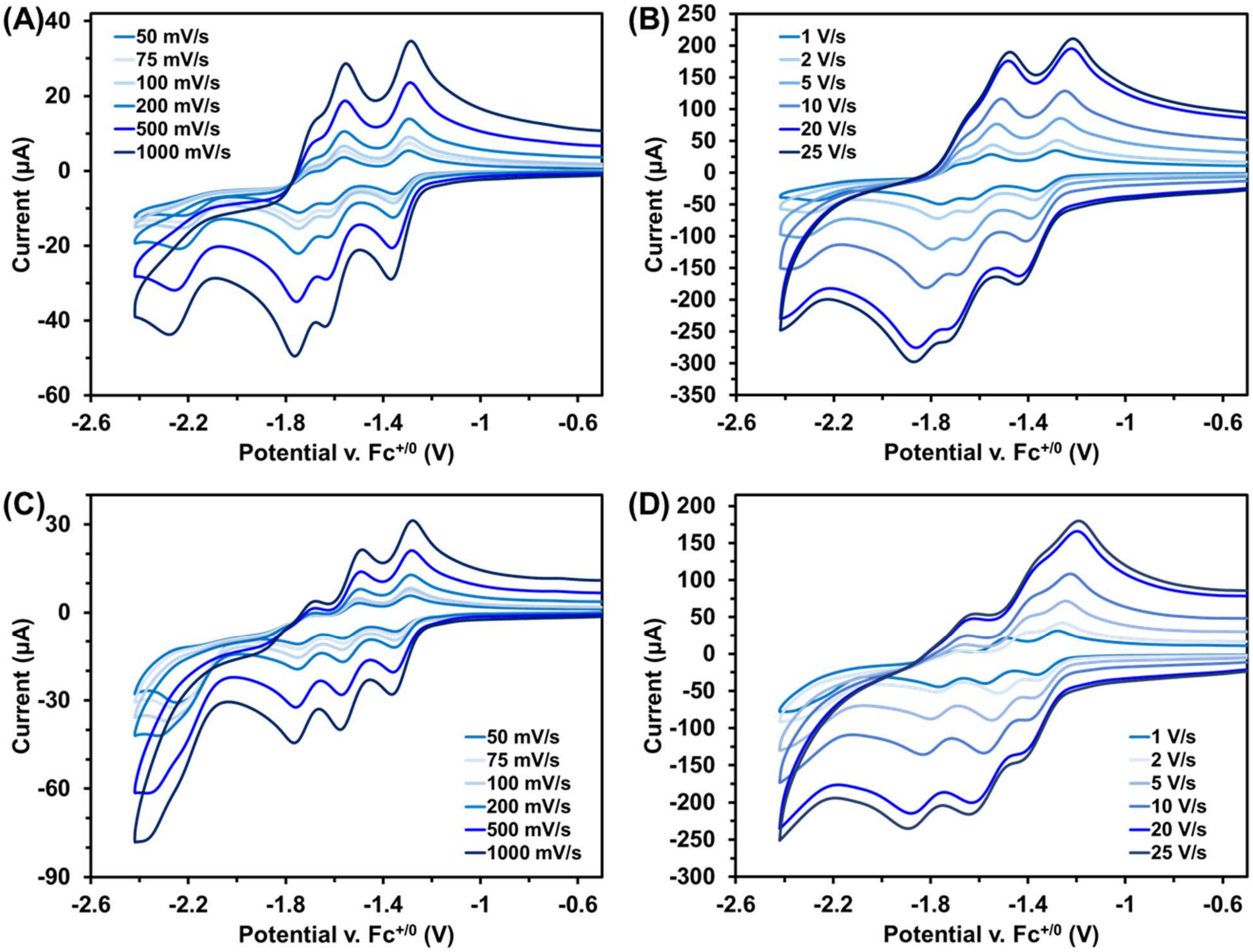

Figure S74. CVs of Re(bpy-C2-NPDI-NH_2) recorded at variable scan rate under argon (A and B) and under CO_2 (C and D) in DMF with 0.1 M $TBAPF_6$ supporting electrolyte (WE = glassy carbon, CE = Pt-wire, RE = Ag/AgCl, and $Fc^{+/0}$ as internal reference standard). Under argon, linear fitting of the scan rate to the Randles-Sevcik equation demonstrates that Re(bpy-C2-NPDI-NH_2) undergoes a diffusion-limited current response, with ***$D = 2.3 \times 10^{-6}$ cm^2 s^{-1}.*** Using the i_{cat}/i_p method (in DMF with 2 M TFE at v = 25 V/s), the electrochemical CO_2 reduction rate constant was determined to be ***$k_{obs} = 341$ s^{-1}*** at $E_{cat/2}$ = -2.10 V (vs. $Fc^{+/0}$).

7. Electro-/Photocatalytic CO_2 Reduction

Table S1. State-of-the-art Re(bpy)-chromophore dyads for photocatalytic CO_2 reduction

Entry	Catalyst	Testing Conditions	$TON_{CO/HCOOH}$	Ref.
A	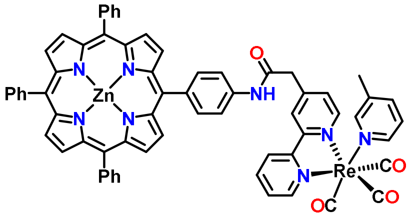	(5:1) DMF:TEOA<br>0.05 mM cat.<br>λ > 520 nm<br>7 hrs	CO = 332 ± 21	10
B	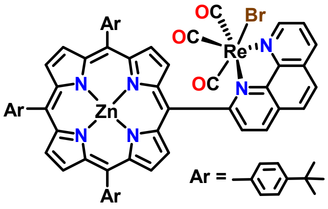	DMA w/<br>0.05 M BIH<br>0.1 M PhOH<br>0.05 mM cat.<br>λ = 420 nm<br>60 hrs	CO >1300	11
C	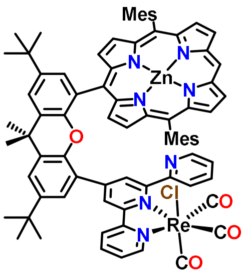	(4:1) DMF:TEOA<br>2000 equiv. BIH<br>0.05 mM cat.<br>λ > 450 nm<br>24 hrs	CO = 195	12
D	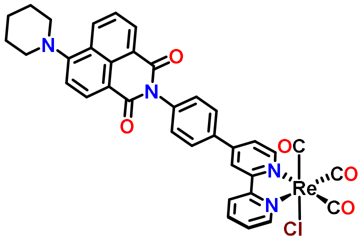	(5:1) DMF:TEOA<br>No BIH<br>2 mM cat.<br>λ = 450 ± 50 nm<br>~5 hrs	CO = 7.2	13
E	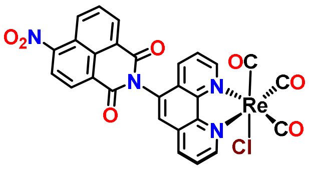	(1:9) CH_3OH:CH_2Cl_2<br>300 equiv. NEt_3<br>λ = 455 ± 5 nm<br>1.5 hrs	HCOOH = 50	14
F	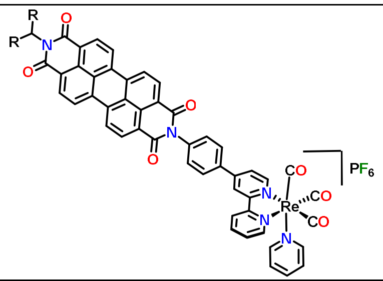	Not reported	Not reported	15

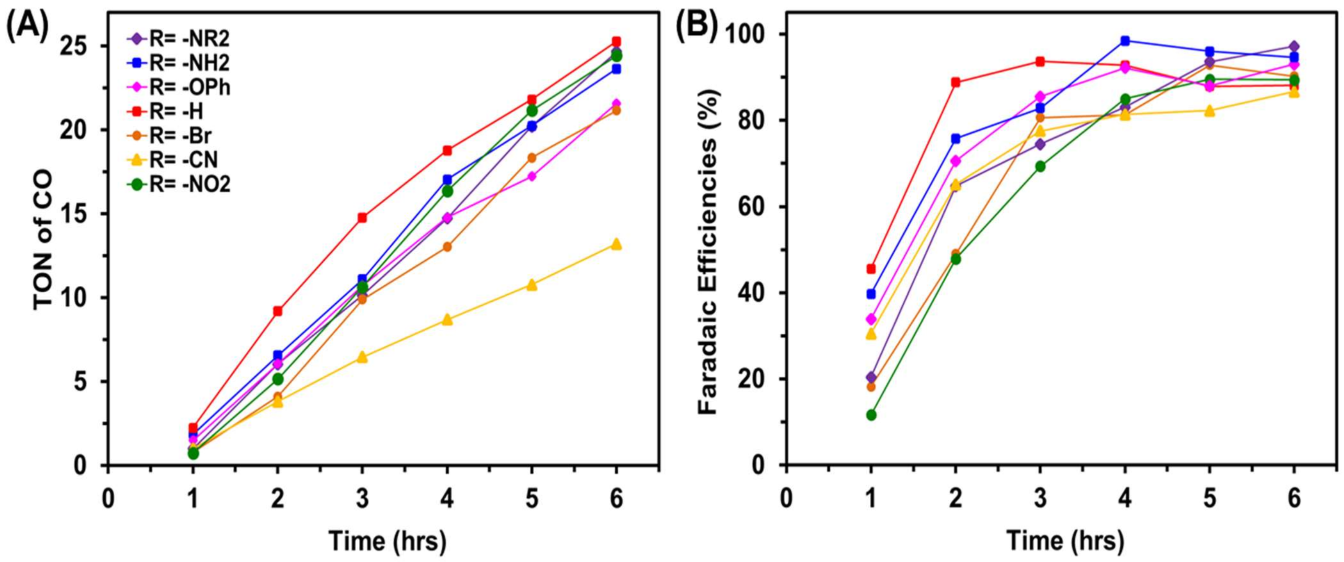

Figure S75. Comparing average TON_{co} (A) and faradaic efficiencies of CO formation (B) achieved by Re(bpy-C2-NPDI-NR_2) (purple), Re(bpy-C2-NPDI-NH_2) (blue), Re(bpy-C2-NPDI-OPh) (magenta), Re(bpy-C2-NPDI-H) (red), Re(bpy-C2-NPDI-Br) (orange), Re(bpy-C2-NPDI-CN) (yellow), and Re(bpy-C2-NPDI-NO_2) (green) during CPE experiments at E_{appl} = -1.8 vs. $Fc^{+/0}$.

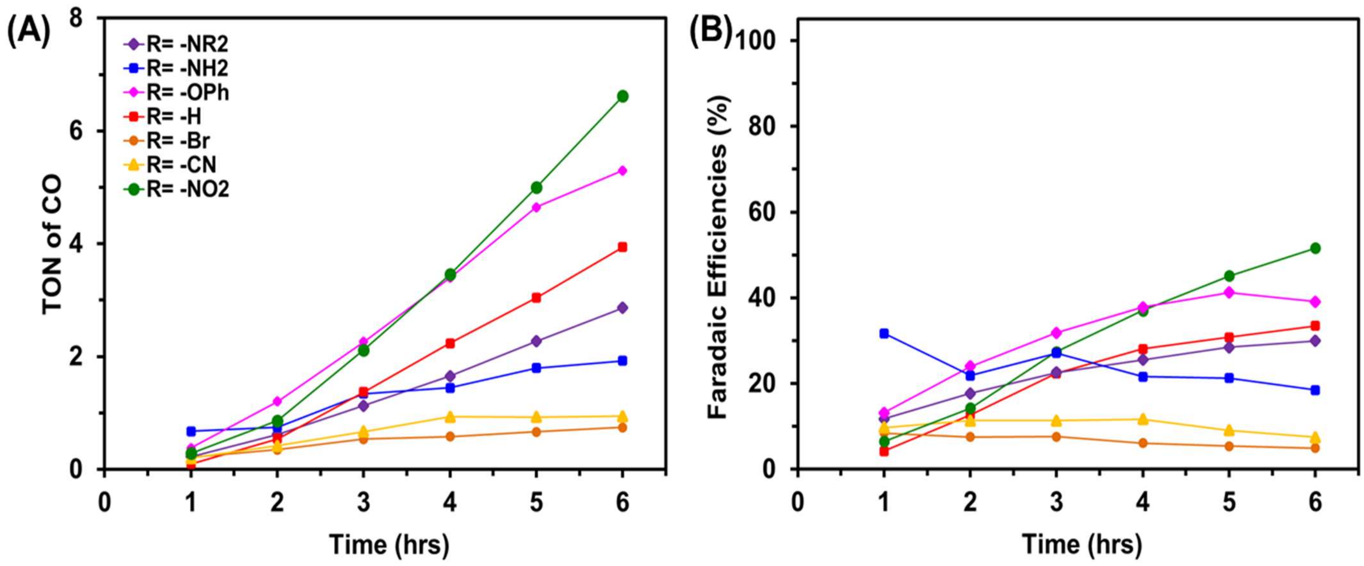

Figure S76. Comparing average TON_{co} (A) and faradaic efficiencies of CO formation (B) achieved by Re(bpy-C2-NPDI-NR_2) (purple), Re(bpy-C2-NPDI-NH_2) (blue), Re(bpy-C2-NPDI-OPh) (magenta), Re(bpy-C2-NPDI-H) (red), Re(bpy-C2-NPDI-Br) (orange), Re(bpy-C2-NPDI-CN) (yellow), and Re(bpy-C2-NPDI-NO_2) (green) during CPE experiments at E_{appl} = -1.7 vs. $Fc^{+/0}$.

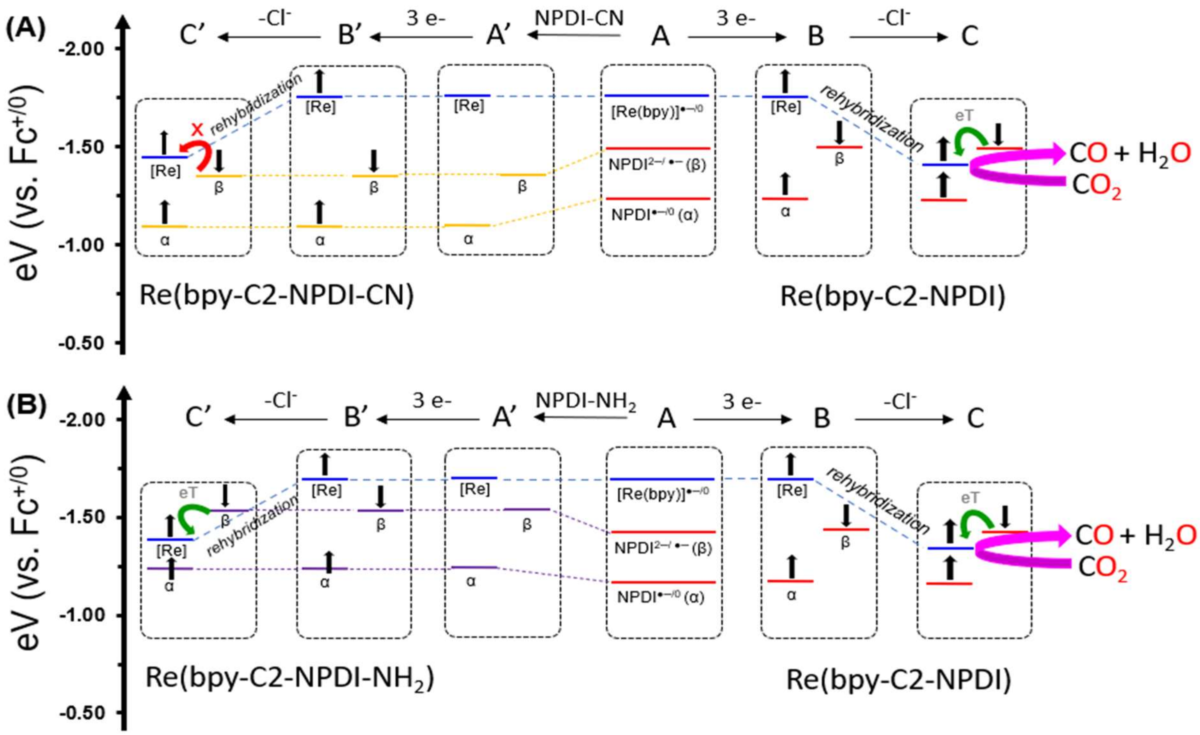

Figure S77. (A) MO description of Re(bpy-C2-NPDI-CN) (yellow) showing the effects of cyano-functionalization on shutting-down the electron-reservoir effect. (B) MO description of Re(bpy-C2-NPDI-NH_2) (purple) showing the effects of amino-functionalization on maintaining the electron-reservoir effect.

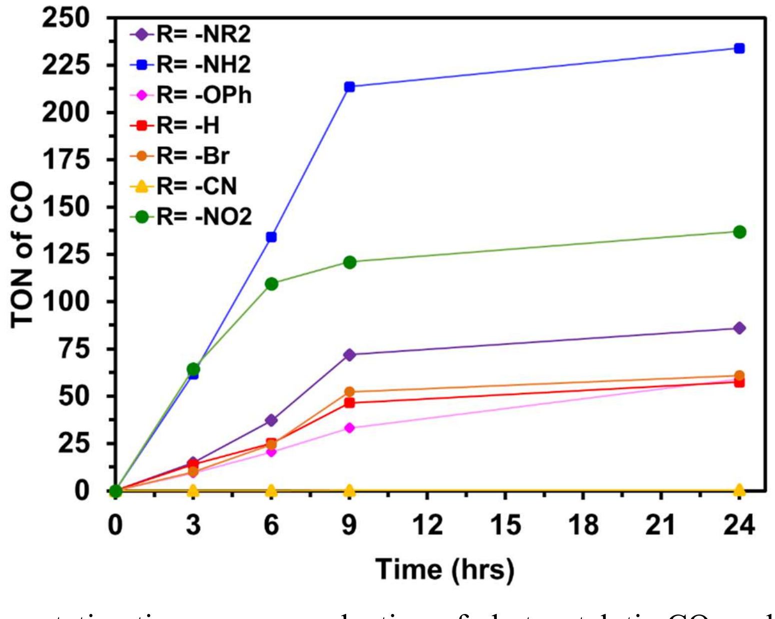

Figure S78. Representative time-course evaluation of photocatalytic CO_2 reduction for Re(bpy-C2-NPDI-NR_2) (purple), Re(bpy-C2-NPDI-NH_2) (blue), Re(bpy-C2-NPDI-OPh) (magenta), Re(bpy-C2-NPDI-H) (red), Re(bpy-C2-NPDI-Br) (orange), Re(bpy-C2-NPDI- CN) (yellow), and Re(bpy-C2-NPDI-NO_2) (green). Final results are summarized in Table 1 of the manuscript.

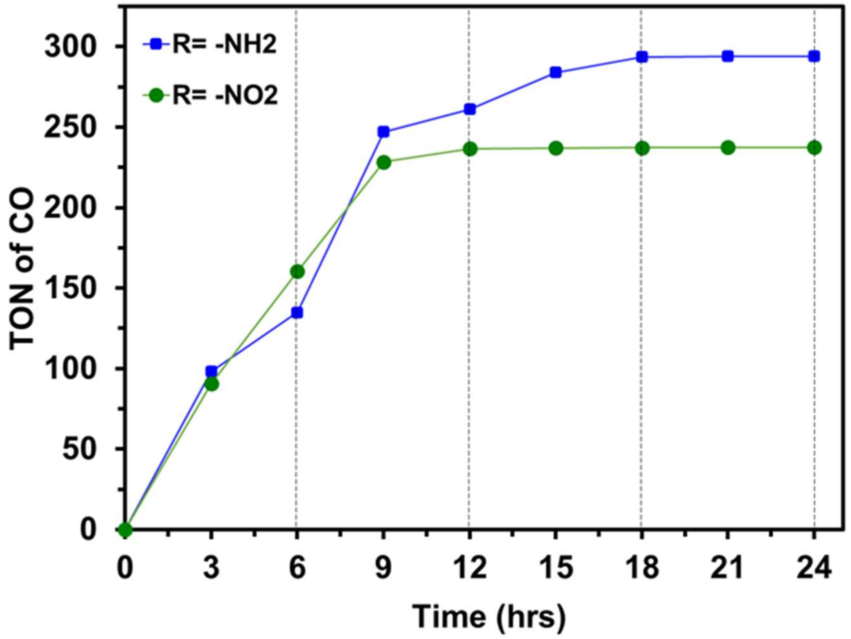

Figure S79. Time-interval experiment performed on Re(bpy-C2-NPDI-NH_2) (blue) and Re(bpy-C2-NPDI-NO_2) (green). After every 6 hours of photocatalysis, the samples were replenished with BIH and sparged with fresh CO_2 before restarting irradiation with blue light. Leveling-off of TON_{co} after ~9 hours suggests that substrate depletion does not contribute to catalyst degradation.

Table S2. Effect of sacrificial electron donor on photocatalytic CO_2 reduction of Re(bpy-C2-NPDI-R).

R =	TON_{CO}	TON_{H2}	CO:H_2
-H	*29*	*1.4*	*95:5*
-CN	*<1*	*<1*	---
-NO_2	*61*	*<1*	*>99:1*
-Br	*33*	*<1*	*>99:1*
-NR_2	*15*	*<1*	*>99:1*
-OPh	*22*	*<1*	*>99:1*
-NH_2	*65*	*1.8*	*97:3*

Table S3. Effect of solvent system on photocatalytic CO_2 reduction of Re(bpy-C2-NPDI-R).

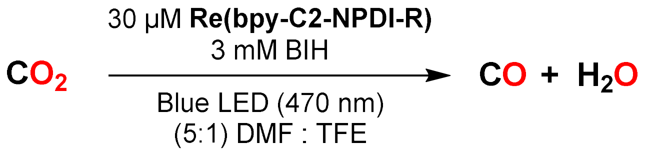

R =	TON_{CO}	TON_{H2}	$CO:H_2$
-H	*17*	*<1*	*>99:1*
-CN	*<1*	*<1*	---
$-NO_2$	*60*	*<1*	*>99:1*
-Br	*23*	*<1*	*>99:1*
$-NR_2$	*32*	*<1*	*>99:1*
-OPh	*34*	*<1*	*>99:1*
$-NH_2$	*143*	*<1*	*>99:1*

Table S4. Effect of light source on photocatalytic CO_2 reduction of Re(bpy-C2-NPDI-R).

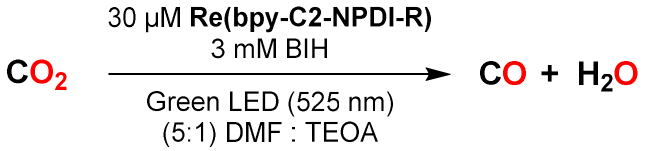

R =	TON_{CO}	TON_{H2}	$CO:H_2$
-H	*7.8*	*<1*	*99:1*
-CN	*<1*	*<1*	---
$-NO_2$	*22*	*<1*	*>99:1*
-Br	*8.2*	*<1*	*>99:1*
$-NR_2$	*6.9*	*<1*	*97:3*
-OPh	*11*	*<1*	*>99:1*
$-NH_2$	*27*	*<1*	*98:2*

Table S5. Photocatalytic CO_2 reduction of Re(bpy)(CO)$_3$Cl paired with N_3-C2-NPDI-R.

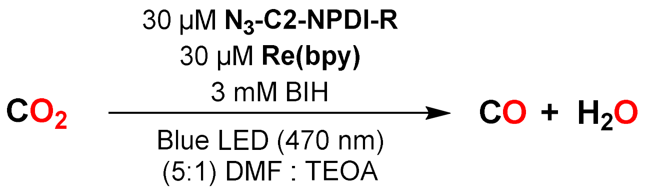

R =	TON_{CO}	TON_{H2}	$CO:H_2$
-H	*4.3*	*<1*	*>99:1*
-CN	*3.5*	*4.5*	*44:56*
$-NO_2$	*4.3*	*4.2*	*51:49*
-Br	*3.7*	*<1*	*>99:1*
$-NR_2$	*3.9*	*<1*	*>99:1*
-OPh	*3.4*	*<1*	*>99:1*
n/a*	*6.4*	*<1*	*>99:1*

* = experiment performed using 30 μM Re(bpy)(CO)$_3$Cl only

Table S6. Photocatalytic CO_2 reduction of N_3-C2-NPDI-R without Re(bpy).

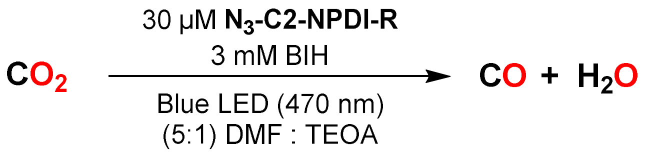

R =	TON_{CO}	TON_{H2}	$CO:H_2$
-H	*2.6*	*<1*	*>99:1*
-CN	*<1*	*<1*	---
$-NO_2$	*2.8*	*<1*	*>99:1*
-Br	*2.5*	*<1*	*>99:1*
$-NR_2$	*2.6*	*<1*	*>99:1*
-OPh	*<1*	*<1*	---

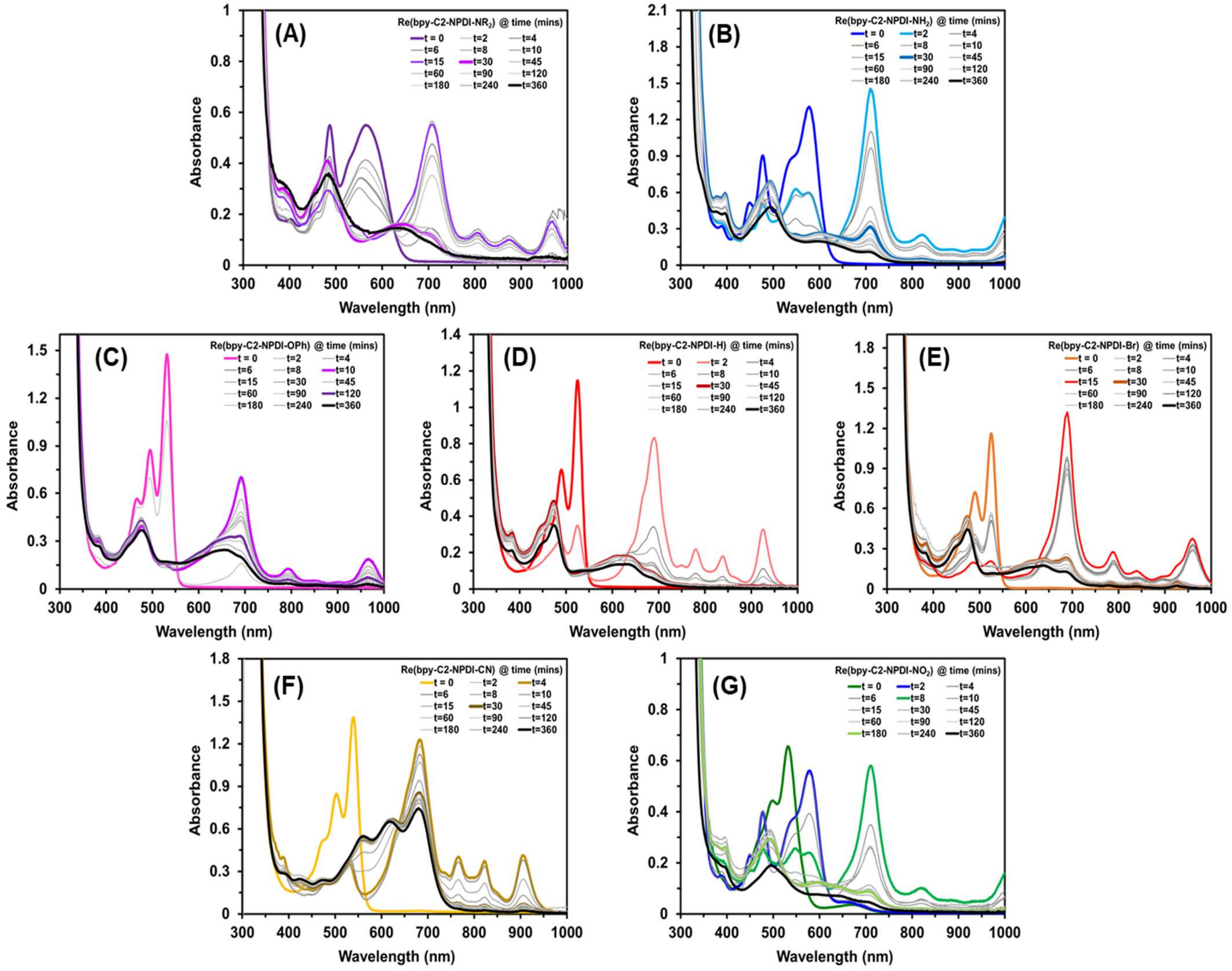

Figure S80. Monitoring of photocatalytic CO_2 intermediates by measuring periodic UV-vis-NIR absorption spectra of Re(bpy-C2-NPDI-R) derivatives, where R = $-NR_2$ (A), R = $-NH_2$ (B), R = -OPh (C), R = -H (D), R = -Br (E), R = -CN (F), and R = $-NO_2$ (G).

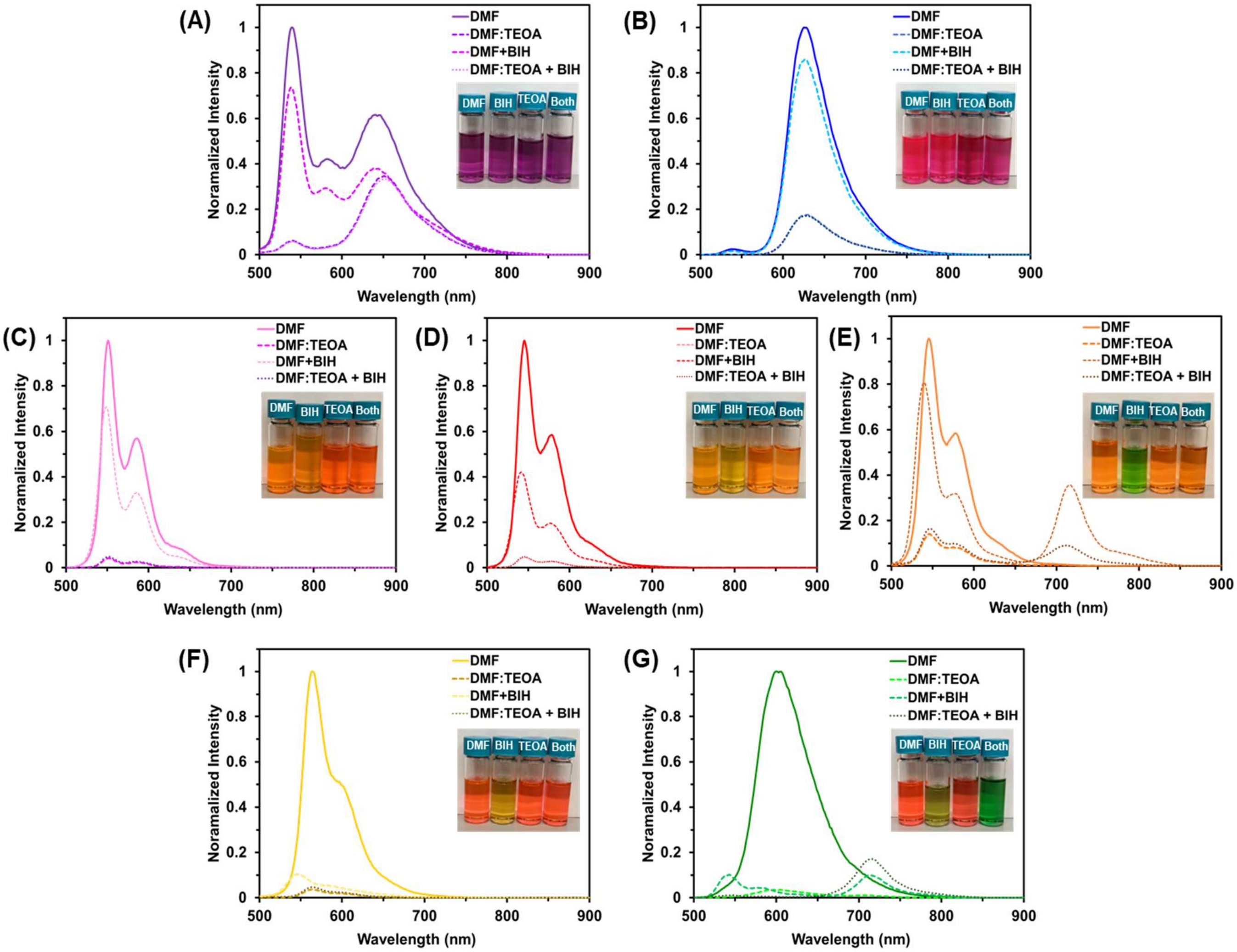

Figure S81. Monitoring the effects of BIH and TEOA on photoluminescence quenching of Re(bpy-C2-NPDI-R), where R = -NR_2 (A), R = -NH_2 (B), R = -OPh (C), R = -H (D), R = -Br (E), R = -CN (F), and R = -NO_2 (G). Samples were excited at λ_{ex} = 470 nm to match the photocatalytic CO_2 reduction conditions. All spectra for a particular Re(bpy-C2-NPDI-R) were normalized relative to the DMF only solution. In some cases (i.e., -Br and -NO_2), the added BIH was able to reduce the NPDI-moiety (after equilibrating for 4 hours), leading to the emergence of an emission feature at ~715 nm that correlates to the one-electron reduced species.

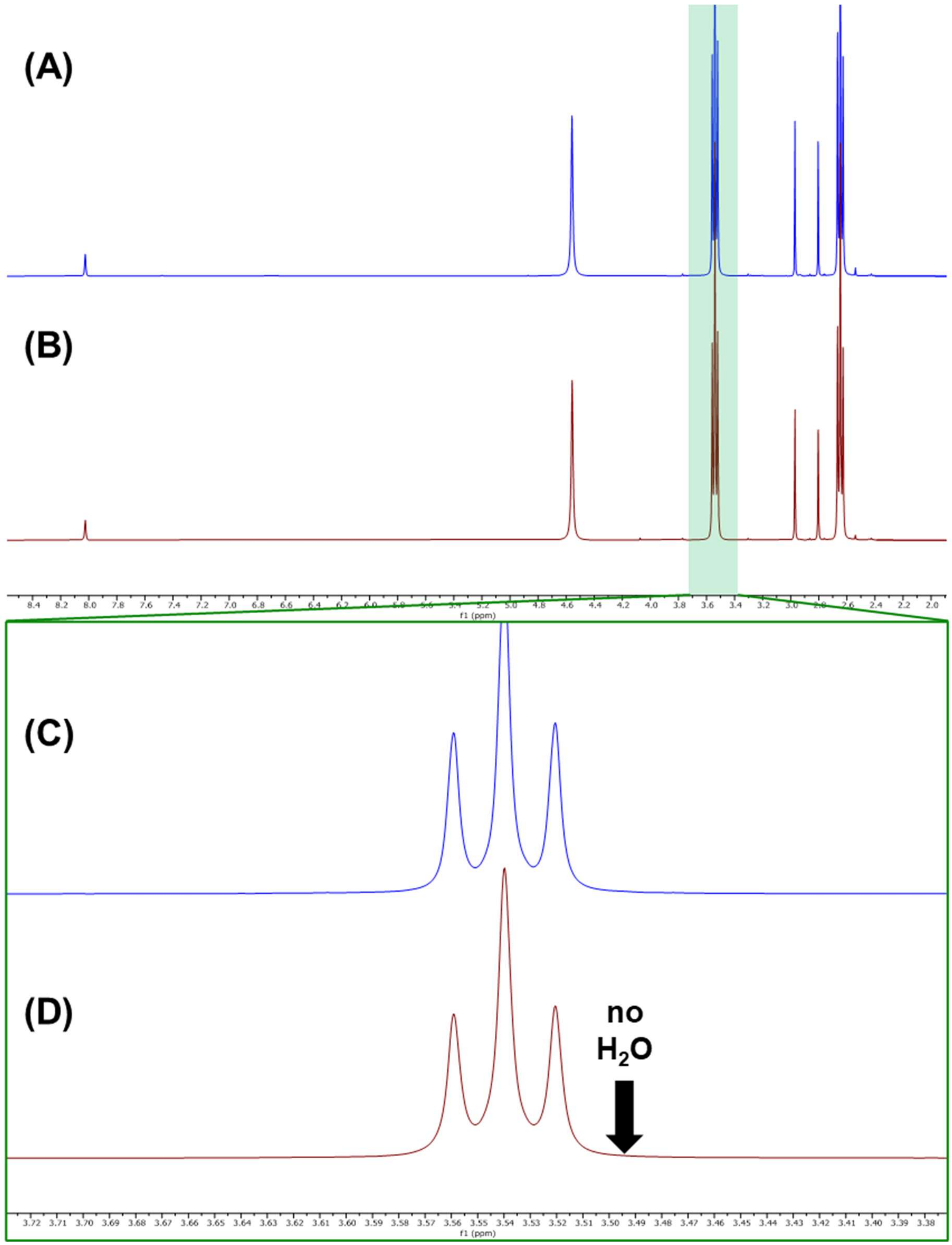

Figure S82. ^{1}H NMR spectra (300 MHz, DMF-d_7) of photocatalytic reaction mixture before (A & C) and after 24 hours of blue-light irradiation (B & D), where the reaction mixture was Re(bpy-C2-NPDI-NH_2) (30 μM), BIH (10 mM), and $^{13}CO_2$ (~0.9 atm) in a 5:1 DMF-d_7:TEOA mixture. There is no evidence of H_2O (δ 3.5 ppm) formation during photocatalysis.[16]

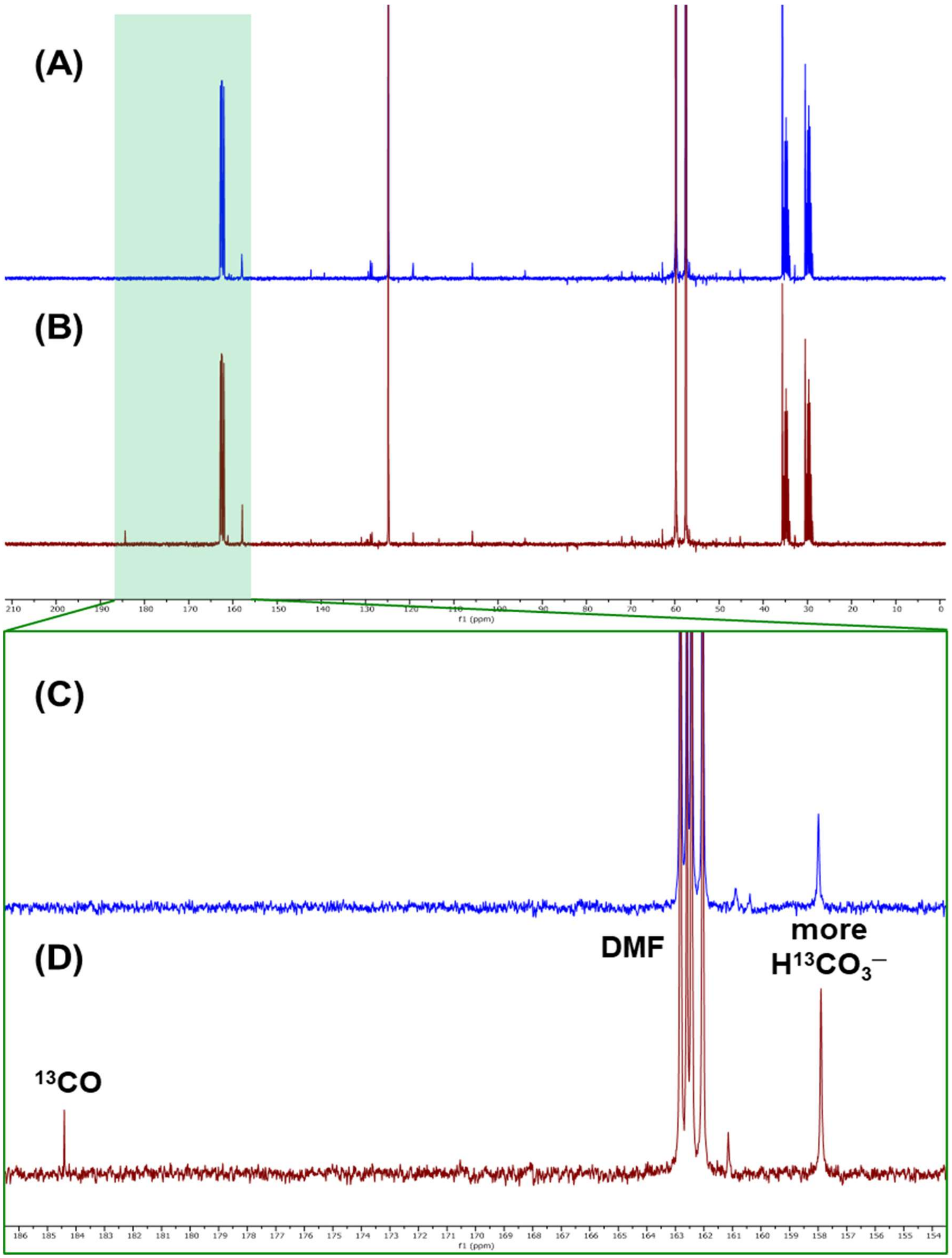

Figure S83. $^{13}C\{^{1}H\}$ NMR spectra (75 MHz, DMF-d_7) of photocatalytic reaction mixture before (A & C) and after 24 hours of blue light irradiation (B & D), where the reaction mixture was Re(bpy-C2-NPDI-NH_2) (30 μM), BIH (10 mM), and $^{13}CO_2$ (~0.9 atm) in a 5:1 DMF-d_7:TEOA mixture. Following CO_2 photocatalysis, a ^{13}CO resonance (δ 184 ppm) emerged and the intensity of the $H^{13}CO_3^-$ resonance (δ 158 ppm) increased, consistent with a disproportionation reaction.[16]

8. Evaluation of Re(bpy-C2-NPDI-NO_2)

Using our lab's standard copper catalyzed azide-alkyne cycloaddition (CuAAC) procedure, it was observed that Re(bpy-C2-NPDI-NH_2) was formed as a by-product during the synthesis of Re(bpy-C2-NPDI-NO_2). When sacrificial reducing agent, sodium ascorbate, was removed from the synthetic protocol, no Re(bpy-C2-NPDI-NH_2) was formed. This strongly hints towards the Re(bpy-C2-NPDI-NO_2) being converted into Re(bpy-C2-NPDI-NH_2) under reducing conditions, when a polar aprotic solvent (i.e. THF or DMF) and an adventitious proton source (i.e. H_2O or TFE) are present. Examining now the electrochemical behavior of Re(bpy-C2-NPDI-NO_2), there is a noticeable difference compared to the other Re(bpy-C2-NPDI-R) dyads.

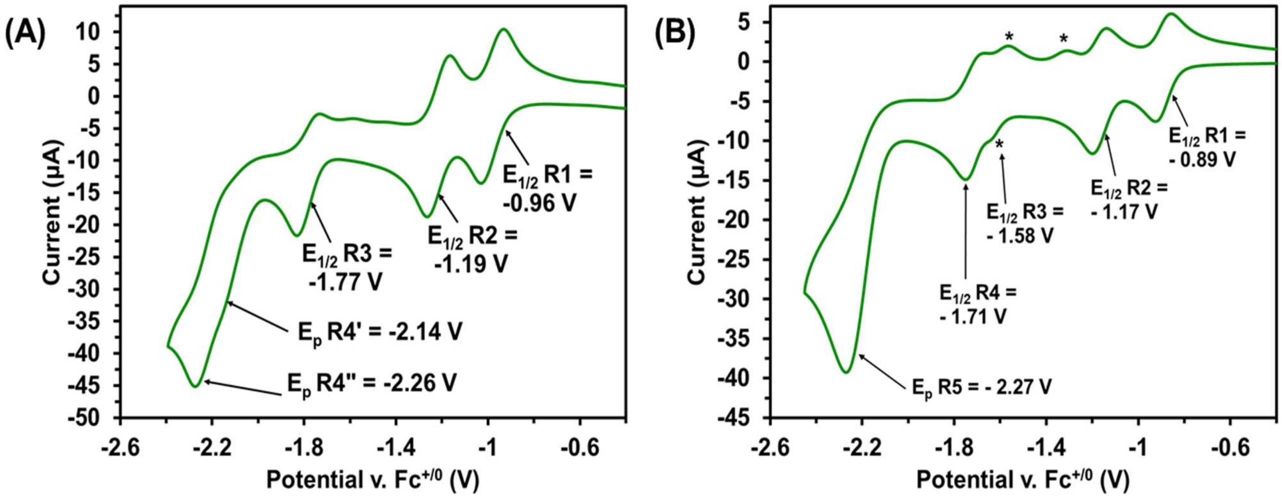

Figure S84. Comparing the cyclic voltammograms of Re(bpy-C2-NPDI-NO_2) in CH_2Cl_2 (A) and DMF (B) under an atmosphere of argon. All measurements were recorded at 100 mV/s with 0.1 M $TBAPF_6$ supporting electrolyte (WE = glassy carbon, CE = Pt-wire, RE = Ag-wire (CH_2Cl_2) or Ag/AgCl (DMF), and $Fc^{+/0}$ as internal reference standard).

Unlike all other Re(bpy-C2-NPDI-R) dyads, Re(bpy-C2-NPDI-NO_2) exhibited two concomitant redox processes at values more negative than -2.07 V. The redox event at E_p = -2.14 V is likely NPDI-NO_2 based because the Re(bpy)-based redox event (at E_p = -2.27 V) is consistent with all other Re(bpy-C2-NPDI-R) dyads. Another unique feature of this Re(bpy-C2-NPDI-NO_2) derivative is that under reducing conditions, the presence of adventitious H_2O in a polar aprotic solvent, like DMF, induces the evolution of one new redox event at E_p = -1.62 V (marked by *) on the cathodic scan. On the anodic return scan, there are two new redox processes at E_p = -1.54 V and -1.30 V, respectively.

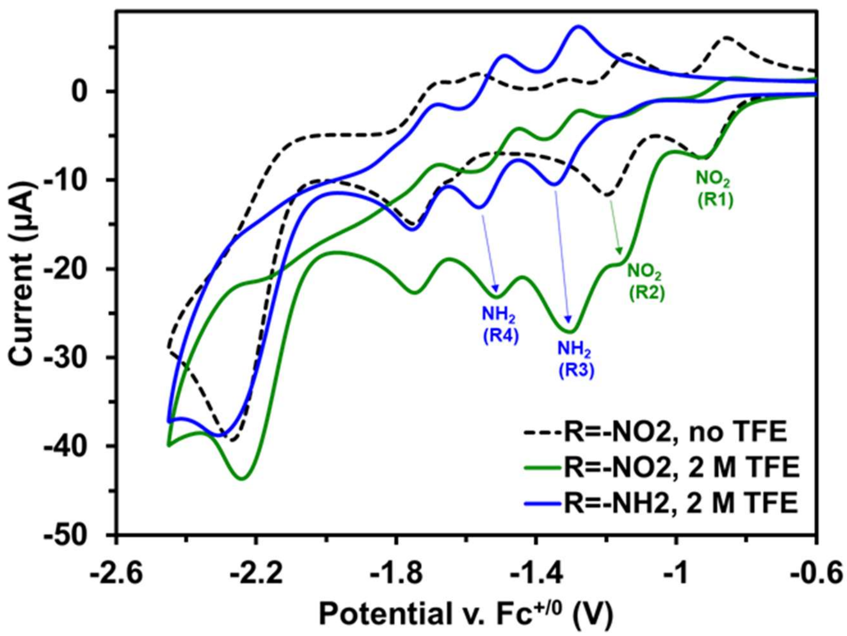

Figure S85. Overlaid cyclic voltammograms of Re(bpy-C2-NPDI-NO_2) under argon (black) with Re(bpy-C2-NPDI-NO_2) (green) and Re(bpy-C2-NPDI-NH_2) (blue) under CO_2 with 2 M TFE added. All measurements were recorded at 100 mV/s, under argon in DMF with 0.1 M $TBAPF_6$ supporting electrolyte (WE = glassy carbon, CE = Pt-wire, RE = Ag/AgCl, and $Fc^{+/0}$ as internal reference standard).

Relative to the initial trace under argon (black), the incremental addition of TFE under an atmosphere of CO_2 led to a CV current enhancement at the $NPDI^{2-/\bullet-}$ redox couple of Re(bpy-C2-NPDI-NO_2) ($E_{1/2}$ = -1.17V). As a result, two new reversible reduction features (at $E_{1/2}$ = -1.31 and -1.58 V) were observed prior to the $bpy^{\bullet-/0}$ ($E_{1/2}$ = -1.71 V) and $Re^{0/I}$ (E_p -2.27 V) redox processes. While initially it was believed that these new redox features signified dyad degradation, a closer inspection of these redox features shows excellent correlation to the $NPDI^{\bullet-/0}$ and $NPDI^{2-/\bullet-}$ reductions of Re(bpy-C2-NPDI-NH_2). This would strongly suggest that -NO_2 undergoes an *in-situ* conversion to -NH_2 under these catalytic conditions.

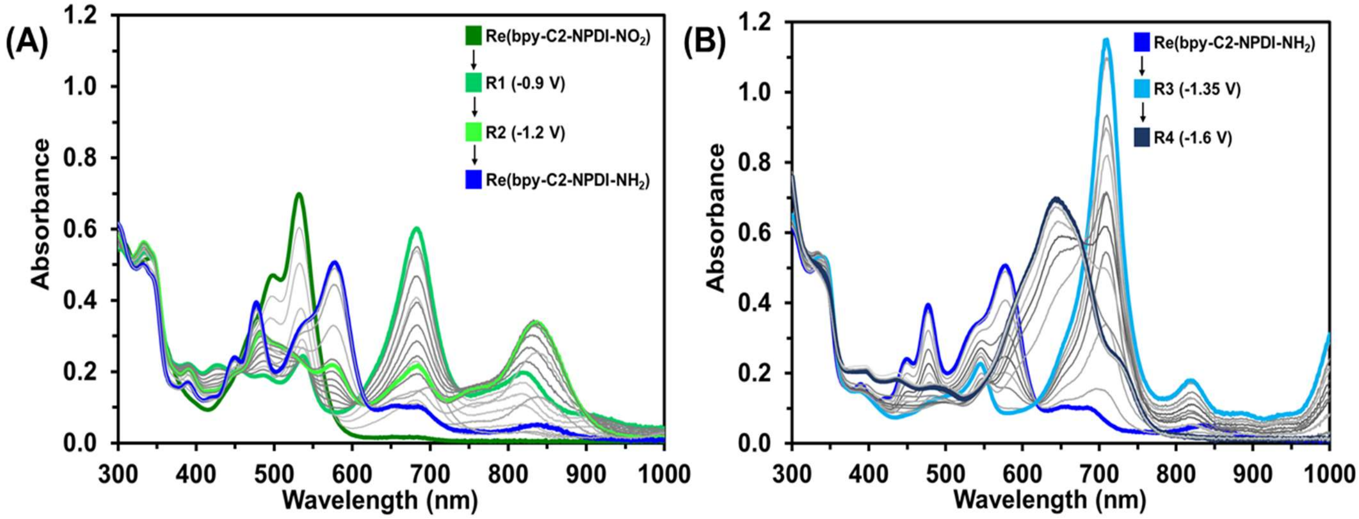

Figure S86. UV-vis-nIR SEC absorbance spectra of Re(bpy-C2-NPDI-NO_2), where R1 = -0.9 V, R2 = -1.2 V, R3 = -1.3 V, and R4 = -1.6 V. All experiments were performed in DMF with 0.1 M $TBAPF_6$ supporting electrolyte (WE = Pt-mesh, CE = Pt-wire, pseudo-RE = Ag-wire).

Unlike the other Re(bpy-C2-NPDI-R) dyads, which all displayed two transitions by UV-vis-nIR SEC, the Re(bpy-C2-NPDI-NO_2) derivative exhibited five unique electrochemical transitions. One electron reduction of Re(bpy-C2-NPDI-NO_2) resulted in the characteristic shift in λ_{max} from 534 nm to 686 nm, along with the emergence of one fine-structure absorbance band at 812 nm (turquoise). The two electron reduction of Re(bpy-C2-NPDI-NO_2) provides an intriguing scenario where $NPDI^{\bullet-}$ spectral features are rapidly replaced by absorption peaks at λ = 484, 585, and 839 nm (lime green). Before this species could be fully evolved, however, it is consumed to afford a species with strong spectral features at λ = 479, 539, and 578 nm (blue). The overall shape and position of these spectral features are identical to Re(bpy-C2-NPDI-NH_2). Moreover, the subsequent reductions this *in-situ* generated species resulted in the exact same UV-vis-nIR SEC spectral transitions measured for Re(bpy-C2-NPDI-NH_2) (Figure 4B). Therefore, these data strongly support our hypothesis that -NO_2 is *in-situ* converted to -NH_2 under the conditions necessary for CO_2 reduction catalysis.

9. References

1 J. D. B. Koenig, J. Willkomm, R. Roesler, W. E. Piers and G. C. Welch, *ACS Appl. Energy Mater.*, 2019, **2**, 4022–4026.

2 A. J. Bard and L. R. Faulkner, Electrochemical Methods, Electrochemical Methods: Fundamentals and Applications, 2nd Edition | Wiley: 2008.

3 M. E. Ahmed, A. Rana, R. Saha, S. Dey and A. Dey, *Inorg. Chem.*, 2020, **59**, 5292–5302.

4 M. L. Clark, P. L. Cheung, M. Lessio, E. A. Carter and C. P. Kubiak, *ACS Catal.*, 2018, **8**, 2021–2029.

5 E. Orgiu, J. George, J. A. Hutchison, E. Devaux, J. F. Dayen, B. Doudin, F. Stellacci, C. Genet, J. Schachenmayer, C. Genes, G. Pupillo, P. Samorì and T. W. Ebbesen, *Nat. Mater.*, 2015, **14**, 1123–1129.

6 J. R. Cann, C. Cabanetos and G. C. Welch, *Eur. J. Org. Chem.*, 2018, **2018**, 6933–6943.

7 Y. Yang, C. Fryer, J. Sharkey, A. Thomas, U. Wais, A. W. Jackson, B. Wilm, P. Murray and H. Zhang, *ACS Appl. Mater. Interfaces*, 2020, **12**, 27930–27939.

8 M. Stolte, T. Schembri, J. Süß, D. Schmidt, A.-M. Krause, M. O. Vysotsky and F. Würthner, *Chem. Mater.*, , DOI:10.1021/acs.chemmater.0c02115.

9 G. R. Fulmer, A. J. M. Miller, N. H. Sherden, H. E. Gottlieb, A. Nudelman, B. M. Stoltz, J. E. Bercaw and K. I. Goldberg, *Organometallics*, 2010, **29**, 2176–2179.

10 C. D. Windle, M. W. George, R. N. Perutz, P. A. Summers, X. Z. Sun and A. C. Whitwood, *Chem. Sci.*, 2015, **6**, 6847–6864.

11 Y. Kuramochi, Y. Fujisawa and A. Satake, *J. Am. Chem. Soc.*, 2020, **142**, 705–709.

12 P. Lang, M. Pfrunder, G. Quach, B. Braun-Cula, E. G. Moore and M. Schwalbe, *Chem. – Eur. J.*, 2019, **25**, 4509–4519.

13 F. Franco, C. Cometto, C. Garino, C. Minero, F. Sordello, C. Nervi and R. Gobetto, *Eur. J. Inorg. Chem.*, 2015, **2015**, 296–304.

14 D. R. Case, A. Spear, A. F. Henwood, M. Nanao, S. Dampf, T. M. Korter, T. Gunnlaugsson, J. Zubieta and R. P. Doyle, *Dalton Trans.*, 2021, **50**, 3479–3486.

15 N. T. L. Porte, J. F. Martinez, S. Hedström, B. Rudshteyn, B. T. Phelan, C. M. Mauck, R. M. Young, V. S. Batista and M. R. Wasielewski, *Chem. Sci.*, 2017, **8**, 3821–3831.

16 Y. Tamaki, K. Koike, T. Morimoto and O. Ishitani, *J. Catal.*, 2013, **304**, 22–28.
